# Selective oxidation of methane to C_2+_ products over Au-CeO_2_ by photon-phonon co-driven catalysis

**DOI:** 10.1038/s41467-024-51690-2

**Published:** 2024-08-30

**Authors:** Chao Wang, Youxun Xu, Lunqiao Xiong, Xiyi Li, Enqi Chen, Tina Jingyan Miao, Tianyu Zhang, Yang Lan, Junwang Tang

**Affiliations:** 1 Department of Chemical Engineering, University College London, London, WC1E 7JE UK; 2 Industrial Catalysis Center, Department of Chemical Engineering, Tsinghua University, Beijing, 100084 China; 3 Beijing Key Lab for Source Control Technology of Water Pollution, College of Environmental Science and Engineering, Beijing Forestry University, Beijing, 100083 P. R. China

**Keywords:** Photocatalysis, Heterogeneous catalysis

## Abstract

Direct methane conversion to high-value chemicals under mild conditions is attractive yet challenging due to the inertness of methane and the high reactivity of valuable products. This work presents an efficient and selective strategy to achieve direct methane conversion through the oxidative coupling of methane over a visible-responsive Au-loaded CeO_2_ by photon-phonon co-driven catalysis. A record-high ethane yield of 755 μmol h^−1^ (15,100 μmol g^−1^ h^−1^) and selectivity of 93% are achieved under optimised reaction conditions, corresponding to an apparent quantum efficiency of 12% at 365 nm. Moreover, the high activity of the photocatalyst can be maintained for at least 120 h without noticeable decay. The pre-treatment of the catalyst at relatively high temperatures introduces oxygen vacancies, which improves oxygen adsorption and activation. Furthermore, Au, serving as a hole acceptor, facilitates charge separation, inhibits overoxidation and promotes the C-C coupling reaction. All these enhance photon efficiency and product yield.

## Abstract (web summary)

To achieve high yield and selectivity of C_2+_ products from methane conversion, the authors report a photon-phonon co-driven catalytic process using CeO_2_ catalysts. Gold, as a co-catalyst, promotes C-C coupling and suppresses overoxidation

## Introduction

Methane, the main component of natural gas, has been used as a low-cost and clean fuel for heating. With large reserves of natural gas being discovered, the use of methane as a potential primary raw material for chemical synthesis becomes attractive. Industrially, methane upgrade is realised via two steps: an initial reforming procedure to generate synthesis gas, and a subsequent Fischer-Tropsch process to convert the synthesis gas into long-chain hydrocarbons^1,2^. Compared with the two-step process, the direct conversion of methane into high-value chemicals is not only scientifically viable but also economically cost-effective. Up to now, tremendous effort has been made to directly convert methane into methanol or multi-carbon products (C_2+_) via non-oxidative coupling of methane (NOCM), oxidative coupling of methane (OCM), and partial oxidation of methane^3–6^. However, due to the highly symmetrical molecular structure and high C-H bond energy, efficient and selective methane conversion always requires high temperatures (>600 °C) and/or expensive oxidants (e.g., H_2_O_2_ and H_2_SO_4_)^7–9^. In recent years, there has been a growing interest in the development of catalysts and reaction conditions for the direct conversion of methane under mild conditions. This is a challenging but promising area of research, with the potential to significantly reduce the cost and environmental impact of methane conversion.

Photocatalysis is an attractive approach to drive chemical reactions, and offers a promising solution for direct methane conversion. Instead of using heat, photocatalysis employs photons to drive reactions under mild conditions. Recent studies have shown that methane can be photocatalytically converted into mono-carbon (C_1_) oxygenates (e.g., CH_3_OH, CH_3_OOH, HCHO, etc.) by metal oxides loaded with co-catalysts in batch reactors^10–14^. Conversion of methane into C_2+_ chemicals is economically more profitable, but also technically more challenging. Nevertheless, photocatalytic methane upgrade in the absence of oxidants (NOCM) shows the potential to achieve C_2+_ products with a selectivity of ca. 95% using catalysts such as ZnO, TiO_2_ and Ga_2_O_3_, but the yield is very low (ca. 2 μmol h^−1^) due to the endothermic nature of the reaction^15–17^. For photocatalytic OCM, the presence of oxygen promotes the yield of C_2+_ in methane conversion. Our study has demonstrated the continuous conversion of methane into ethane and ethylene using a Pt-CuO_x_/TiO_2_ photocatalyst in the presence of oxygen^18^. An improved ethane production rate of 6.8 μmol h^−1^ with a moderate C_2_ selectivity of 60% was obtained. Later, a Ag-AgBr/TiO_2_ photocatalyst was developed, which increased the ethane yield to 35 μmol h^−1^ with a C_2+_ selectivity of 79% in a pressurised flow reaction system^19^. Additionally, a ZnO/TiO_2_ hybrid photocatalyst loaded with Au was reported to selectively produce ethane at a yield of 100 μmol h^-1^_,_ achieving a selectivity of 90%^20^. Very recently, PdCu alloy and Au, working as hole-acceptors, were loaded onto UV-responsive TiO_2_ to reduce the overoxidation process in photocatalytic OCM. A series of spectroscopic measurements were adopted to investigate the charge migration process^21,22^. It is worth noting that the photocatalysts in these systems are mainly TiO_2_-based semiconductors, which are only active under UV light and show poor capability for visible light harvesting. On the other hand, overoxidation leads to low selectivity towards C_2+_ chemicals, given that all products or intermediates generated in OCM are more reactive than methane itself. Thus, it is desirable to design photocatalysts that display excellent light harvesting and charge separation, efficient methane activation and effective co-catalysts to restrain overoxidation in methane conversion.

Ceria (CeO_2_), a rare earth metal oxide, has displayed great potential in thermocatalysis^23^. CeO_2_ has been used as a powerful oxidation catalyst in various reactions, including CO oxidation, NO_x_ removal, organics oxidation, etc., due to its high oxygen mobility and the multi-valence nature of Ce^24–26^. For instance, Pd@CeO_2_ supported on Al_2_O_3_ showed active and stable methane oxidation performance over a wide range of temperatures from 250 to 850 °C and was capable of completely converting methane into CO_2_ at 400 °C^27^. Additionally, CeO_2_ is a visible-light responsive semiconductor with an absorption edge of 400–500 nm depending on the size and morphology, making it a suitable candidate for visible-driven photocatalysis in applications such as environmental treatment^28,29^. For instance, CeO_2_ doped with Ru and modified by Au co-catalyst was used for CO_2_ methanation^30^. A one-pass CO_2_ conversion of 75% and a methane selectivity of 100% was achieved at a GHSV of 80,000 mL g^−1^ h^−1^ under visible light irradiation. Recently, an intertwined network of fibrous Rh#CeO_2_ composite was reported for the photocatalytic reforming of methane with carbon dioxide^31^. The high yield and selectivity originated from the efficient partitioning of charges by Rh modification. With careful catalyst design, CeO_2_ could harvest both UV and visible photons and serve as a high-efficiency photocatalyst for methane oxidation.

In this study, we designed and synthesised CeO_2_ photocatalyst modified with Au (Au-CeO_2_−300) for photocatalytic oxidation of methane. An efficient and stable ethane production at a yield of 755 μmol h^−1^ (15,100 μmol g^−1^ h^−1^) with a high C_2+_ selectivity of 98% has been achieved, which is also attractively stable (>120 h). It is found that pretreating the Au-CeO_2_ catalyst at 300 °C introduces a certain level of defects (e.g., Ce^3+^ and oxygen vacancies) in CeO_2_, which is beneficial for methane oxidation. Finally, the role of Au co-catalyst in charge separation, overoxidation and C-C coupling is thoroughly discussed.

## Results and discussion

**Fig. 1:**
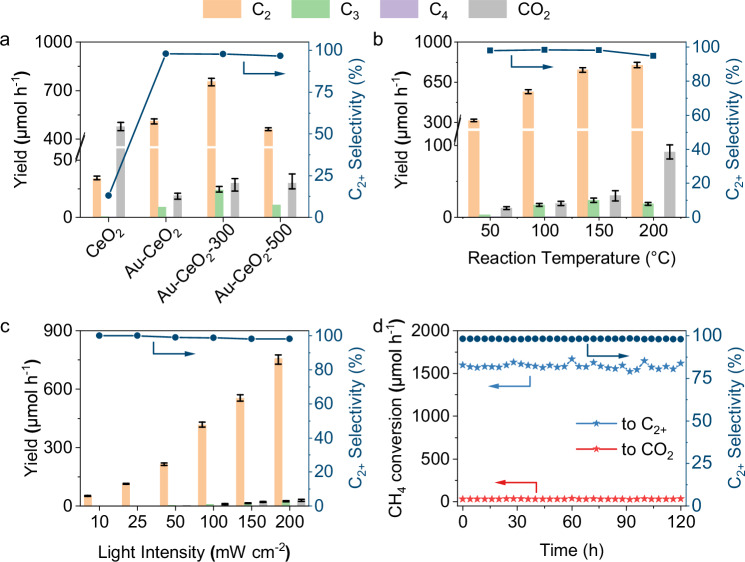
Photocatalytic oxidation of methane. **a** Product yields and C_2+_ selectivity over CeO_2_, Au-CeO_2_, Au-CeO_2_-300 and Au-CeO_2_−500; **b** Product yields and C_2+_ selectivity over Au-CeO_2_−300 operated at different temperatures; **c** Products yield and C_2+_ selectivity over Au-CeO_2_-300 under different light intensities; **d** Long-term test of methane conversion rate and C_2+_ selectivity over Au-CeO_2_-300. Error bars represent standard deviations calculated from the performance tests of the photocatalysts prepared in three different batches. Unless otherwise specified, the reaction conditions applied are 50 mg catalyst, methane to air = 200:1, GHSV = 480,000 mL h^-1^ g^-1^, Pressure = 5 bar, Temperature = 150 °C, 365 nm LED, light intensity = 200 mW cm^-2^.

The photocatalytic methane oxidation was tested in a pressurised flow reaction system^19^. No products are observed in the absence of light, catalyst, or methane (Supplementary Fig. 1). Upon irradiation under 365 nm LED, Au-CeO_2_−300 shows the capability to convert methane to C_2_ to C_4_ products (C_2+_, including C_2_H_6_, C_2_H_4_, C_3_H_8_, C_3_H_6_ and C_4_H_10_) and a small amount of CO_2_ in the presence of air (Fig. 1a, Supplementary Fig. 1 and Supplementary Table 1). When using pristine CeO_2_ as the photocatalyst, CO_2_ is detected as the main product at a yield of 478 μmol h^-1^ with the co-production of C_2+_ hydrocarbons. The selectivity of C_2+_ products is only 13% for CeO_2_ (Fig. 1a). A variety of metals and metal oxides were loaded onto CeO_2_ to investigate the effect of co-catalysts on the performance of methane oxidation. Pt, Ru, Pd and Rh dramatically improve the oxidation of CH_4_ to CO_2_ (Supplementary Fig. 2). The oxide co-catalysts, such as CoO_x_, MnO_x_, and CuO_x_ display improved C_2+_ selectivity and suppressed overoxidation (Supplementary Fig. 3). Among all the co-catalysts used, Au, when loaded onto CeO_2_, displays the highest yield and selectivity towards C_2+_ hydrocarbons, especially C_2_H_6_ (Supplementary Figs. 3 and 4). The production rate of CO_2_ is reduced from 478 to 30 μmol h^-1^. A C_2_H_6_ yield of 512 μmol h^-1^ with a high C_2+_ selectivity of 98% is obtained. Pre-treating Au-CeO_2_ at 300 °C in air leads to an increase in the yield of all products. The yield of C_2_H_6_ is further improved to 755 μmol h^-1^, while the high C_2+_ selectivity of 98% is not affected. The isotopic labelling experiments unambiguously prove that the produced C_2+_ and CO_2_ are from CH_4_ and O_2_ in the reactant (Supplementary Fig. 5).

The loading amount of Au on CeO_2_ was firstly optimised. As discussed above, the major product obtained over pure CeO_2_ was CO_2_, suggesting an intense overoxidation process. The product selectivity is shifted towards C_2+_ hydrocarbons from 13% to 79% even with 0.1 wt.% Au loading (Supplementary Fig. 6). The yield of C_2_H_6_ increases from 427 to 755 μmol h^-1^ as the Au loading increases from 0.1 wt.% to 1 wt.%. Further increasing the Au loading causes a decrease in the yield while an increase in the selectivity of C_2+_ products. This suggests that the co-catalyst Au plays an important role in either promoting C-C coupling or suppressing overoxidation in the methane oxidation process. However, excessive Au covers the surface of CeO_2_ and blocks light absorption, resulting in decreased photon efficiency. Pretreating Au-CeO_2_ at elevated temperatures can have a positive impact on the performance of the catalyst. Au-CeO_2_ was pre-treated in a muffles furnace from 200 to 500 °C. The optimised temperature for pre-treatment is 300 °C (catalyst denoted Au-CeO_2_-300, Supplementary Fig. 7). High temperatures such as 500 °C (Au-CeO_2_-500) cause a decrease in the yield of C_2_H_6_ from 755 to 459 μmol h^-1^. The actual Au amount in Au-CeO_2_, Au-CeO_2_-300, and Au-CeO_2_-500 are 0.83 wt.%, 0.85 wt.%, and 0.81 wt.%, respectively, as measured by inductively coupled plasma-atomic emission spectrometry.

To optimise the reaction conditions for the photocatalytic conversion of methane using Au-CeO_2_-300, various parameters are studied. The effect of the reaction pressure on the activity of the catalyst was firstly studied. Increasing the reaction pressure from 1 bar to 5 bar leads to an increase in the yield of all products (Supplementary Fig. 8). The yield of C_2_H_6_ increases from 447 to 755 μmol h^-1^, while the selectivity towards C_2+_ is hardly affected. The product yields under 6 and 7 bar are similar to that achieved under 5 bar. Thus, 5 bar is selected as the optimised pressure. Next, the methane-to-air ratio is studied at a constant gas hourly space velocity (GHSV) of 480,000 mL h^-1^ g^-1^ using Ar as a balance. When O_2_ is absent in the reaction atmosphere, a low C_2_H_6_ yield of 71 μmol h^-1^ is obtained (Supplementary Fig. 9). As the concentration of air in the reactant increases, the yields of all products are improved. This indicates that oxygen gas can have a positive effect on photocatalytic methane conversion. However, the yield of CO_2_ surges from 30 to 124 μmol h^-1^ when the methane-to-air ratio changes from 200:1 to 12:1 with the C_2_H_6_ yield slightly increased from 755 to 859 μmol h^-1^. Overall, O_2_ is indispensable in photocatalytic OCM, however, too much O_2_ causes overoxidation of CH_4_ to CO_2_. To achieve both high yield and high selectivity of the high-value C_2+_ products, the methane-to-air ratio of 200:1 is chosen for subsequent studies. Another key factor is the reaction temperature, which would likely promote photocatalysis, i.e. the concept of photon-phonon co-driven catalysis. Au-CeO_2_-300 was then tested at temperatures ranging from 50 to 200 °C to investigate the coupling effect of thermal catalysis with photocatalysis (Fig. 1b). When the reaction temperature increases from 50 to 150 °C, the yields of C_2_H_6_ more than doubles, increasing from 317 to 755 μmol h^-1^ while the selectivity to C_2+_ hydrocarbons remains nearly identical at 98%. CO_2_ production also nearly doubles. The yield of C_2_H_6_ at 200 °C slightly increases to 792 μmol h^-1^, while the CO_2_ production is more than tripled (91 μmol h^−1^) compared with that at 150 °C. Thus, the optimised reaction condition for the photon-phonon co-driven catalysis is 150 °C and 5 bar. The product yield of Au-CeO_2_-300 at higher temperatures from 150 to 200 °C was also measured in dark (Supplementary Fig. 10). Only a trace amount of CO_2_ is detected at 200 °C. Thus, it can be concluded that the selective catalytic conversion of methane into C_2+_ products is not driven by heat only while the catalytic performance of Au-CeO_2_-300 at different temperatures (Fig. 1b) again suggests coupling photocatalysis with thermocatalysis can dramatically enhance the yield of C_2+_ products.

Moreover, the effect of GHSV was also studied. It is found that as the GHSV increases from 160,000 to 480,000 mL h^-1^ g^-1^, the yield of all products and the selectivity towards C_2+_ products increase (Supplementary Fig. 11). High GHSV causes reduced detention time of reactants on the catalyst bed, which is beneficial for the suppression of overoxidation but decreases overall methane conversion. Furthermore, the photocatalytic methane oxidation was measured under various light intensities from 10 to 200 mW cm^-2^. The product yield is approximately in a linear relationship with the light intensity (Fig. 1c), indicating that photo-generated charge carriers have the high utlisation efficiency. Since CeO_2_ is a visible-light responsive photocatalyst, Au-CeO_2_-300 was also tested under other light sources, including a Xe lamp (visible light, λ > 420 nm) and a blue LED (450 nm), and a Xe lamp equipped with a λ > 475 nm and λ > 550 nm filter (Supplementary Fig. 12). As shown in Supplementary Fig. 13, the C_2_H_6_ yields under visible light and blue LED are 158 and 57 μmol h^-1^, respectively, even higher than most of the reported results measured under strong UV light (Supplementary Table 2). The photocatalytic methane conversion performance of Au-CeO_2_-300 follows the absorption spectrum of CeO_2_ (Supplementary Figs. 11 and 12). Thus, the photo-generated charges by CeO_2_ are the driving force for the conversion of methane to C_2+_ products. No products are detected over Au-TiO_2_ and Au-ZnO under >420 nm irradiation (Supplementary Fig. 14). This shows the superiority of CeO_2_ as a visible-responsive photocatalyst and excludes the contribution of the plasmonic effect of Au to the activity under visible irradiation. Overall, the yield of C_2_H_6_, the main product, is as high as 755 μmol h^-1^ and the selectivity towards C_2+_ molecules is 98% under the optimised conditions. This performance is the record among all reported photocatalytic methane conversion reactions (Supplementary Table 2) and is >7 times higher than the recently reported benchmark photocatalyst Au-TiO_2_/ZnO^20^. The apparent quantum efficiency of Au-CeO_2_-300 at 365 nm is calculated to be 12%. In addition, the potential of Au-CeO_2_-300 for oxidative coupling of methane under simulated solar irradiation was also measured using a Xe lamp equipped with an AM 1.5 filter (100 mW cm^-2^) without external heating. The production rates of C_2_H_6_, C_2_H_4_, C_3_H_8_, and C_4_H_10_ are 104, 0.2, 4.5 and 51 μmol h^-1^, respectively, suggesting the potential of the current catalyst for solar driven methane conversion although it is less productive than the above photon-phonon co-driven catalysis.

The durability and stability of Au-CeO_2_-300 were assessed by conducting long-term tests under the optimised reaction conditions for 120 h (Fig. 1d). Methane is continuously converted into C_2+_ products at a rate ranging from 1521 to 1665 μmol h^-1^ with the C_2+_ selectivity kept at 98%. The Au-CeO_2_-300 photocatalyst after running for 120 h was then characterised by X-ray diffraction (XRD), transmission electron microscopy (TEM), and ultraviolet-visible diffuse reflectance spectroscopy (UV-Vis DRS). The results indicate that the phase structure, morphology band structure of CeO_2_, and the particle size of the co-catalyst Au remained unchanged in Au-CeO_2_-300 even after 120 h of reaction (Supplementary Figs. 15–17). These findings prove that Au-CeO_2_-300 is a robust and stable photocatalyst and can efficiently and selectively convert methane into C_2+_ hydrocarbons for an extended period under the current reaction conditions.

**Fig. 2:**
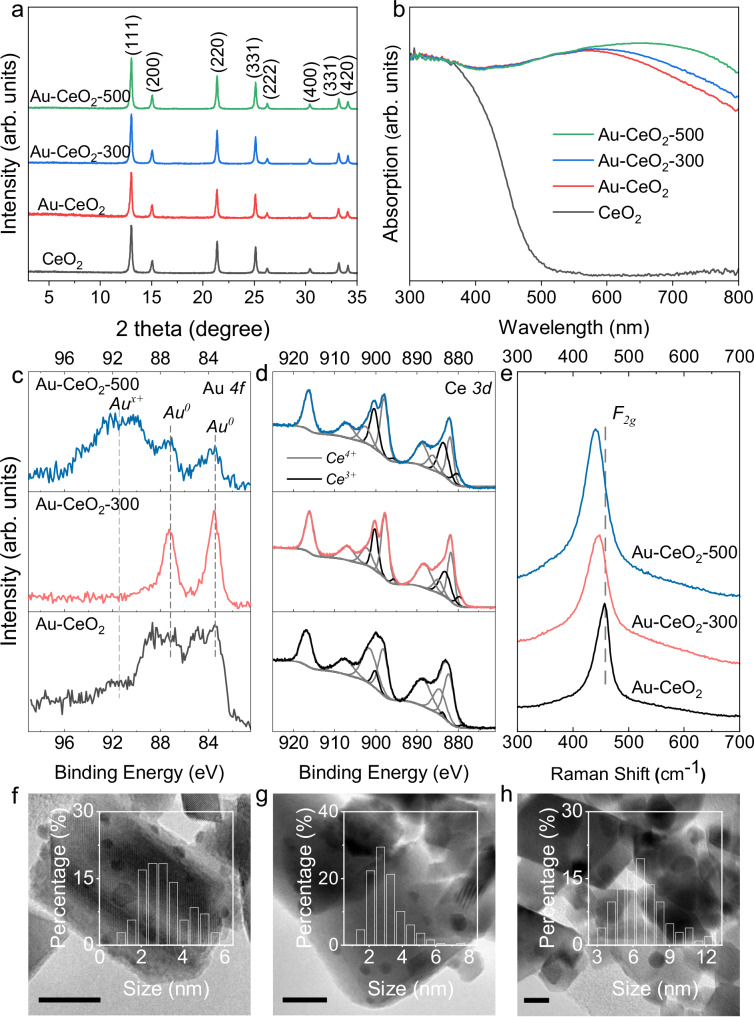
Catalyst characterisation. **a** XRD and (**b**) UV-Vis DRS spectra of CeO_2_, Au-CeO_2_, Au-CeO_2_-300 and Au-CeO_2_−500; **c** Au *4* *f*, (**d**) Ce *3d* XPS and (**e**) Raman spectra of Au-CeO_2_, Au-CeO_2_−300 and Au-CeO_2_-500; TEM images of (**f**) Au-CeO_2_, (**g**) Au-CeO_2_-300 and (**h**) Au-CeO_2_-500, insets show the size distribution of Au particles, scale bar: 10 nm.

A thorough characterisation of the catalysts used in this study was conducted. XRD analysis of CeO_2_ shows that all peaks are attributed to the cubic fluorite phase (JCPDS # 34-0394) of ceria (Fig. 2a). After the loading of Au, no additional peaks for Au are detected, likely due to the small particle size and/or high dispersion of the co-catalyst. Similar diffraction features are observed for Au-CeO_2_ after calcination at 300 °C and 500 °C, indicating that the lattice structure of the photocatalyst remains unchanged after the pre-treatment. The UV-Vis DRS spectrum of CeO_2_ shows an absorption edge of 495 nm, corresponding to an optical bandgap of 2.5 eV (Fig. 2b), which is slightly smaller than the reported probably due to partially reduced Ce^4+^ in the catalyst^32^. When 1 wt. % of Au is loaded on CeO_2_, a strong absorption is observed across the whole visible range. The absorption in UV and blue regions (300-450 nm) is mainly contributed by CeO_2_. The band at 600 nm is ascribed to the plasmonic effect of metallic Au nanoparticles on the surface of CeO_2_^20^. Due to the closely packed Au nanoparticles on the CeO_2_ support, a strong scattering in the visible range is observed^33^. When the Au loading amount is reduced to 0.1 wt.%, where the scattering is reduced, two absorption features originating from CeO_2_ and plasmonic Au are clearly observed (Supplementary Fig. 18). The strong visible absorption is also reflected by the dark colour of Au-CeO_2_ (Supplementary Fig. 19). A red shift of the plasmonic absorption is found after Au-CeO_2_ is calcined at higher temperatures, particularly for Au-CeO_2_-500, indicating that the pre-treatment at an extra high temperature may have caused growth or aggregation of the co-catalyst Au^34^.

X-ray Photoelectron Spectroscopy (XPS) was performed to investigate the relation between the chemical states and the performance of photocatalysts. High-resolution Au 4 *f* spectra suggest that metallic Au is the main species of Au in Au-CeO_2_ and Au-CeO_2_-300 (Fig. 2c), while a large proportion of oxidised Au is found in Au-CeO_2_-500. This indicates that the calcination of Au-CeO_2_ at 500 °C in air caused the oxidation of Au into Au_2_O_3_^35^. The Ce *3d* spectra show that Ce^4+^ and Ce^3+^ co-exist in CeO_2_, Au-CeO_2_, Au-CeO_2_-300 and Au-CeO_2_-500 (Supplementary Fig. 20 and Fig. 2d)^36^. The bands in the Ce *3d* XPS spectrum of Au-CeO_2_-300 display a clear shift towards lower binding energies compared to that of Au-CeO_2_, which indicates a partial reduction of Ce^4+^ to Ce^3+^ (Supplementary Fig. 21). The proportion of Ce^3+^ and Ce^4+^ in the catalysts is calculated based on the integrated area of the corresponding bands and summarised in Supplementary Table 4. It shows that the Ce^3+^ contents in CeO_2_ and Au-CeO_2_ are relatively low, 8.5% and 4.8%, respectively. After calcination at 300 °C, 24.6% of Ce^3+^ is found in Au-CeO_2_-300. The Ce^3+^ content increases to 28.9% when the pre-treatment temperature reaches 500 °C. EPR was used to further investigate the valence change of cerium after the pre-treatment of Au-CeO_2_ (Supplementary Fig. 22). The strongest band with a g value of 1.96 originates from the Ce^3+^-O^-^-Ce^4+^ sites in CeO_2_^37^. The intensity of this band increases with the pre-treatment temperature, suggesting more Ce^4+^ is reduced to Ce^3+^ after pre-treating at higher temperatures. The signals at g values of 1.88, 1.93, 2.08 and 2.14 are also related to Ce^3+^ in the samples^38^.

The generation of Ce^3+^ in CeO_2_ is often accompanied by the formation of oxygen vacancies (O_V_)^39^. Raman spectroscopy was used to probe the formation of O_v_ in Au-CeO_2_ after pre-treatment. The *F*_*2g*_ mode in the Raman spectrum of CeO_2_ originates from the symmetrical stretching vibration of O^2-^ around Ce^4+^ and is sensitive to defects such as O_V_^40,41^. The F_2g_ peak shifts from 457 cm^-1^ for Au-CeO_2_ to 448 cm^-1^ for Au-CeO_2_-300, and further to 441 cm^−1^ for Au-CeO_2_-500 (Fig. 2e). The redshift of *F*_*2g*_ mode is related to the presence of O_V_ in the material^40,42^. O_V_ in CeO_2_ has been reported to promote the adsorption and activation of oxygen gas in catalytic oxidation reactions^42^. To exclude the effect of Au particle change during the calcination on the adsorption of O_2_, CeO_2_ was calcined at 300 °C and 500 °C to obtain CeO_2_-300 and CeO_2_-500 and measured for oxygen adsorption. The results indicate that CeO_2_-300 and CeO_2_-500 display more oxygen uptake than CeO_2_ (Supplementary Fig. 23). The O 1 *s* XPS spectra also reveal that after the pre-treatment, a large amount of chemically adsorbed oxygen-containing species are detected on the surface of the Au-loaded CeO_2_ (Supplementary Fig. 24). The O_2_^-^ trapping EPR experiment indicates that the photocatalytic oxygen activation capability of CeO_2_ is improved by the existence of O_v_ (Supplementary Fig. 25). Improved photocatalytic methane oxidation is also observed for CeO_2_-300 and CeO_2_-500 compared with CeO_2_ (Supplementary Fig. 26), in agreement with the Raman, O_2_ adsorption and O_2_^-^ trapping EPR results. Pre-treatment of Au-CeO_2_ introduces O_v_ into CeO_2_, which promotes the adsorption and activation of O_2_ and is beneficial for photocatalytic methane oxidation.

The TEM images of Au-CeO_2_, Au-CeO_2_-300 and Au-CeO_2_-500 reveal that the Au nanoparticles are well-dispersed on CeO_2_ (Fig. 2f–h). No significant change in the crystals of CeO_2_ is observed after pre-treatment (Supplementary Fig. 27). The Au size distribution indicates that the Au nanoparticles on Au-CeO_2_ are between 1 and 6 nm, while >83% of Au on Au-CeO_2_-300 displays particle size from 2 to 4 nm. It suggests that the Au nanoparticles smaller than 2 nm grow into sizes of 2–4 nm after pre-treatment at 300 °C. The slight growth of Au nanoparticles at 300 °C is verified by calcination Au-CeO_2_-Na with smaller Au nanoparticles at 300 °C (Supplementary Figs. 28–30). The wide size distribution of Au in Au-CeO_2_ also explains its widened Au *4* *f* XPS spectrum compared to that of Au-CeO_2_-300 (Fig. 2c). However, increasing the calcination temperature to 500 °C leads to the intensive growth of Au nanoparticles to ~6 nm. The TEM images did not reveal any oxidised Au surface, which could be due to the reduction of the Au_2_O_3_ to the metallic state by the electron beam during TEM measurement.

Combining the results obtained from UV-Vis DRS, Raman, XPS, EPR, and TEM analysis, it can be concluded that pre-treating Au-CeO_2_ at 300 °C induces a change of the catalyst, generating Ce^3+^ sites and oxygen vacancies that improve oxygen adsorption and activation, and cause slight growth of the Au nanoparticles. However, it causes the intensive growth and oxidation of the co-catalyst Au when the pre-treatment temperature is further increased to 500 °C, leading to reduced catalytic performance.

The yield and product selectivity of a photocatalytic system are often affected by two important factors, which are charge separation and surface reaction. Firstly, the charge separation and migration process of photocatalytic methane oxidation by CeO_2_ and Au-CeO_2_-300 was investigated by photo-induced absorption (PIA) spectroscopy. An in situ UV-Vis DRS system was developed to measure the reflectance of the photocatalyst in dark or under light irradiation in various reaction atmospheres (Supplementary Fig. 31)^43^. The PIA can be calculated by the following equation:

1 $$\varDelta {{Abs}}=\frac{{R}_{da{rk}}-{R}_{{lig}ht}}{{R}_{d{ark}}}\times 100\%$$

Where R_dark_ and R_light_ are the reflectances measured in dark and under light conditions, respectively.

**Fig. 3:**
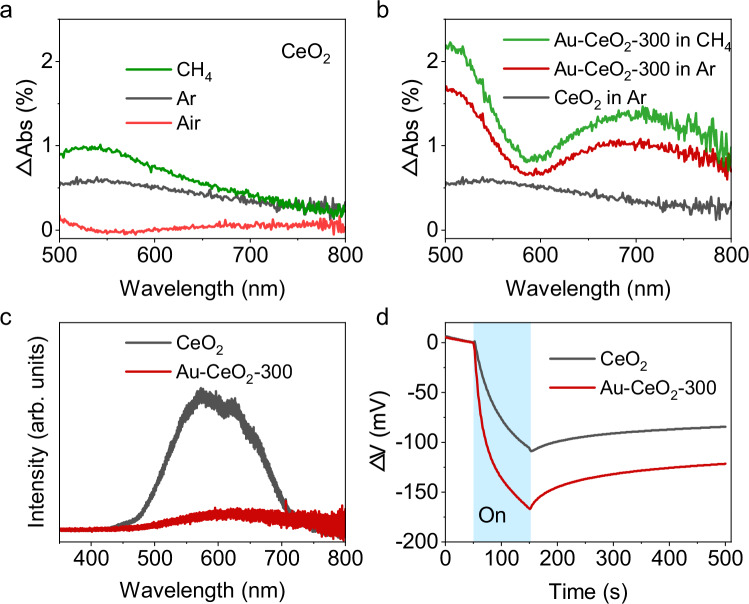
Charge separation and migration. **a** PIA spectra of CeO_2_ in Ar, air and methane; **b** PIA spectra of CeO_2_ in Ar, Au-CeO_2_-300 in Ar and Au-CeO_2_-300 in methane; **c** PL spectra and (**d**) OCVD plots of CeO_2_ and Au-CeO_2_-300.

To ensure the reliability of the experimental setup and to eliminate the possibility of any interference from the irradiation source, a reference sample, BaSO_4_ was tested under an Ar atmosphere. The reflectance spectra of BaSO_4_ in dark and under light irradiation are found to overlap well with each other (Supplementary Fig. 32a). It suggests that no interference with the measurement is caused by the UV-LED as no PIA is detected for BaSO_4_ (Supplementary Fig. 32b). The PIA spectrum of CeO_2_ was subsequently observed under an Ar atmosphere. An evident difference between the reflectance of CeO_2_ in dark and under light irradiation is observed (Supplementary Fig. 33a). The PIA spectrum of CeO_2_ suggests that an absorption across the entire visible range is generated when CeO_2_ is illuminated under a 365 nm LED (Supplementary Fig. 33b). Transient absorption studies reported that excited CeO_2_ displays a positive absorption from 500 to 800 nm, which originates from the electron transition from the conduction band (CB) to the higher energy levels close to the CB (Supplementary Fig. 34a)^44^. A series of light on-off measurements of reflectance at 500, 600 and 700 nm show the repeatable decrease in the reflectance of CeO_2_ upon irradiation, indicating the PIA signals are highly reproducible (Supplementary Fig. 35). The PIA spectra of CeO_2_ are further investigated in air and methane atmospheres (Fig. 3a). The PIA of CeO_2_ is quenched in the air atmosphere. Oxygen gas in air is a powerful electron scavenger and can be reduced by CB electrons of CeO_2_^45,46^. In other words, most CB electrons from CeO_2_ are consumed by oxygen gas when air is introduced, resulting in the reduced charge transition between the CB of CeO_2_ and its closer energy levels (Supplementary Fig. 34b). On the other hand, in the methane atmosphere, an increase in the PIA spectrum is observed. This is ascribed to the fact that the function of methane is reversed to oxygen gas, ie. CH_4_ is a photohole acceptor, which leads to improved charge separation and abundant photoelectrons at the CB of CeO_2_ (Supplementary Fig. 34c). Consequently, the enhanced charge transition related to CB electrons is detected. XPS VB spectrum indicates CeO_2_ display a VB potential of 2.2 V vs. NHE (Supplementary Fig. 36), which is sufficient for methane activation^47^. Considering the bandgap of CeO_2_ is 2.5 eV, the CB potential is calculated to be -0.3 V vs. NHE, which is more negative than that for the oxygen reduction reaction (-0.16 V vs. NHE)^48^. Therefore, it can be concluded that during photocatalytic methane oxidation by CeO_2_, electrons and holes are generated upon light irradiation, and then are consumed by oxygen gas and methane, respectively.

Subsequently, the PIA spectrum of Au-CeO_2_-300 was measured in Ar to study the role of Au in charge separation and migration. The PIA spectrum of Au-CeO_2_-300 exhibits two distinct features (Fig. 3b). Firstly, a significant increase in the PIA is detected over Au-CeO_2_-300 compared with CeO_2_, suggesting that Au plays a similar role as methane when CeO_2_ is excited under UV irradiation. In other words, when Au-CeO_2_-300 is irradiated under UV, the photoholes transfer from the VB of CeO_2_ to Au and Au serves as a hole acceptor in Au-CeO_2_-300. Secondly, a trough at ca. 600 nm in the PIA spectrum is observed, which is caused by the ground-state bleaching of plasmonic Au^49,50^. A further increment in PIA is observed when Au-CeO_2_-300 is measured in methane. This indicates that the oxidation potential of photo-holes on Au is still capable of methane activation. With the co-existence of Au and methane, more CB electrons in CeO_2_ could be promoted to higher levels by the probe source. Therefore, the highest PIA is observed. To prove the reliability of this result, Pt, which is well-documented as an electron acceptor and co-catalyst in photocatalysis, was loaded on CeO_2_ and measured for PIA (Supplementary Fig. 37). After loading Pt onto CeO_2_, the PIA signal is quenched, indicating again that the PIA observed is attributed to photoelectrons in CeO_2_ as the photo-generated electrons transfer from the CB of CeO_2_ to Pt. This result confirms the conclusion above that Au works as a hole acceptor. The opposite results in the product distribution of methane conversion by Pt- or Au-modified CeO_2_ also suggest that Pt and Au play different roles in photocatalytic methane oxidation (Supplementary Fig. 38). In situ Au 4 *f*  XPS spectra of Au-CeO_2_-300 were measured to further probe the function of Au in charge separation (Supplementary Fig. 39). A positive shift of 0.3 eV is observed for the Au 4 *f* XPS bands of Au-CeO_2_-300 under light irradiation compared with that in dark. This suggests that under light irradiation, the transfer of holes from CeO_2_ to Au is detected.

Density functional theory (DFT) calculations are also performed to simulate the charge migration process. Au-CeO_2_-300 is simulated by loading a Au_9_ cluster onto the (111) surface of CeO_2_ with Ce^3+^ and O_V_s (Supplementary Figs. 40 and 41). The Density of States results show that Au 5d orbitals partially overlap with O 2p in Au-CeO_2_-300, suggesting that Au possibly works as an electron donor (Supplementary Fig. 42). The charge density difference clearly indicates the decrease in the electron density of Au and the increase in the electron density of CeO_2_ (Supplementary Fig. 43a), which means an electron transfer process from Au to CeO_2_. This has provided direct theoretical evidence that Au works as a hole acceptor in Au-CeO_2_-300. Similar charge behaviour is also observed over Au_9_-CeO_2_ in the absence of O_V_ (Supplementary Fig. 43b). The results obtained from the DFT calculation are in good agreement with the experimental analysis.

To gain further insights into the role of Au in charge separation, photoluminescence (PL) spectroscopy was conducted using a 325 nm UV laser as the excitation source. CeO_2_ exhibits a strong fluorescence centred at ca. 600 nm (Fig. 3c). In contrast, the PL spectrum of Au-CeO_2_-300 is quenched, indicating that the radiative charge recombination is significantly reduced after the loading of Au on CeO_2_. A red shift of the PL spectrum is observed after Au-CeO_2_ is pre-treated at 300 and 500 °C (Supplementary Fig. 44). This likely results from the recombinations related to the interband levels caused by the defects (e.g., Ce^3+^ and O_V_s) formed during pre-treatment, which is consistent with the XPS analysis. Electrochemical open circuit voltage decay (OCVD) is another widely used method to study the charge separation efficiency of semiconductors^51^. When irradiated by a Xe lamp, a negative photovoltage is observed over both CeO_2_ and Au-CeO_2_-300 (Fig. 3d), which arises from the separation of electrons and holes in the semiconductor electrodes. The accumulated photovoltage values on CeO_2_ and Au-CeO_2_-300 after irradiation for 100 s are 109 and 151 mV, respectively, indicating that Au can enhance the separation of photo-generated electrons and holes in CeO_2_ by nearly 50%. Therefore, the following conclusions can be drawn from the charge separation and migration analysis. First, photo-generated electrons react with oxygen gas while holes activate methane during photocatalytic methane oxidation. Second, Au acts as a hole acceptor in Au-CeO_2_-300 and can promote charge separation. Third, the photoholes transferred from the VB of CeO_2_ to Au are sufficiently oxidative for methane activation.

**Fig. 4:**
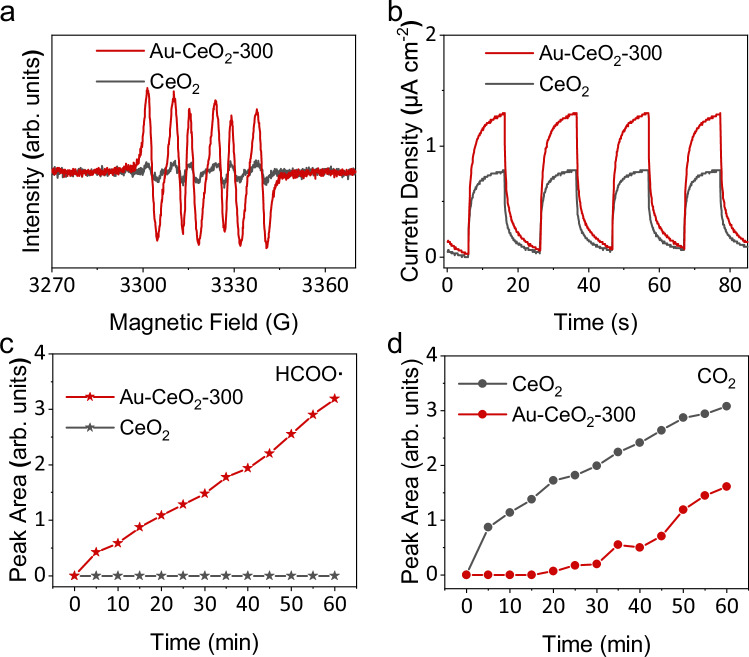
Surface chemistry investigations. **a** EPR spectra of CeO_2_ and Au-CeO_2_-300 for O_2_^-^ trapping; **b** Transient photocurrent response of CeO_2_ and Au-CeO_2_-300 at the bias potential of 0.3 V vs. Ag/AgCl; Evolution of (**c**) HCOO**·** species and (**d**) CO_2_ during the in situ DRIFTS measurement of CeO_2_ and Au-CeO_2_-300 (methane to air = 200:1).

After the charge separation and migration, the surface reactions take place, which involve reactant adsorption and activation, radical generation and transformation, product formation, and unexpected overoxidation^52^. The oxygen reduction reaction is an important half-reaction in all photocatalytic reactions with oxygen gas as the oxidant. The adsorption of O_2_ on CeO_2_ has been investigated and discussed (Supplementary Fig. 23). After adsorption on the catalyst surface, O_2_ reacts with photoelectrons, resulting in the generation of surface superoxide radicals (O_2_^-^). Electron paramagnetic resonance (EPR) was thus used to monitor the production of O_2_^-^ radicals with 5,5-dimethyl−1-pyrroline N-oxide (DMPO) as the spin-trapping reagent. After irradiation for 30 s, an EPR signal with six distinct peaks is obtained (Fig. 4a), resulting from the coupling product of O_2_^-^ radicals and DMPO. Au-CeO_2_-300 produces a much higher level of O_2_^-^ than CeO_2_ because of its high charge separation capability. However, the electrochemical oxygen reduction LSV results indicate that Au does not directly improve the oxygen reduction capability of CeO_2_, rather different from Pt (Supplementary Fig. 45). The photocatalytic performance of CeO_2_ under an oxygen-rich environment displays a different trend in the product selectivity compared with Au modification, which also indicates that Au does not improve O_2_^-^ formation via working as an electron acceptor like Pt (Supplementary Fig. 46). The LSV and EPR measurements indicate that Au promotes charge separation by working as a hole acceptor, which is beneficial for methanol oxidation, and indirectly favourable for superoxide radical production from oxygen reduction. The formed superoxide radicals play two main roles in photocatalytic methane oxidative coupling. Firstly, it combines with protons generated from CH_4_ oxidation half-reaction by photo-holes, to clear the surface of the catalyst for subsequent methane activation. Secondly, it reacts with the intermediates formed during methane oxidation, resulting in the production of CO_2_. No EPR signals are detected for either CeO_2_ or Au-CeO_2_-300 in dark (Supplementary Fig. 47), indicating O_2_^-^ radicals are formed from the reaction of oxygen gas with photo-generated electrons. Oxygen exchange between the oxygen gas and the lattice oxygen of CeO_2_ is also detected during photocatalytic methane oxidation by Au-CeO_2_-300 (Supplementary Fig. 48), which is similar as the reported^20,53^.

The other half reaction is the oxidation reaction of methane by photoholes. The adsorption of methane on CeO_2_ and Au-CeO_2_-300 was simulated using DFT calculations (Supplementary Fig. 49). The results show that although the adsorption of CH_4_ on both CeO_2_ and Au-CeO_2_-300 surfaces is weak, which could be ascribed to the highly symmetric structure of the molecule, the adsorption energy of CH_4_ on the Au-CeO_2_-300 surface is 0.11 eV more negative than that on the CeO_2_ surface, indicating that Au can slightly improve the adsorption of CH_4_ on the CeO_2_ surface, which is beneficial for the subsequent methane activation reaction. In situ DRIFTS reveal that the adsorption of methane can be improved by photo-irradiation (Supplementary Fig. 50). Activation of methane by photo-holes to generate CH_3_· is observed by in situ DRIFTS in pure CH_4_ (Supplementary Fig. 51). Under light irradiation, two bands at 2845 and 2880 cm^−1^ are detected over both CeO_2_ and Au-CeO_2_-300, which are ascribed to the symmetric and asymmetric stretching vibration of C-H bond in CH_3_**·** species^54^. The intensity of CH_3_**·** in the spectrum of Au-CeO_2_-300 is relatively stronger than that in CeO_2_, indicating that Au could greatly promote the methane activation rate or CH_3_**·** formation rate, which is also beneficial for the coupling of CH_3_**·** to C_2_H_6_. DFT calculation also suggests that a reduced energy barrier is achieved for methane activation over Au-CeO_2_-300 compared with CeO_2_ (Supplementary Fig. 52). Another feature in the DRIFTS spectra is the band located at 2947 cm^−1^, originating from the CH_3_O**·** species (Supplementary Fig. 51)^55^. CH_3_**·** is the first intermediate formed in the overoxidation process. The oxygen source in CH_3_O**·** is the lattice oxygen of CeO_2_ as the colour of both CeO_2_ and Au-CeO_2_−300 changes after the in situ DRIFTS measurement (Supplementary Fig. 53). The oxidation capability of the catalysts is also investigated by photo-electrochemical oxidation reaction. The solubility of methane is low in most electrolytes and the interaction of methane with the electrode surface is rather weak. Thus, photoelectrochemical water oxidation is used to investigate the oxidation capability of the photocatalysts, considering water oxidation requires a similar potential as methane activation^52^. Au-CeO_2_-300 shows a higher photocurrent than CeO_2_ across the entire potential window of the linear sweep voltammetry plot (Supplementary Fig. 54), indicating that more water molecules are activated and oxidised on Au-CeO_2_-300 than CeO_2_. The long-term transient photocurrent response of the two catalysts further confirms the stronger oxidation capability of Au-CeO_2_-300 compared to CeO_2_ (Fig. 4b).

To obtain an in-depth understanding of the photocatalytic methane oxidation process over CeO_2_ and Au-CeO_2_-300, in situ diffuse reflectance infrared Fourier transform spectroscopy (DRIFTS) was carried out. The DRIFTS spectra of the catalysts in dark in the reaction atmosphere (Supplementary Fig. 55) were used as the baseline when performing the measurement under light irradiation. As shown in Supplementary Fig. 56, the peak at 2885/2843 cm^-1^ is associated with the C-H stretching vibration of the adsorbed CH_3_· species^14,19,20,56^, which is the first intermediate generated from methane activation by photoholes. Two peaks at 1402 and 1566 cm^-1^, and three peaks at 1373, 1433 and 1548 cm^-1^ are observed on the spectra of CeO_2_ and Au-CeO_2_-300, respectively. These peaks are attributed to HCOO· species, which is another important intermediate during the overoxidation process^55^. A carbonate species is detected at 1263/1236 cm^-1^ (Supplementary Figs. 56b and 57)^19^. The overoxidation product CO_2_ is observed at 2360 cm^-1^ for both photocatalysts. The IR band positions of all detected species are summarised in Supplementary Table 5. A red shift in the bands of all adsorbed species (e.g., CH_3_·, CO_3_^2-^· and HCOO·) is observed in the spectrum of Au-CeO_2_-300 compared with CeO_2_, indicating different adsorption sites of the intermediates on the surface of the two catalysts. As discussed in the above charge separation section, Au acts as a hole acceptor in Au-CeO_2_-300 and methane is activated by holes transferred from CeO_2_ to Au. Thus, the intermediates formed in the subsequent oxidation steps are possibly adsorbed on Au or at the interface of Au and CeO_2_.

The quantity of survived species observed by DRIFTS is plotted as a function of reaction time to provide insights into the evolution and transformation of intermediates during photocatalytic methane oxidation (Fig. 4c, d and Supplementary Fig. 58). The amount of CH_3_· first increases and then fluctuates at a certain concentration for both CeO_2_ and Au-CeO_2_-300 (Supplementary Fig. 58). However, the peak intensity of Au-CeO_2_-300 is much stronger than that of CeO_2_, indicating the superior methane activation capability of Au-CeO_2_-300. The formed CH_3_**·** follows two routes: (1) two CH_3_**·** couple into C_2_H_6_, and (2) CH_3_**·** is overoxidised, leading to the production of CO_2_. Gas-phase CH_3_**·** species has been observed using EPR and photoionisation mass spectroscopy^57,58^. Thus, the coupling of CH_3_**·** could occur in the gas phase or on the catalyst surface. In the case of gas-phase radical reaction, Au can facilitate the desorption of CH_3_· from the catalyst surface due to the lowest adsorption energy based on the adsorption energies of CH_3_· calculated on the surface of CeO_2_ and Au-CeO_2_-300 (Supplementary Table 6). This suggests that CH_3_**·** radicals on Au can readily desorb and couple into C_2_H_6_, while the CH_3_**·** on CeO_2_ likely undergoes overoxidation by photoholes or surface O_2_^-^ species since it is strongly bonded with O of CeO_2_. The other case, where the radical reaction occurs on the surface of the catalyst, was also investigated by DFT simulation. The results indicate that the energy barrier for CH_3_**·** coupling on the Au surface is much lower than that on the CeO_2_ surface (Supplementary Fig. 59). This could also be revealed by the strong CH_3_O· peaks observed in the DRIFTS spectrum of CeO_2_ (Supplementary Fig. 51). Then, the strongest peak related to HCOO· is analysed (Fig. 4c). The peak area of this band keeps increasing with time over Au-CeO_2_-300, indicating the accumulation of HCOO· on the surface of the catalyst. It suggests the consumption of HCOO· is much slower than its formation, and HCOO· oxidation is the rate-limiting step in methane oxidation to CO_2_ over Au-CeO_2_-300. In contrast, this band is not detected when CeO_2_ is used as the catalyst, and the intensity of the other two bands at 1402 and 1566 cm^-1^ related to HCOO· is also weak. This implies that the HCOO· radicals formed on CeO_2_ can be facilely converted to CO_2_, the final overoxidation product. Thus, a very low amount of HCOO· is observed on the surface of CeO_2_. This is also evidenced by the instant evolution of gaseous CO_2_ over CeO_2_ under photocatalytic reaction conditions (Fig. 4d), while only a weak signal of CO_2_ is observed after reaction for 20 min when Au-CeO_2_-300 is used as the catalyst. The DRIFTS analysis reveals that the loading of Au on CeO_2_ could efficiently suppress the overoxidation reaction in photocatalytic methane oxidation by restricting the transformation of HCOO· radicals. To confirm this, the photocatalytic oxidation of HCOONa over CeO_2_, Au-CeO_2_-300, and Pt-CeO_2_ was measured (Supplementary Fig. 60). The CO_2_ production rate over CeO_2_, Au-CeO_2_-300, and Pt-CeO_2_ are 457, 43, and 843 μmol h^-1^, respectively. The results reveal that the oxidation of HCOO**·** to CO_2_ is restricted by loading Au onto CeO_2_. However, the CO_2_ production rate nearly doubles by loading of Pt, which is an electron acceptor. Since Au loading blocks the CH_3_**·** overoxidation pathway, more CH_3_**·** species follow the coupling pathway to form C_2+_ hydrocarbons.

**Fig. 5:**
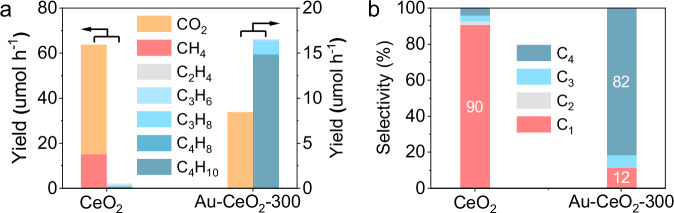
Ethane oxidation reaction. **a** Yield and (**b**) selectivity for ethane oxidation to different products over CeO_2_ and Au-CeO_2_-300. Reaction conditions: 50 mg catalyst, ethane to air = 5:1, GHSV = 120 000 mL h^−1^ g^−1^, Pressure = 1 bar, Temperature = 50 °C, 365 nm LED, light intensity = 100 mW cm^-2^.

To understand the origin of the high selectivity of Au-CeO_2_-300 towards C_2+_ hydrocarbons, ethane, the main product in methane oxidation was introduced as a reactant into the photocatalytic oxidation reaction over CeO_2_ and Au-CeO_2_-300 (Fig. 5). When CeO_2_ is used as the photocatalyst, the main product is CO_2_ at a yield of 49 μmol h^-1^ (Fig. 5a). Apart from CO_2_, CH_4_ at a production rate of 15 μmol h^-1^ is detected. Only a trace amount of C_2+_ molecules (e.g., C_2_H_4_, C_3_H_8_, C_3_H_6_, C_4_H_10_ and C_4_H_8_) are observed in the product. In contrast, when Au-CeO_2_-300 is used for ethane oxidation under identical conditions, the CO_2_ product rate is only 5 μmol h^-1^ (10 times lower than that over CeO_2_), and CH_4_ is not detectable in the products. The major product is switched to C_4_H_10_ at a high yield of 15 μmol h^-1^. To provide a clear product distribution for ethane oxidation over the photocatalysts, the selectivity of products is calculated based on the number of carbon in the molecules (Fig. 5b). It clearly shows that C_1_ molecules with a high selectivity of 90% are the main products of photocatalytic ethane oxidation by CeO_2_. In contrast, C_4_ hydrocarbons account for 82% of the products when Au-CeO_2_-300 is applied. To produce C_1_ molecules, the C-C bond in ethane has to be broken. To form a C_4_ molecule, a new C-C bond should be formed to connect two C_2_H_6_ molecules. Thus, the CeO_2_ surface is prone to catalyse the C-C bond-breaking reaction. It further indicates that C_2_H_6_ could be overoxidised to CO_2_ or transferred back to CH_4_ even if it is formed by the coupling of CH_3_· in photocatalytic methane oxidation over CeO_2_. On the contrary, Au-CeO_2_-300 can promote the C-C coupling reaction under photocatalytic conditions. Therefore, CH_3_· radicals are more likely to couple into C_2_H_6_ on the Au-CeO_2_-300 surfaces. This also explains the formation of C_3_ and C_4_ products by Au-CeO_2_-300 in methane oxidation. It is worth noting that the ethane conversion rates over CeO_2_ and Au-CeO_2_-300 are 35 and 36 μmol h^-1^, respectively, indicating that Au loading on CeO_2_ has little effect on the conversion of ethane. Overall, these results provide strong evidence that Au on the surface of CeO_2_ can promote the C-C bond coupling reaction and avoid overoxidation, thus boosting the selectivity towards C_2+_ molecules in photocatalytic methane oxidation.

**Fig. 6:**
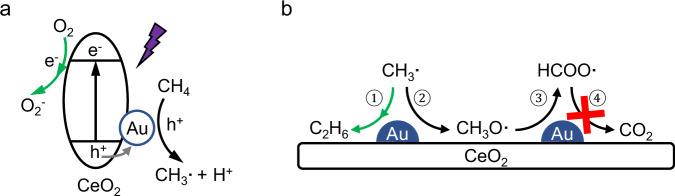
Proposed methane oxidation process. **a** Change separation-migration and reactant activation, and (**b**) the effect of Au on promoting C-C coupling, and suppressing overoxidation over Au-CeO_2_−300.

Based on the analysis above, a reaction mechanism for photocatalytic methane oxidation over Au-CeO_2_-300 is proposed (Fig. 6). When the photocatalyst is irradiated by the 365 nm LED, electrons are populated to the CB of CeO_2_, leaving holes on the VB of CeO_2_. The photoelectrons reduce oxygen gas to produce superoxide radicals. The existence of O_V_s in CeO_2_ improves the oxygen adsorption and reduction processes. Au acts as a hole acceptor and enhances charge separation efficiency. The photoholes at Au transferred from VB of CeO_2_, then activate methane molecules to generate methyl radicals and protons (Fig. 6a). The protons are consumed by superoxide radicals, resulting in the formation of water. The coupling reaction of methyl radicals leads to the formation of ethane (step ① in Fig. 6b), which is the main pathway for the consumption of methyl radicals. This is due to the existence of Au nanoparticles, which boost the desorption and coupling of CH_3_· radicals. A small portion of methyl radicals undergoes overoxidation to CH_3_O· and further to HCOO· (steps ② and ③ in Fig. 6b). On pristine CeO_2_ surface, CH_3_· is strongly bonded on the catalyst surface, which leads to severe overoxidation. Moreover, HCOO· is readily converted to CO_2_, which further promotes overoxidation. Thus, a low selectivity towards C_2+_ products is observed over CeO_2_. In contrast, the presence of Au co-catalyst considerably reduces the efficiency of HCOO· oxidation (step ④ in Fig. 6), which limits the overoxidation process. Therefore, both a high yield and selectivity of C_2+_ hydrocarbons are achieved in photocatalytic methane oxidation over Au-CeO_2_-300.

In summary, this study reported the efficient and selective oxidative coupling of methane using Au-loaded CeO_2_ by photon-phonon co-driven catalysis. The optimised Au-CeO_2_-300 achieves a C_2_H_6_ yield of 755 μmol h^-1^ (15,100 μmol g^-1^h^-1^) and a C_2+_ selectivity of 98%, with the excellent stability of at least 120 h. The introduction of oxygen vacancies into Au-CeO_2_ by pre-treatment at 300 °C further promotes the adsorption and activation of oxygen gas. Charge migration studies confirm that Au acts as a hole acceptor and improves charge separation in photocatalysis. Surface chemistry analysis shows that Au as a co-catalyst on CeO_2_ can accelerate both the oxygen reduction and the methane oxidation half-reactions. In situ DRIFTS characterisation reveals that Au suppresses overoxidation by restraining the conversion of HCOO· radicals. Finally, the control experiment of the ethane oxidation reaction indicates that Au can promote the C-C coupling reaction and mitigate overoxidation, thereby boosting the selectivity towards C_2+_ products during photocatalytic methane oxidation. These findings provide important insights into the catalyst design and fundamental understandings of catalytic partial oxidation of methane. With further light source engineering, reactor design and reaction optimisation, this study can offer a promising and potential solution for economical and sustainable methane conversion to high-value hydrocarbons under mild conditions.

## Methods

### Materials synthesis

Au was loaded on CeO_2_ by a chemical reduction method. 50 mg CeO_2_ (25 nm nanopowder, Sigma-Aldrich) was dispersed in 50 mL deionised water and stirred for 30 min. Then, a certain amount of HAuCl_4_·4H_2_O (5 mg (Au) mL^-1^, Sigma-Aldrich) solution was added and stirred for another 30 min. Subsequently, 5 mL NaBH_4_ (2 mg mL^-1^, Sigma-Aldrich) solution was added drop by drop in the above suspension. After stirring for 60 min, the product was washed by centrifugation and dried at 60 °C. To further improve the activity of the photocatalysts, Au-loaded CeO_2_ was pretreated in a muffle furnace at various temperatures from 200 to 500 °C for 2 h.

### Characterisations

X-ray diffraction (XRD) was carried out with two Stoe STADI-P diffractometers, one equipped with a Mo Kα source (λ = 0.7073 Å, scanned from 3 to 35°) and another equipped with a Cu Kα source (λ = 1.5418 Å, scanned from 10 to 85°). Ultraviolet-visible diffuse reflectance spectroscopy (UV-Vis DRS) was measured by a Shimadzu UV-2550 spectrophotometer fitted with an integrating sphere. The reflectance values were directly converted to absorption by the Kubelka-Munk equation via the UV-Probe 2.33 software. X-ray photoelectron spectroscopy (XPS) was measured by a Thermo Scientific XPS instrument equipped with an Al Kα source (hν = 1486.6 eV). The in situ XPS measurement in dark and under light irradiation conditions was performed using the same sample. The XPS spectrum of the sample in dark was firstly measured. Then, another spectrum was acquired after the sample was irradiated by a 365 nm LED light for 10 min. Both spectra were calibrated using the C 1 s spectra obtained in dark and light irradiation, respectively. The Au content in the photocatalysts was measured by inductively coupled plasma-atomic emission spectrometry (Agilent, ICP-OES 5800). Raman spectra were measured by a Renishaw InVia multi-channel Raman spectroscopy with a 514 nm excitation laser. Transmission electron microscopy (TEM) was carried out on a JEOL 2010 instrument. The size distribution of Au is obtained by measuring the diameter of >100 nanoparticles for each sample. Photoluminescence (PL) was collected from 330 nm to 800 nm by a Renishaw InVia spectroscopy with a 325 nm laser as the excitation source.

### Photocatalytic reaction test

The photocatalytic oxidative coupling of methane was carried out in a pressurised flow reaction system. The reactor used is a stainless steel cell equipped with a quartz window. All parts of the reaction system are connected by stainless steel tubings. The flow rates of methane, air and Ar were controlled by three mass flow controllers (Bronkhorst). Two pressure gauges were fitted before and after the reactor to monitor the pressure change during the photocatalytic reaction. No pressure drop was observed after the reaction. A regulator valve at the end of the reaction system was used to adjust and maintain the pressure in the reactor. A heating unit at the bottom of the catalyst bed is used to control the reaction temperature from room temperature to 200 °C. The photocatalyst was filtered onto a glass fibre membrane and fitted into the cell for the performance test. To prepare the membrane, 50 mg photocatalyst was dispersed in 200 mL deionised water and stirred for 30 min. Then, the suspension was filtered by the membrane and dried at 60 °C for 12 h. The membrane was fixed in the reactor by a stainless steel ring. The top and bottom parts of the reactor were sealed with a rubber ring and a stainless steel clamp. The light source used was a 365 nm LED (100 W, Beijing Perfect Light), a Xe lamp (300 W, Beijing Perfect Light) equipped with a 420 nm long-pass filter, and a 450 nm LED (100 W, Beijing Perfect Light). The light intensity of the 365 nm LED could be adjusted from 10 to 200 mW/cm^2^, depending on the reaction conditions. The surface temperature of the catalyst was measured by a radiative thermometer (Extech Instruments, IR320). The product released from the regulator valve was directly connected to a GC (Varian 450) equipped with a TCD detector, a methanizer, and an FID detector. The isotopic labelling tests were performed in a quartz batch reactor (100 mL). The isotopic labelled ^13^CH_4_, CD_4_ and ^18^O_2_ are purchased from Sigma-Aldrich. After purging the reactor with N_2_ (BOC) for 30 min, the feedstock (CH_4_ + O_2_, ^13^CH_4_ + O_2_, CD_4_ + O_2_, or CH_4_ + ^18^O_2_) with CH_4_ to O_2_ ratio of 10:1 was added into the reactor and irradiated by a 365 nm LED (100 W) for 15 min. The gas products were analysed by GC-MS (Shimadzu GCMS-QP2020 NX).

Methane conversion was calculated by:

2 $${{Con}.}_{{{CH}}_{4}}=\frac{{nmuber\; of\; conversted} \, {{CH}}_{4}}{{number\; of\; input} \, {{CH}}_{4}}\times 100\%$$

Oxygen conversion was calculated by:

3 $${{Con}.}_{{O}_{2}}=\frac{{nmuber\; of\; conversted} \, {O}_{2}}{{number\; of\; input} \, {O}_{2}}\times 100\%$$

The selectivities are calculated based on observable products as the following:

4 $$	{Selectivity\; of} {C}_{2+} \\ 	=\frac{2\times {n}_{{{C}_{2}H}_{6}}+2\times {n}_{{{C}_{2}H}_{4}}+3\times {n}_{{{C}_{3}H}_{8}}+3\times {n}_{{{C}_{3}H}_{6}+}4\times {n}_{{{C}_{4}H}_{10}}}{2\times {n}_{{{C}_{2}H}_{6}}+2\times {n}_{{{C}_{2}H}_{4}}+3\times {n}_{{{C}_{3}H}_{8}}+3\times {n}_{{{C}_{3}H}_{6}+}4\times {n}_{{{C}_{4}H}_{10}}+{n}_{{{CO}}_{2}}}\times 100\%$$

5 $$	{Selectivity\; of}{{CO}}_{2} \\ 	=\frac{{n}_{{{CO}}_{2}}}{2\times {n}_{{{C}_{2}H}_{6}}+2\times {n}_{{{C}_{2}H}_{4}}+3\times {n}_{{{C}_{3}H}_{8}}+3\times {n}_{{{C}_{3}H}_{6}+}4\times {n}_{{{C}_{4}H}_{10}}+{n}_{{{CO}}_{2}}}\times 100\%$$

Carbon balance was calculated by:

6 $${{{\rm{Carbon\; balance}}}}=\frac{{number\; of}C \, {in\; products}}{{number\; of\; converted} \, {{CH}}_{4}}\times 100\%$$

Oxygen balance was calculated by:

7 $$	{{{\rm{Oxygen\; balance}}}} \\ 	=\frac{\frac{1}{2}n{C}_{2}{H}_{6}+n{C}_{2}{H}_{4}+n{C}_{3}{H}_{8}+\frac{3}{2}n{C}_{3}{H}_{6}+\frac{3}{2}n{C}_{4}{H}_{10}+n{{CO}}_{2}}{n \, {of\; converted} \, {O}_{2}}\times 100\%$$

The apparent quantum efficiency (AQE) was calculated based on the conversion of methane:

8 $$\begin{array}{c}{AQE}=\frac{{Number\; of\; electrons\; transferred}}{{Number\; of\; incident\; photons}}\times 100\%\\=\frac{\left(2\times {n}_{{{C}_{2}H}_{6}}+4\times {n}_{{{C}_{2}H}_{4}}+4\times {n}_{{{C}_{3}H}_{8}}+6\times {n}_{{{C}_{3}H}_{6}+}6\times {n}_{{{C}_{4}H}_{10}}+{8\times n}_{{{CO}}_{2}}\right)\times {N}_{A}}{\frac{I\times A}{{E}_{g}\times J}}\times 100\%\end{array}$$

Where N_A_ is Avogadro’s constant 6.02 × 10^23^, I is the light intensity in the unit of mW cm^-2^, A is the irradiation area 7 cm^2^, E_g_ is the energy of a photon with 365 nm wavelength and J is the amount of charge in one electron and used to transform the unit of photon energy from eV to J.

### Supplementary information

Supplementary Information

Peer Review File

### Source data

Source Data

## Data Availability

Data supporting this study are available in the Supplementary information and in the Source Data file provided with this manuscript. Source data are provided with this paper.
