# Supplementary material for: Selective oxidation of methane to C2+ products over Au-CeO2 by photon-phonon co-driven catalysis
Source: Nat Commun. 2024 Aug 30;15:7535. doi: 10.1038/s41467-024-51690-2 (PMC11364766; doi:10.1038/s41467-024-51690-2)
Supplement: Supplementary file 1 — Supplementary Information [file 41467_2024_51690_MOESM1_ESM.pdf]

Supplementary Information for

Selective oxidation of methane to C_{2+} products over Au-CeO_2 by photon-phonon co-driven catalysis

Chao Wang[1], Youxun Xu[1], Lunqiao Xiong[1,2], Xiyi Li[1], Enqi Chen[1], Tina Jingyan Miao[1], Tianyu Zhang[3*], Yang Lan[1], and Junwang Tang[1,2*]

[1] Department of Chemical Engineering, University College London, London WC1E 7JE, United Kingdom

[2] Industrial Catalysis Center, Department of Chemical Engineering, Tsinghua University, Beijing 100084, China

[3] Beijing Key Lab for Source Control Technology of Water Pollution, College of Environmental Science and Engineering, Beijing Forestry University, Beijing, 100083 P. R. China

[*]Corresponding author: Dr Tianyu Zhang. Email: tzhang@bjfu.edu.cn; Prof. Junwang Tang. Email: jwtang@tsinghua.edu.cn

Supplementary Methods

Synthesis of catalysts

Synthesis of M-CeO_2 (M=Pt, Ru, Pd, Rh)

M-CeO_2 (M=Pt, Ru, Pd, Rh) was synthesised by a chemical reduction method using $H_2PtCl_6·6H_2O$, $RuCl_3$, $PdCl_2$, and $RhCl_3$ (Sigma-Aldrich) as the precursor for Pt, Ru, Pd, and Rh, respectively. In a typical synthesis, 50 mg CeO_2 (25 nm nanopowder, Sigma-Aldrich) was dispersed in 50 mL deionised water and stirred for 30 min. Then, 100 µL of the metal precursor (5 mg (Au) mL^{-1} based on the corresponding metal) solution was added and stirred for another 30 min. Subsequently, 5 mL $NaBH_4$ solution (2 mg mL^{-1}, Sigma-Aldrich) was added drop by drop in the above suspension. After stirring for 60 min, the product was washed by centrifugation and dried at 60 °C. The product was denoted M-CeO_2.

Synthesis of MnO_x-CeO_2

MnO_x-CeO_2 was synthesised by a photo-deposition method. 50 mg CeO_2 (25 nm nanopowder, Sigma-Aldrich) was dispersed in 50 mL $NaIO_3$ (0.02 M) solution and stirred for 30 min. Then, 100 µL of $MnSO_4$ (5 mg (Mn) mL^{-1}, Sigma-Aldrich) solution was added and stirred for another 30 min. The suspension was transferred to a quartz cell and purged with Ar (BOC) for 30 min to remove air. Subsequently, the suspension was irradiated by a 300 W Xe lamp for 2 h under continuous stirring. After the reaction, the product was washed by centrifugation and dried at 60 °C. The product was denoted MnO_x-CeO_2.

Synthesis of CoO_x-CeO_2 and CuO_x-CeO_2

CoO_x-CeO_2 and CuO_x-CeO_2 were synthesised by an impregnation method using $Co(NO_3)_2·6H_2O$ (Sigma-Aldrich) and $Cu(NO_3)_2·6H_2O$ (Sigma-Aldrich) as the precursor for Co and Cu, respectively. Typically, 50 mg CeO_2 (25 nm nanopowder, Sigma-Aldrich) was dispersed in 10 mL deionised water and stirred for 30 min. Then, 100 µL of $Co(NO_3)_2·6H_2O$ or $Cu(NO_3)_2·6H_2O$ (5 mg (Co or Cu) mL^{-1}) solution was added into the above solution and stirred at 80 °C in a fume hood until dry. The mixture was then calcined in a muffle furnace at 300 °C for 2 h. The product was denoted CoO_x-CeO_2 or CuO_x-CeO_2.

Synthesis of CeO_2-300 and CeO_2-500

CeO_2-300 and CeO_2-500 were synthesised by calcination of CeO_2 in a muffle furnace at 300 and 500 °C, respectively.

Synthesis of Au-CeO_2-Na

Au-CeO_2-Na was synthesised by a modified $NaBH_4$ reduction method. 50 mg CeO_2 (25 nm nanopowder, Sigma-Aldrich) was dispersed in 50 mL deionised water and stirred for 30 min. Then, 100 µL of $HAuCl_4·4H_2O$ (5 mg (Au) mL^{-1} based on metallic Au, Sigma-Aldrich) solution was added and stirred for another 30 min. Subsequently, 5 mL $NaBH_4$ solution (2 mg mL^{-1}, containing 0.5 M NaOH, Sigma-Aldrich) was added drop by drop in the above suspension. After stirring for 60 min, the product was washed by centrifugation and dried at 60 °C. The product is denoted Au-CeO_2-Na.

Synthesis of Au-CeO_2-Na-300

Au-CeO_2-Na-300 was prepared by calcination of Au-CeO_2-Na in a muffle furnace at 300 °C for 2 h.

Oxygen adsorption measurement

The oxygen adsorption property of CeO_2, CeO_2-300, and CeO_2-500 was measured using a stainless-steel reactor in dark. In a typical test, 200 mg sample powder was loaded into the reactor. Then, the sample was treated in an Ar flow with a flow rate of 50 mL min^{-1} at 150 °C for 1 h to remove the surface adsorbed species. Afterwards, an Ar flow containing 40 ppm O_2 at a flow rate of 20 mL min^{-1} was introduced. The oxygen concentration in the outlet gas after various running times was analysed by a GC equipped with a TCD detector. The change in the oxygen concentration compared with that measured in the absence of a catalyst is caused by the adsorption of oxygen on the sample surface.

In situ ultraviolet-visible diffuse reflectance spectroscopy (UV-Vis DRS) measurement

An Agilent Cary 5000 UV-Vis-NIR spectrometer was used for the in situ charge behaviour measurement. A Praying Mantis accessory equipped with a Harrick cell was fitted into the Cary 5000 spectrometer to align the beam path and control the sample environment. The three windows of the Harrick cell are made from crystal quartz and transparent for UV and visible light. Two windows are for the transmission of measurement light, and the other is for the excitation light (Supplementary Fig. 31). A UV LED (365 nm, 12 W) fitted with a 365 nm band pass filter was used as the irradiation source. The 365 nm bandpass filter was employed to ensure a clean 365 nm irradiation. Another 450 nm long pass filter (λ > 450 nm) was fitted before the detector of the spectrometer to avoid the interference of the scattering light from the LED source. In each measurement, 100 mg catalyst was loaded into the sample holder of the Harrick cell. A metal spatchaler was used to create a flat surface of the sample for measurement. Then, the Harrick cell was purged by Ar, air or methane for 30 min at a flow rate of 50 mL/min. Afterwards, the cell was sealed for measurement. The reflectance of each sample was measured from 500 to 800 nm both in dark and under LED irradiation. Each measurement was performed three times and the average reflectance values were used for analysis.

Electrochemical measurement

The working electrode was prepared by a spin-coating method. 10 mg photocatalyst was dispersed in 1 mL ethanol and sonicated for 30 min. Then 80 µL of the suspension was dropped on a piece of fluorine-doped tin Oxide (FTO) glass. The FTO was fixed on the spin coater for 30 s at 300 rpm. Finally, the electrode was dried on an 80 °C hot plate. All the electrochemical tests were performed in a three-electrode cell with 0.5 M $NaSO_4$ (Sigma-Aldrich) solution as the electrolyte. The FTO coated with the photocatalyst, a Pt plate, and a Ag/AgCl in 3 M KCl electrode were used as the working electrode, counter electrode, and reference electrode, respectively. The linear sweep voltammetry (LSV) was performed at a voltage window from 0 to 0.6 V for the water oxidation reaction and from 0 to -1.2 V for the oxygen reduction reaction at a scan rate of 0.01 V s^{-1} to measure the oxidation capability of the catalysts. During the test, the lamp was chopped on and off automatically at an interval of 5 s. The light source used was a 150 W Xe lamp. For the photocurrent measurement, a bias potential of 0.3 V was applied. Similarly, an on-and-off interval of 10 s was applied over a period of 90 s. For the open circuit photovoltage decay (OCVD) test, the photoelectrochemical cell was first kept in dark for 10 min

to ensure an equilibrium potential was obtained. Then, the open circuit voltage was recorded. The working electrode was illuminated by a 150 W Xe lamp at Time = 50 s, after which a photovoltage was generated. The generation of photovoltage results from the accumulation of charge carriers in the photocatalysts. A negative photovoltage is obtained, as the major carriers are electrons in CeO_2. After irradiation for another 100 s, the light was turned off and fast decay of the photovoltage was observed.

Superoxide radical trapping experiment

Superoxide (O_2^-) radicals were trapped by 5,5-dimethyl-1-pyrroline N-oxide (DMPO, Sigma-Aldrich) as the spin-trapping reagent and monitored through electron paramagnetic resonance (EPR) in a Bruker A200 spectrometre. [1,2] Typically, 5 mg photocatalyst was dispersed in 5 mL methanol (Sigma-Aldrich) solution containing 25 mM DMPO. After stirring for 3 min, the suspension was loaded into a capillary tube. The capillary tube was quickly sealed and put into a quartz tube for measurement. The EPR measurement was conducted at room temperature, with a modulation frequency of 100 kHz, a microwave frequency of 9.32 GHz, a sweep time of 80 s, a microwave power of 5.04 mW, and swept from 3320 to 3270 G. When conducting the experiment under light conditions, the suspension was irradiated with a 365 nm LED source for 30 s before loading into the capillary tube. In the measurement, methanol i) plays a similar role as methane in the oxidation reaction (reacting with photoholes) and ii) works as a solvent to disperse the radical trapping reagent. In other words, the overall reaction is methanol oxidation by photoholes, and oxygen reduction by photoelelctrons, which is similar to the methane oxidation reaction (methane oxidation by photoholes, and oxygen reduction by photoelelctrons).

In situ diffuse reflectance infrared Fourier transform spectroscopy (DRIFTS) measurement

In situ DRIFTS was measured with a Shimadzu IRTracer-100 Fourier transform infrared spectrometre equipped with a Harrick cell and a Pray Mantis accessory. Two ZnSe windows and one quartz window were used on the cell for IR transmittance and UV irradiation, respectively. In each test, 100 mg catalyst was loaded in the sample holder of the cell. A metal spatchaler was used to create a flat surface of the sample for measurement. Then, the cell was purged with methane and air at flow rates of 400 and 2 mL min^{-1}, respectively for 30 min in dark. Afterwards, the cell was sealed and fixed in the Praying Mantis accessory. The Praying Mantis accessory with the Harrick cell was finally fixed in the FTIR spectrometre for measurement. The signal of the photocatalyst in the reaction atmosphere in dark was used as the baseline. A 12 W 365 nm LED or a 100 W 365 nm LED was used to irradiate the catalysts during measurement. The IR spectra were obtained every 10 min for 60 min for each catalyst.

Photocatalytic ethane oxidation reaction

The photocatalytic oxidation of ethane was tested using the same setup as photocatalytic OCM. The flow rates of ethan, air and Ar (all from BOC) were 10, 2 and 88 mL min^{-1}, respectively, corresponding to a GHSV of 120 000 mL h^{-1} g^{-1}. The reaction temperature and pressure were 50 °C and 1 bar unless specifically stated otherwise. The light intensity applied was 100 mW cm^{-2}.

Photocatalytic HCOONa oxidation reaction

The photocatalytic HCOONa oxidation reaction was performed in a quartz reactor (50 mL). 20 mg of catalyst was dispersed in 20 mL HCOONa (5 mg mL^{-1}, Sigma-Aldrich) solution. The mixture was then purged with synthetic air (20% O_2 and 80% N_2, BOC) for 30 min in dark. The reactor was then irradiated by a 100 W 365 nm LED with a light intensity of 200 mW cm^{-2} under stirring for 30 min. The gas product was sampled by a syringe (1 mL) and analysed by a GC.

Computational details

All DFT calculations were performed using periodic DFT methods implemented in the Vienna ab initio simulation package (VASP6.1).[3,4] Projector augmented wave (PAW) method was used for the interaction between the atomic cores and valence electrons and the exchange-correlation energies were calculated via the generalised gradient approximation (GGA) with the PBE functional.[5,6] To partially correct the strong electron-correlation properties of these oxides, DFT+U calculations[5,6] were performed with U= 5 eV, with the U values taken from the literature.[7,8] An energy cutoff of 400 eV was used for the plane-wave basis set. The geometries were optimized with the self-consistent field and force convergence criteria set to 10^{-5} eV and 0.03 eV/Å, respectively. The charge density differences were evaluated using the formula Δρ = ρA+B – ρA – ρB, where ρX is the electron density of X. A 4x4 supercell of CeO_2 111 surface with three CeO_2 sublayer (each consists of one Ce atomic layer and two oxygen atomic layers) was selected, and the vacuum spacing between two repeated slabs was set to 15 Å. K-points grid of 1x1x1 was used to sample the Brillouin zones. For geometry optimization, the bottom sublayer of CeO_2 was fixed. The transition states were acquired using the climbing image nudged elastic band (CI-NEB) method[9–11] by relaxing the force below 0.05eV/Å, which was further confirmed by the analysis of the only one imaginary frequency.

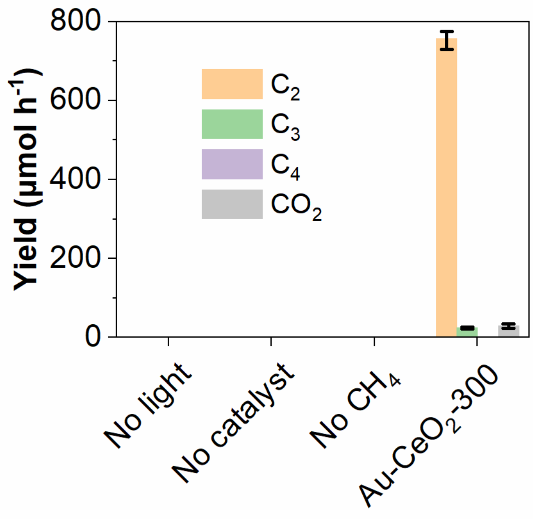

Supplementary Fig. 1 Product yield in control experiments. Results of control experiments with no light, no catalyst, no methane, and standard photocatalytic reaction conditions over Au-CeO_2-300. Error bars represent standard deviations calculated from the performance tests of the photocatalysts prepared in three different batches. Reaction conditions: 50 mg catalyst, methane to air = 200:1, GHSV = 480 000 mL h^{-1} g^{-1}, Pressure = 5 bar, Temperature = 150 °C, 365 nm LED, light intensity = 200 mW cm^{-2}.

A one-pass methane conversion of 0.14% and oxygen conversion of 45% are achieved. The carbon and oxygen balance are calculated to be 92.5% and 90.8%, respectively. Although a methane to air ratio of 200:1 was used, there is sufficient oxygen gas in the reaction system for reaction.

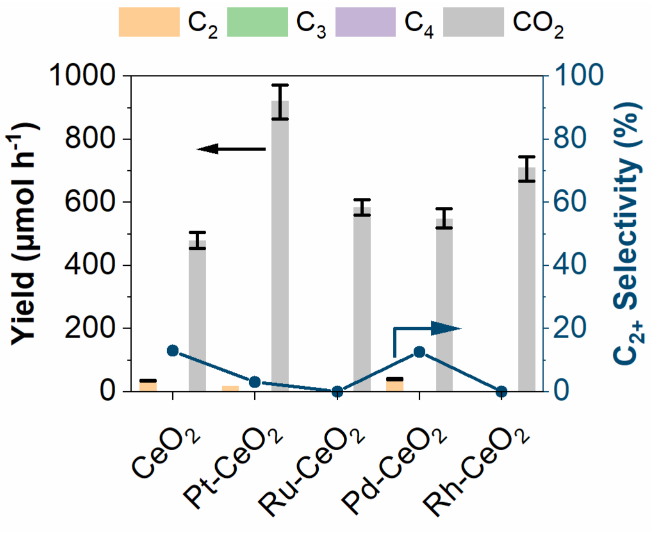

Supplementary Fig. 2 Effects of reduction co-catalysts. Photocatalytic methane conversion performance of CeO_2, Pt-CeO_2, Ru-CeO_2, Pd-CeO_2, and Rh-CeO_2. Error bars represent standard deviations calculated from the performance tests of the photocatalysts prepared in three different batches. Reaction conditions: 50 mg catalyst, methane to air = 200:1, GHSV = 480 000 mL h^{-1} g^{-1}, Pressure = 5 bar, Temperature = 150 °C, 365 nm LED, light intensity = 200 mW cm^{-2}.

Pt, Ru, Pd, and Rh were loaded onto CeO_2 with a similar $NaBH_4$ reduction method to Au-CeO_2 using different precursors of the corresponding metal. The results indicate that loading these metals as co-catalysts on CeO_2 has dramatically improved CH_4 oxidation to CO_2. For instance, the CO_2 production rate increases from 478 to 920 μmol h^{-1} after loading Pt onto CeO_2. The production of C_{2+} is not promoted by these co-catalysts as the C_{2+} selectivity is lower than 20% among all photocatalysts. This is because the electron-accepting co-catalyst plays a key role in promoting the reduction half-reaction to form excessive O_2^-. However, the oxidation half-reaction is not modified. The overall effect of electron-accepting co-catalysts is to improve methane oxidation to CO_2.

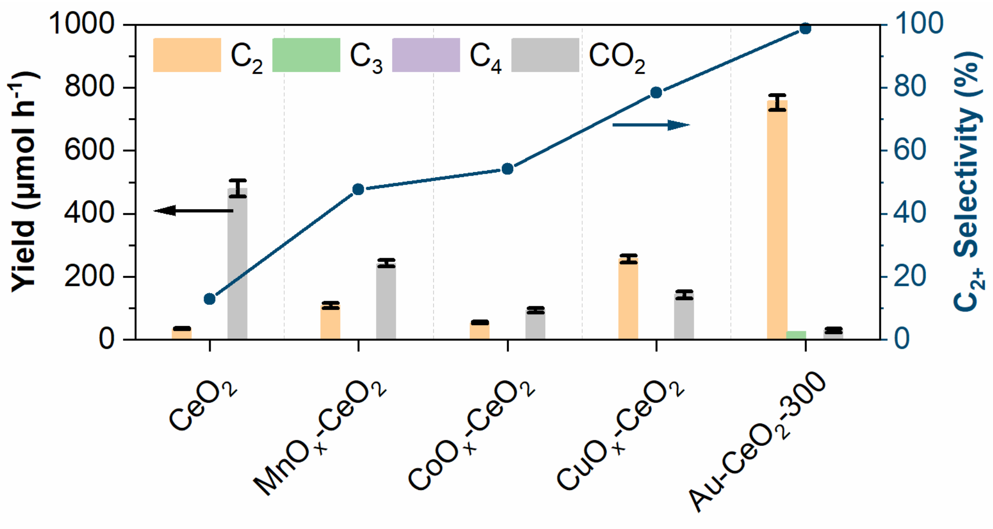

Supplementary Fig. 3 Effects of oxidation co-catalysts. Photocatalytic methane conversion performance of CeO_2, MnO_x-CeO_2, CoO_x-CeO_2, CuO_x-CeO_2, and Au-CeO_2-300. Error bars represent standard deviations calculated from the performance tests of the photocatalysts prepared in three different batches. Reaction conditions: 50 mg catalyst, methane to air = 200:1, GHSV = 480 000 mL h^{-1} g^{-1}, Pressure = 5 bar, Temperature = 150 °C, 365 nm LED, light intensity = 200 mW cm^{-2}.

Oxides of Co, Mn, and Cu have been reported to act as hole-accepting co-catalysts in photocatalysis.[12–14] Therefore, MnO_x was loaded onto CeO_2 by a photo-deposition method and CoO_x and CuO_x were loaded onto CeO_2 by an impregnation method. The catalysts are denoted MnO_x-CeO_2, CoO_x-CeO_2, and CuO_x-CeO_2, respectively. After modification of CeO_2 with the hole-accepting co-catalysts, photo-generated roles transfer from CeO_2 to the metal oxides, resulting in reduced oxidation potential of holes, hence a decrease in the yield of overoxidation product, CO_2. The yield of C_2H_6 is improved to different levels over the various co-catalysts used. The selectivity towards C_{2+} hydrocarbon is improved from 13% over CeO_2 to 48% over MnO_x-CeO_2, 54% over CoO_x-CeO_2, and 78% over CuO_x-CeO_2. Au-CeO_2-300 displays the highest C_2H_6 yield of 755 μmol h^{-1} and C_{2+} selectivity of 98%.

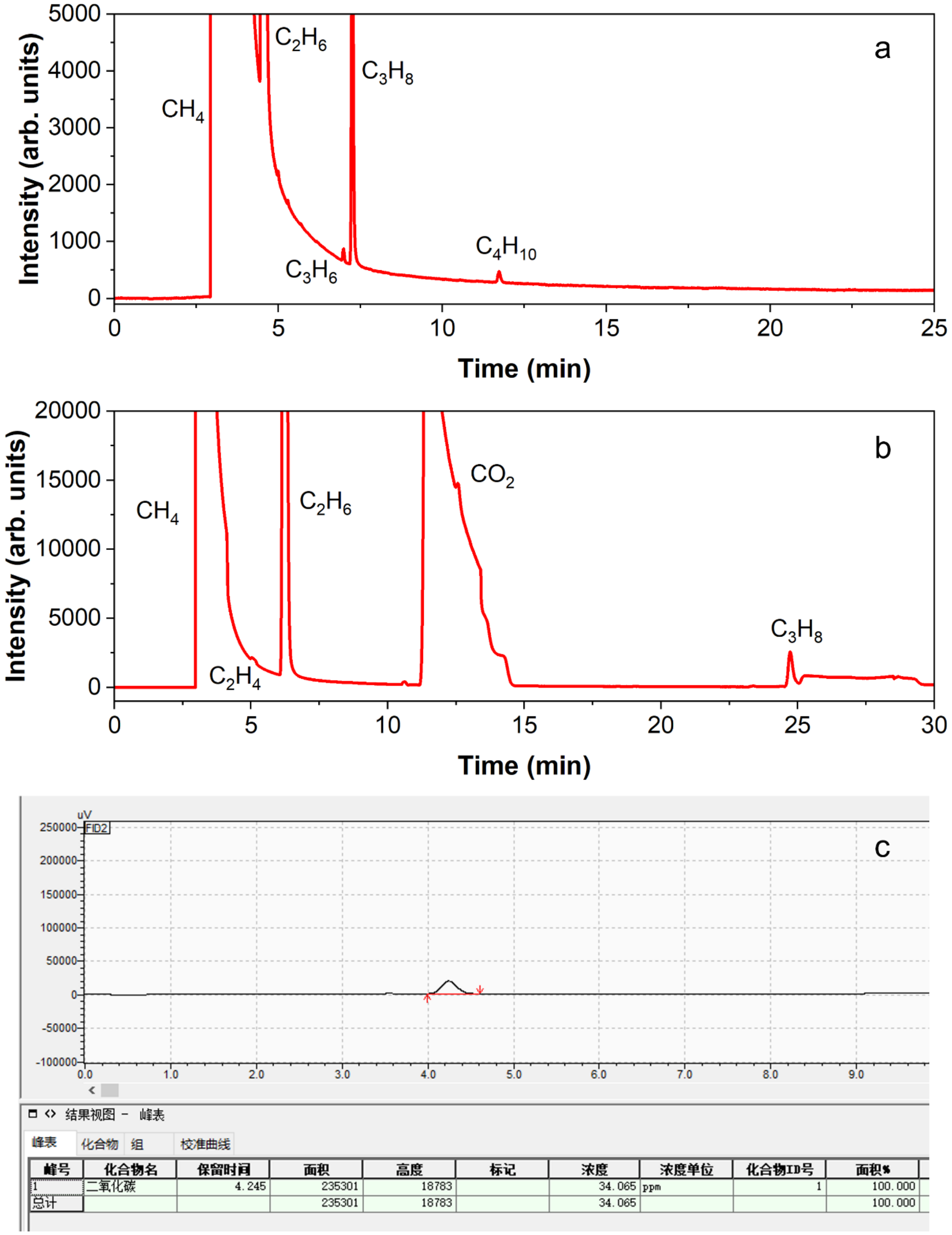

Supplementary Fig. 4 GC chromatography. GC spectra showing the production of C_2H_6, C_2H_4, C_3H_8, C_3H_6, C_4H_{10} and CO_2 over Au-CeO_2-300. C_3H_6, C_3H_8 and C_4H_{10} were quantified (a) and C_2H_4, and C_2H_6 were quantified (b). CO_2 was seperately quantified using another GC machine (c). Reaction conditions: 50 mg catalyst, methane to air = 200:1, GHSV = 480 000 mL h^{-1} g^{-1}, Pressure = 5 bar, Temperature = 150 °C, 365 nm LED, light intensity = 200 mW cm^{-2}.

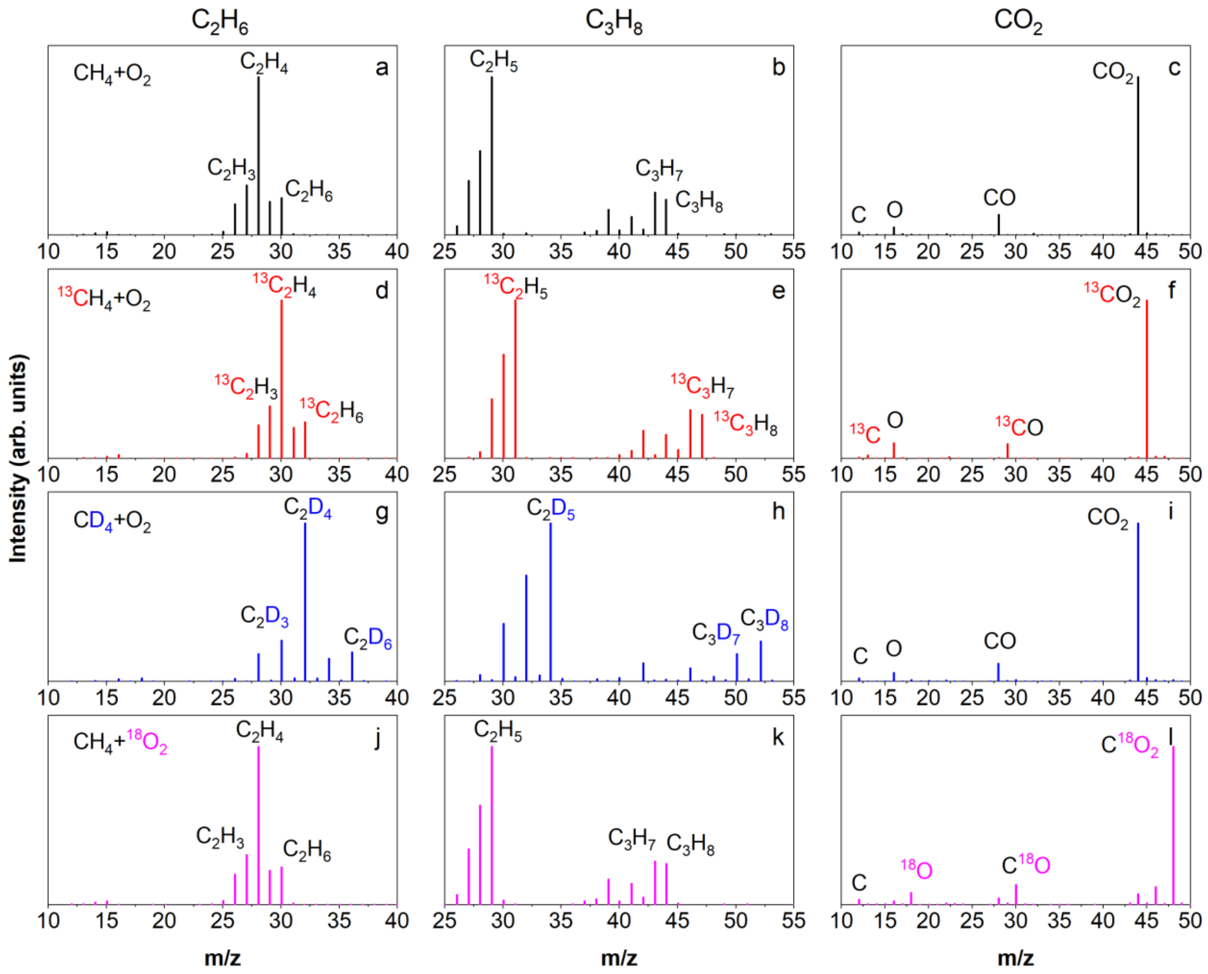

Supplementary Fig. 5 Isotopica labelling results. MS spectra of main products obtained from photocatalytic OCM by Au-CeO_2-300 using (a-c) CH_4+O_2, (d-f) $^{13}CH_4$+O_2, (g-i) CD_4+O_2, and (j-l) CH_4+$^{18}O_2$ as feedstock.

The MS spectra of C_2H_6, C_3H_8, and CO_2 obtained from non-labelled feedstock were firstly measured (Supplementary Fig. 5 a-c). When $^{13}CH_4$+O_2 was applied, all characteristic peaks of C_2H_6, C_3H_8, and CO_2 shift to a higher m/z value (Supplementary Fig. 5 d-f). In detail, the m/z ratios of C_2H_6 and C_2H_4 fragments of C_2H_6 increase from 30 and 28 to 32 and 30, respectively. The C_3H_7 and C_2H_5 fragments of C_3H_8 also shift from m/z of 43 and 29 to 46 and 31, respectively. The m/z ratio of the CO_2 main peak increases from 44 to 45. These indicate that C atoms in C_2H_6, C_3H_8, and CO_2 are from CH_4 in the feedstock. When the reactant was changed to CD_4+O_2, right shift of peaks of C_2H_6 and C_3H_8 is detected, while similar CO_2 signals are observed compared to non-labelled feedstock (Supplementary Fig. 5 g-i). The value of the right shift is consistent with the number of H atoms in the fragments, suggesting that H atoms in the produced C_{2+} hydrocarbons are also from CH_4. Finally, when CH_4+$^{18}O_2$ was used as feedstock (Supplementary Fig. 5 j-l), the peaks of C_2H_6 and C_3H_8 are similar to those using non-labelled feedstock. While the main peak of CO_2 shifts from 44 to 48. This suggests that the main oxygen source in CO_2 is oxygen gas. A small portion of CO_2 molecules with m/z rations of 46 and 44 are also detected, indicating possible oxygen exchange between the oxide catalyst and oxygen gas.

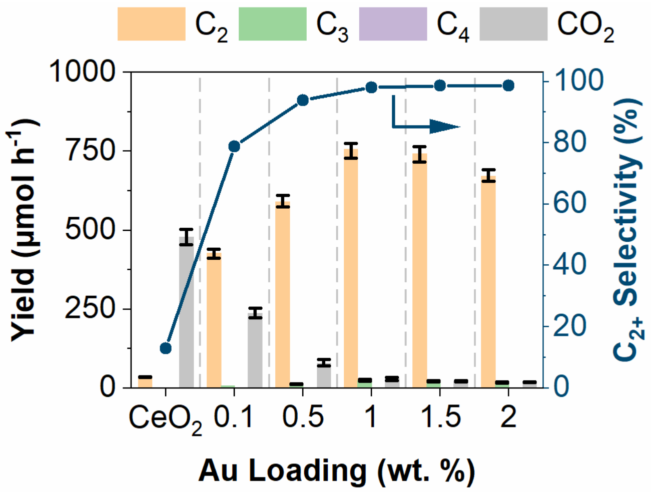

Supplementary Fig. 6 Effects of Au loading. Product yield and C_{2+} selectivity of Au-CeO_2 with different loading amounts of Au. Error bars represent standard deviations calculated from the performance tests of the photocatalysts prepared in three different batches. Reaction conditions: 50 mg catalyst, methane to air = 200:1, GHSV = 480 000 mL h^{-1} g^{-1}, Pressure = 5 bar, Temperature = 150 °C, 365 nm LED, light intensity = 200 mW cm^{-2}.

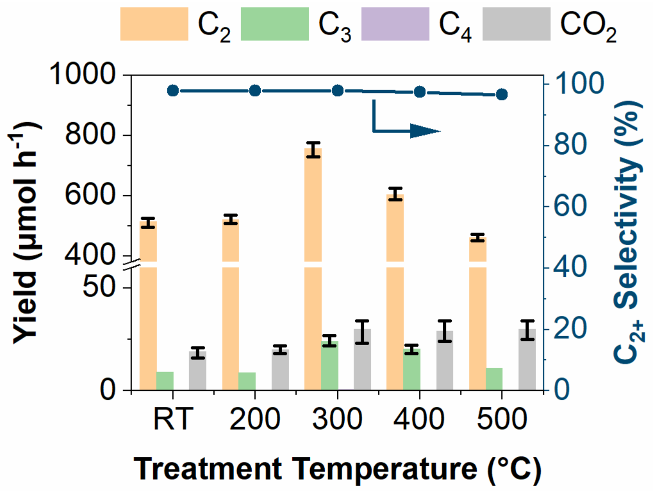

Supplementary Fig. 7 Effects of pretreatment temperature. Product yield and C_{2+} selectivity of Au-CeO_2 pre-treated under various temperatures. Error bars represent standard deviations calculated from the performance tests of the photocatalysts prepared in three different batches. Reaction conditions: 50 mg catalyst, methane to air = 200:1, GHSV = 480 000 mL h^{-1} g^{-1}, Pressure = 5 bar, Temperature = 150 °C, 365 nm LED, light intensity = 200 mW cm^{-2}.

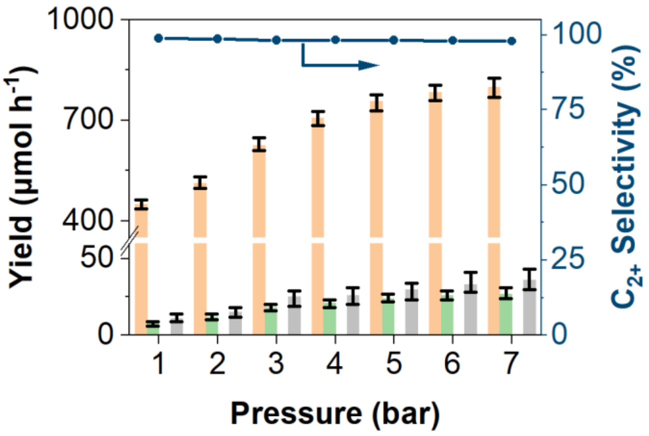

Supplementary Fig. 8 Effects of reaction pressure. Product yield and C_{2+} selectivity of Au-CeO_2-300 tested under different reaction pressures. Error bars represent standard deviations calculated from the performance tests of the photocatalysts prepared in three different batches. Reaction conditions: 50 mg catalyst, methane to air = 200:1, GHSV = 480 000 mL h^{-1} g^{-1}, 150 °C, 365 nm LED, light intensity = 200 mW cm^{-2}.

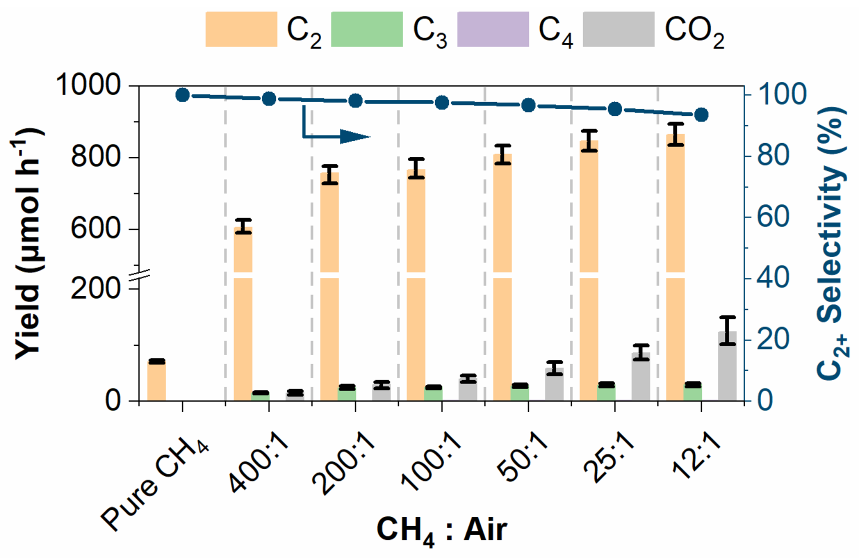

Supplementary Fig. 9 Effects of CH_4 to air ratio. Product yield and C_{2+} selectivity of Au-CeO_2-300 tested under different methane-to-air ratios. Error bars represent standard deviations calculated from the performance tests of the photocatalysts prepared in three different batches. Reaction conditions: 50 mg catalyst, GHSV = 480 000 mL h^{-1} g^{-1}, Pressure = 5 bar, Temperature = 150 °C, 365 nm LED, light intensity = 200 mW cm^{-2}.

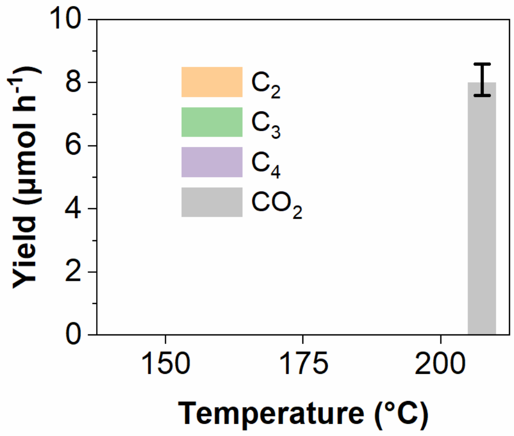

Supplementary Fig. 10 Thermal catalytic performance. Product yield of Au-CeO_2 for catalytic methane conversion in dark at different temperatures. Error bars represent standard deviations calculated from the performance tests of the photocatalysts prepared in three different batches. Reaction conditions: Reaction conditions: 50 mg catalyst, methane to air = 200:1, GHSV = 480 000 mL h^{-1} g^{-1}, Pressure = 5 bar, dark.

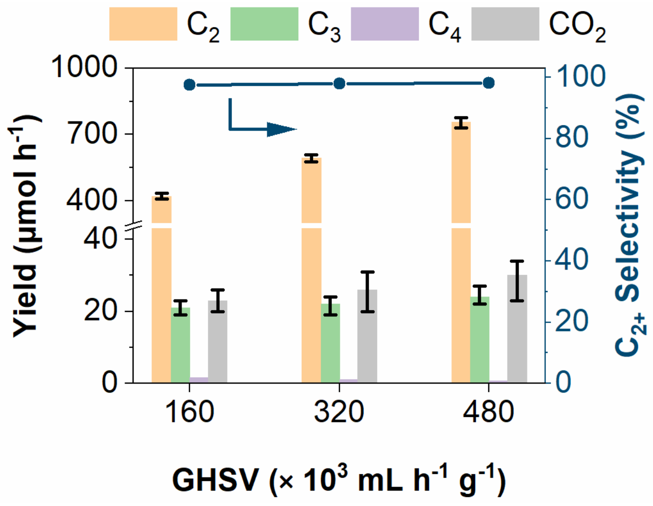

Supplementary Fig. 11 Effects of GHSV. Product yield and C_{2+} selectivity of Au-CeO_2-300 tested at different GHSVs. Error bars represent standard deviations calculated from the performance tests of the photocatalysts prepared in three different batches. Reaction conditions: 50 mg catalyst, methane to air = 200:1, Pressure = 5 bar, Temperature = 150 °C, 365 nm LED, light intensity = 200 mW cm^{-2}.

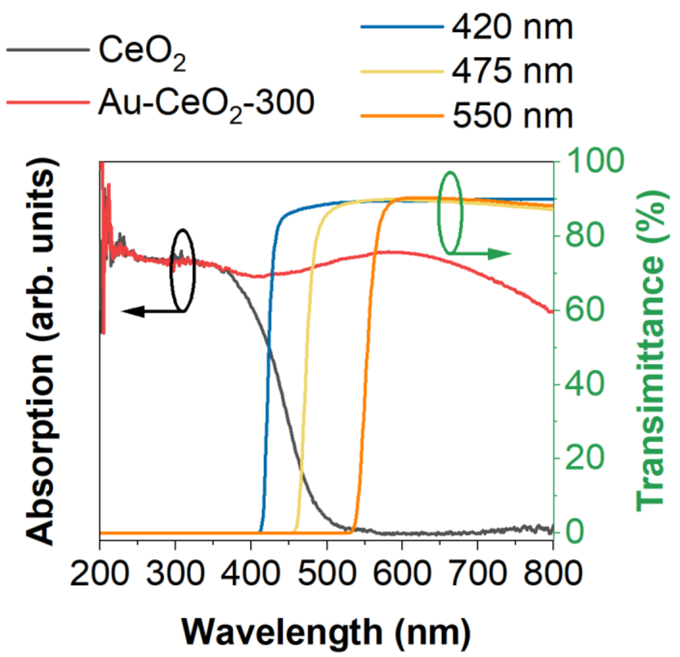

Supplementary Fig. 12 Transmittance of filters. UV-Vis absorption spectra of CeO_2 and Au-CeO_2-300, and transmittance spectra of the 420 nm, 475 nm, and 550 nm long-pass filters.

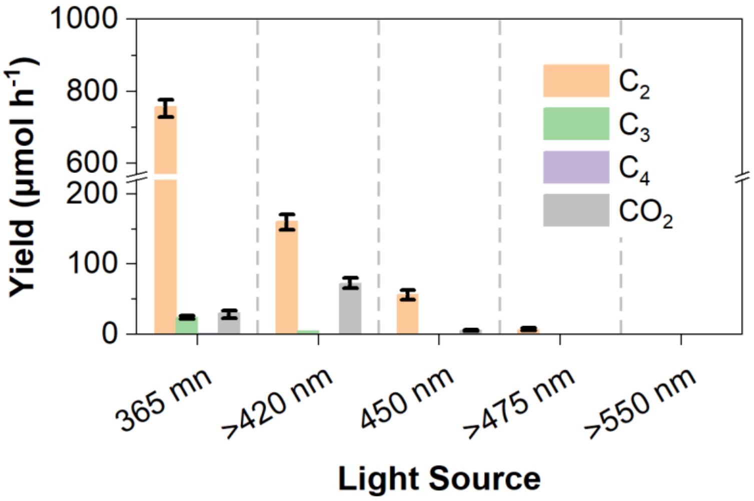

Supplementary Fig. 13 Wavelength-dependent performance. Product yield of Au-CeO_2-300 tested under different light sources, including a 200 mW cm^{-2} 365 nm LED, a 300 W Xe lamp with a 420 nm long-pass filter, a 200 mW cm^{-2} 450 nm LED, and a Xe lamp with a 475 and 550 nm long-pass filter. Error bars represent standard deviations calculated from the performance tests of the photocatalysts prepared in three different batches. Reaction conditions: 50 mg catalyst, methane to air = 200:1, GHSV = 480 000 mL h^{-1} g^{-1}, Pressure = 5 bar, Temperature = 150 °C.

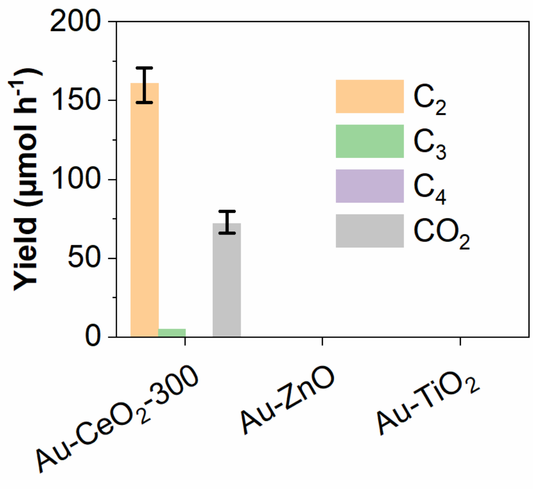

Supplementary Fig. 14 Performance under visible light. Product yield of Au-CeO_2-300, Au-ZnO, and Au-TiO_2 tested under a 300 W Xe lamp with a 420 nm long-pass filter. Error bars represent standard deviations calculated from the performance tests of the photocatalysts prepared in three different batches. Reaction conditions: 50 mg catalyst, methane to air = 200:1, GHSV = 480 000 mL h^{-1} g^{-1}, Pressure = 5 bar, Temperature = 150 °C.

Supplementary Table 1 Performance summary. A summary of the photocatalytic oxidative coupling of methane over the photocatalysts used in this work under various reaction conditions.

Catalyst	CH_4:Air	P/ bar	T /°C	GHSV/ mL h^{-1} g^{-1}	Light Source	Product Yield / μmol h^{-1}						S_{C2+} /%
						C_2H_6	C_2H_4	C_3H_8	C_3H_6	C_4	CO_2	
CeO_2	200:1	5	150	480 000	365 nm 200 mW cm^{-2}	34±2	0	0.3	0.4	0.2	478±26	13
Au1-CeO_2-RT	200:1	5	150	480 000	365 nm 200 mW cm^{-2}	512±18	1.2	8.2	0.4	0.2	19±3	98
Au1-CeO_2-200	200:1	5	150	480 000	365 nm 200 mW cm^{-2}	520±15	1.0	8.4	0.5	0.2	20±2	98
Au1-CeO_2-300	200:1	5	150	480 000	365 nm 200 mW cm^{-2}	755±28	2.5	23.7	0.7	0.7	30±7	98
Au1-CeO_2-400	200:1	5	150	480 000	365 nm 200 mW cm^{-2}	601±21	1.9	19.6	0.6	0.5	29±5	97
Au1-CeO_2-500	200:1	5	150	480 000	365 nm 200 mW cm^{-2}	459±11	1.7	10.9	0.3	0.4	30±5	97
Au0.1-CeO_2-300	200:1	5	150	480 000	365 nm 200 mW cm^{-2}	427±16	0.8	8.3	0.1	0.2	234±17	79
Au0.5-CeO_2-300	200:1	5	150	480 000	365 nm 200 mW cm^{-2}	589±21	1.3	11.2	0.5	0.3	79±12	94
Au1.5-CeO_2-300	200:1	5	150	480 000	365 nm 200 mW cm^{-2}	739±25	2.4	22.0	0.7	0.6	22±2	99
Au2-CeO_2-300	200:1	5	150	480 000	365 nm 200 mW cm^{-2}	669±20	1.8	16.3	0.7	0.5	18±2	99
Au1-CeO_2-300	400:1	5	150	480 000	365 nm 200 mW cm^{-2}	603±21	1.8	15.0	0.4	0.4	15±3	99
Au1-CeO_2-300	100:1	5	150	480 000	365 nm 200 mW cm^{-2}	763±30	2.8	24.4	0.7	1.1	39±6	98
Au1-CeO_2-300	50:1	5	150	480 000	365 nm 200 mW cm^{-2}	806±25	3.2	27.0	0.7	1.2	57±12	97
Au1-CeO_2-300	25:1	5	150	480 000	365 nm 200 mW cm^{-2}	842±29	3.4	28.8	0.5	1.2	86±14	95
Au1-CeO_2-300	12:1	5	150	480 000	365 nm 200 mW cm^{-2}	859±31	4.3	29.5	0.6	1.1	124±26	94
Au1-CeO_2-300	200:1	1	150	480 000	365 nm 200 mW cm^{-2}	447±14	1.4	8.0	0.2	0.1	11±3	99
Au1-CeO_2-300	200:1	2	150	480 000	365 nm 200 mW cm^{-2}	511±18	1.7	11.2	0.4	0.3	15±3	99
Au1-CeO_2-300	200:1	3	150	480 000	365 nm 200 mW cm^{-2}	622±22	2.7	17.6	0.7	0.4	25±6	98
Au1-CeO_2-300	200:1	4	150	480 000	365 nm 200 mW cm^{-2}	704±22	2.8	20.3	0.6	0.5	26±6	98
Au1-CeO_2-300	200:1	6	150	480 000	365 nm 200 mW cm^{-2}	781±25	2.7	25.5	1.0	0.7	33±8	98
Au1-CeO_2-300	200:1	7	150	480 000	365 nm 200 mW cm^{-2}	795±31	2.6	26.3	1.0	0.7	36±7	98
Au1-CeO_2-300	200:1	5	50	480 000	365 nm 200 mW cm^{-2}	317±13	0	3.4	0	0.8	13±2	98
Au1-CeO_2-300	200:1	5	100	480 000	365 nm 200 mW cm^{-2}	568±18	0.3	17.0	0.3	0.9	19±3	98
Au1-CeO_2-300	200:1	5	200	480 000	365 nm 200 mW cm^{-2}	792±25	6.5	18.5	0.3	0.3	91±10	95
Au1-CeO_2-300	200:1	5	150	160 000	365 nm 200 mW cm^{-2}	419±14	1.5	20.0	1.3	1.6	23±3	98

Au1-CeO_2-300	200:1	5	150	320 000	365 nm 200 mW cm^{-2}	591±16	1.7	21.0	1.0	1.1	26±6	98
Au1-CeO_2-300	200:1	5	150	480 000	365 nm 10 mW cm^{-2}	52±2	0	0	0	0	0	100
Au1-CeO_2-300	200:1	5	150	480 000	365 nm 25 mW cm^{-2}	113±3	0	0	0	0	0	100
Au1-CeO_2-300	200:1	5	150	480 000	365 nm 50 mW cm^{-2}	215±7	0	4.3	0	0	4	99
Au1-CeO_2-300	200:1	5	150	480 000	365 nm 100 mW cm^{-2}	416±13	0.5	9.4	0	0.2	11±3	99
Au1-CeO_2-300	200:1	5	150	480 000	365 nm 150 mW cm^{-2}	551±18	1.6	12.4	0	0.3	21±3	98
Au1-CeO_2-300	200:1	5	90	480 000	300 W Xe Lamp, AM1.5	104±6	0.2	4.5	0	0	51±6	82
Au1-CeO_2-300	200:1	5	150	480 000	300 W Xe Lamp, > 420 nm	158±12	2.5	5.4	0	0.2	72±8	81
Au1-CeO_2-300	200:1	5	150	480 000	450 nm 200 mW cm^{-2}	57±8	0	0	0	0	5±1	96

Supplementary Table 2 Performance comparison. A comparison of the photocatalytic methane oxidation performance of Au-CeO_2-300 with the recent literature, $S_{C_{2+}}$ represents the selectivity of C_{2+} products.

Catalyst	Reaction Conditions	C_2H_6 Yield (µmol h^{-1})	$S_{C_{2+}}$	AQY	Stability test	Ref.
**Au-CeO_2-300**	**Flow reactor, GHSV=480 000 mL h^{-1} g^{-1}, CH_4 : air =200:1, 365 nm LED light, 200 mW cm^{-2}, 50 mg catalyst, 5 bar, 150 °C.**	**755**	**98%**	**12% at 365 nm**	**120 h**	**This work**
Au-ZnO/TiO_2	Flow reactor, GHSV=210 000 mL h^{-1} g^{-1}, CH_4 : air=69:1, 300 W Xe lamp, 20 mg catalyst, 1 bar, 140 °C.	100	95%	7.2% at 360 nm	12 h	[15]
Pd1/TiO_2	Batch reactor, pure CH_4, 300 W Xe lamp, 3 mg catalyst, 10 bar.	2.7	94.3%	3.1% at 350 nm	24 h	[16]
Ag-AgBr/TiO_2	Batch reactor, GHSV=240 000 mL h^{-1} g^{-1}, CH_4 : air=40:1, 365 nm LED, 100 mW cm^{-2}, 100 mg catalyst, 6 bar, 40 °C	35.4	79%	3% at 365 nm	12 h	[17]
Pt/Ga_2O_3	Flow reactor, 240 000 mL h^{-1} g^{-1}, pure CH_4, 40 W Hg lamp, 50 mg catalyst, 3 bar.	36	67%	13% at 254 nm	22 h	[18]
Pd/Ga_2O_3	Flow reactor, 480 000 mL h^{-1} g^{-1}, CH_4 + water vapour, 40 W Hg lamp, 50 mg catalyst, 4 bar, 25 °C.	67	83%	14% at 254 nm	4 h	[19]
Pt-CuO_x/TiO_2	Flow reactor, GHSV= 1200 h^{-1}, CH_4 : air=80:1, 365 nm LED, 160 mW cm^{-2}, 100 mg catalyst, 1 bar.	6.8	60%	0.5% at 365 nm	8 h	[20]
Ag/$NaTaO_3$	Batch reactor, pure CH_4, 254 nm UV lamp, 8 W, 10 mg catalyst.	1.9	97%	11% at 254 nm	4 cycles, 8 h	[21]
Ag-HPW/TiO_2	Batch reactor, pure CH_4, 400 W Xe lamp, 100 mg catalyst, 3 bar.	2.3	90%	3.5% at 362 nm	10 cycles, 75 h	[22]
AuPd/ZnO	Batch reactor, pure CH_4, 300 W Xe lamp, 2 mg catalyst.	0.05	97%	-	5 cycles, 20 h	[23]
Pd-Bi/Ga_2O_3	Flow reactor, GHSV=4500 h^{-1}, 10% CH_4 in Ar, 300 W Xe lamp, 800 mg catalyst.	0.96	96%	0.2% at 220-300 nm	100 h	[24]
GaN:ZnO	Batch reactor, pure CH_4, 300 W Xe lamp (300-400 nm), 2 mg catalyst, 20 °C.	0.7	>89%	0.7% at 325 nm	35 cycles, 70 h	[25]
Au/Ga_2O_3	Flow reactor, 480 000 mL h^{-1} g^{-1}, CH_4+water vapour, 40 W Hg lamp, 50 mg catalyst, 4 bar, 25 °C.	57	92%	4.3% at 254 nm	4 h	[26]
TiO_2	Batch reactor, CH4 + HSO_5^-, 300 W Xe lamp, 20 mg catalyst, 20 bar.	13	75%	-	-	[27]

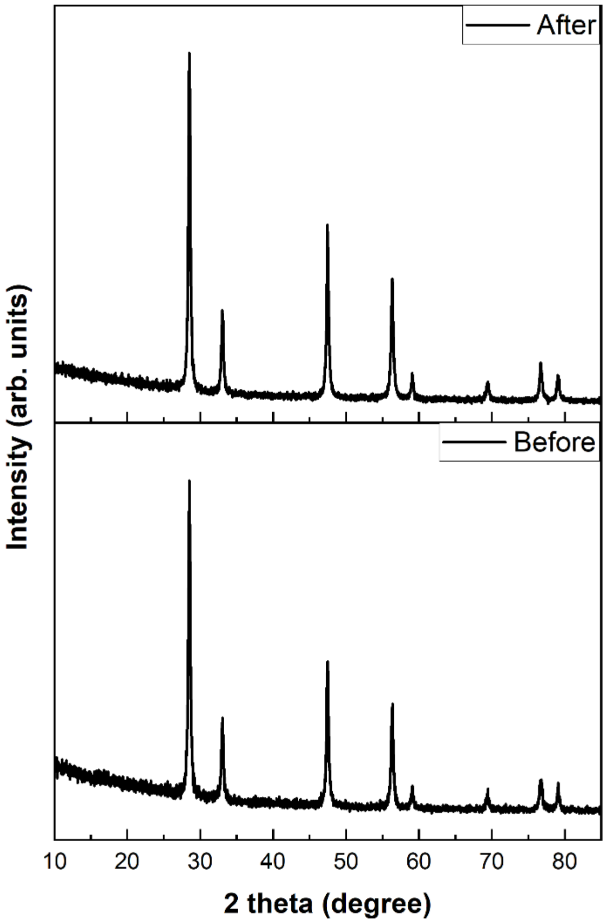

Supplementary Fig. 15 Structure after reaction. XRD patterns of Au-CeO_2-300 before and after photocatalytic methane oxidation for 120 h.

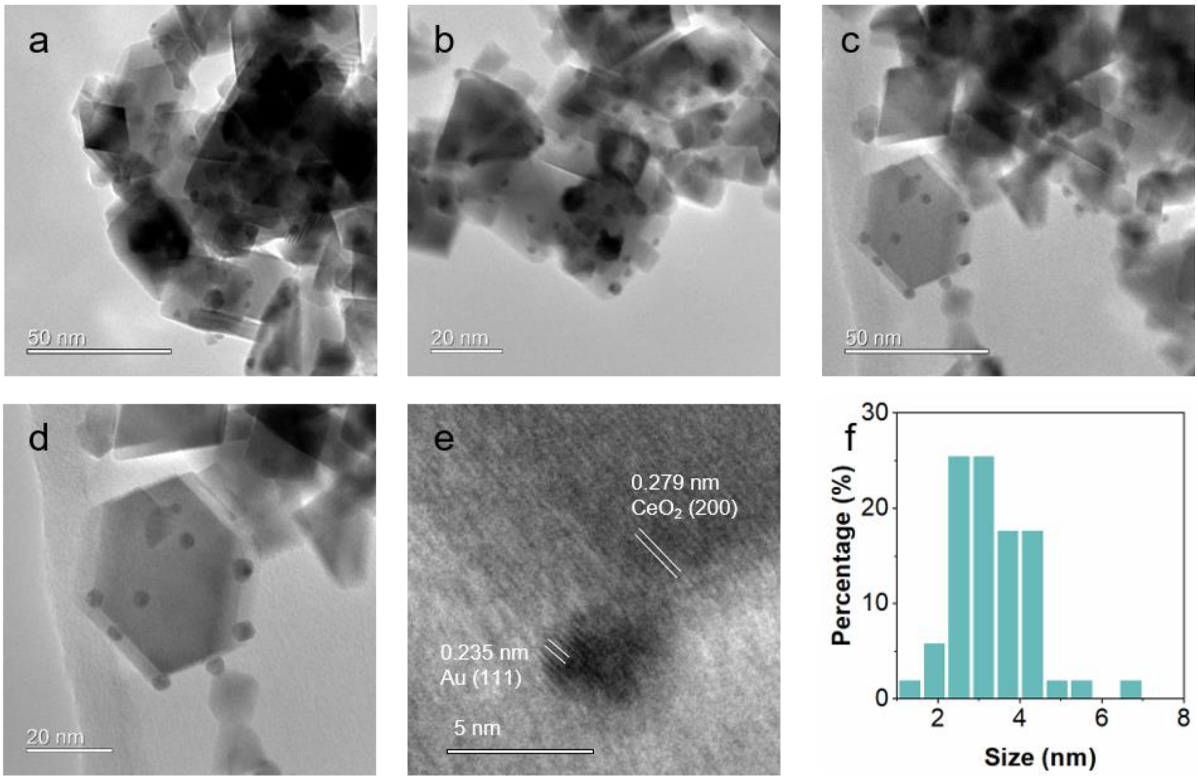

Supplementary Fig. 16 Morphology after reaction (a-e) TEM images and (f) Au particle size distribution of Au-CeO_2-300 after photocatalytic methane oxidation for 120 h.

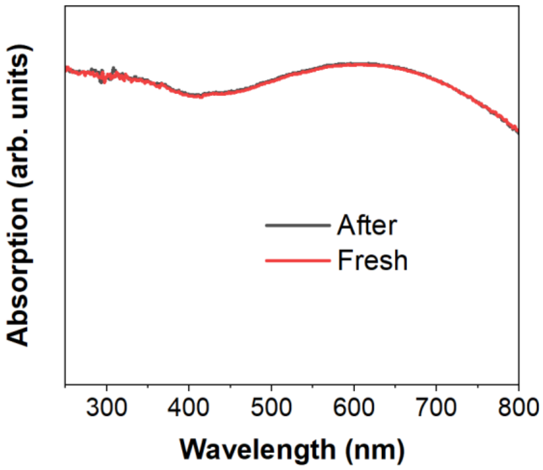

Supplementary Fig. 17 Absorption after reaction. UV-Vis DRS spectra of Au-CeO_2-300 before and after photocatalytic methane oxidation for 120 h.

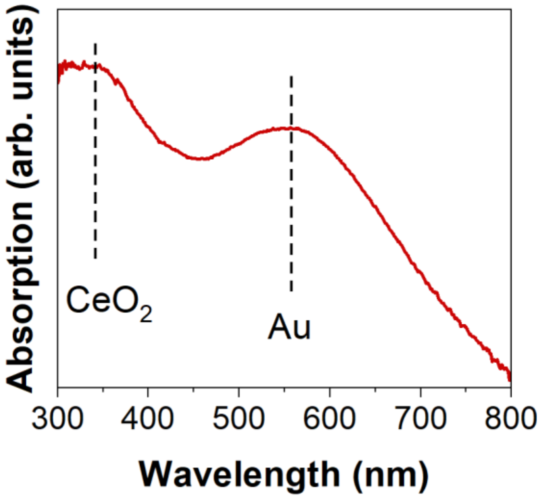

Supplementary Fig. 18 Plasmonic absorption of Au. UV-Vis absorption spectrum of Au0.1-CeO_2-300.

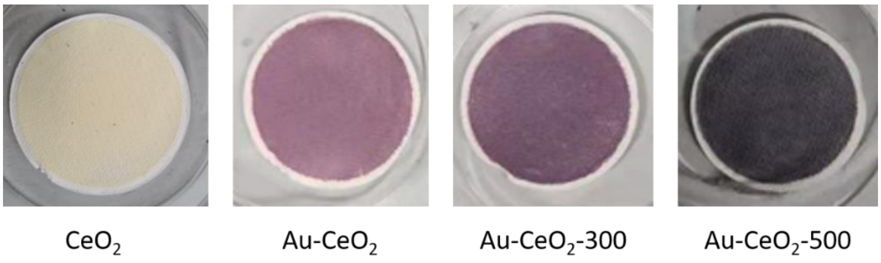

Supplementary Fig. 19 Photos of catalyst. Optical images of CeO_2, Au-CeO_2, Au-CeO_2-300 and Au-CeO_2-500.

Supplementary Table 3 Surface temperatures. Surface temperatures of Au-CeO_2-300 measured by an infrared radiative thermometer under irradiation conditions with and without external heating.

Light source	Without external heating	Heating set to 150 °C
365 nm LED	83 °C	152 °C
450 nm LDE	95 °C	157 °C

Due to the strong absorption of Au-CeO_2-300 across the UV-Vis spectrum, the temperature of the catalyst surface may increase under irradiation due to the photothermal effect. Thus, the surface temperatures of Au-CeO_2-300 with and without external heating under various light sources were measure. The surface temperatures range from 80 to 110 °C or from 150 to 160 °C under conditions without or with external heating, respectively.

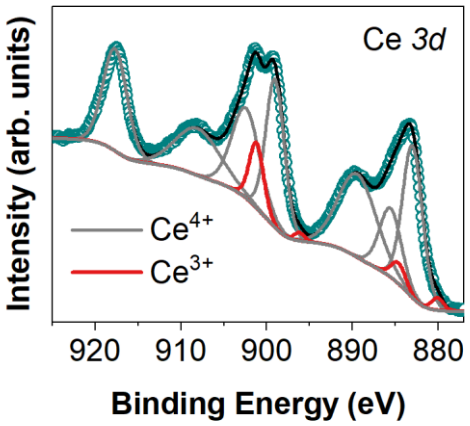

Supplementary Fig. 20 Ce *3d* XPS spectrum of CeO_2.

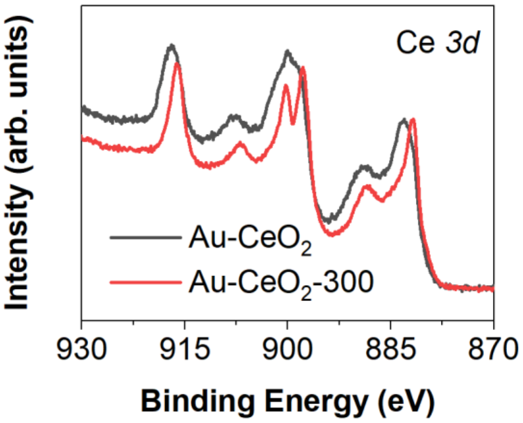

Supplementary Fig. 21 Ce *3d* XPS spectra of Au-CeO_2 and Au-CeO_2-300.

Supplementary Table 4 Peak areas of XPS bands. Peak areas of fitted bands for Ce *3d* XPS spectra and calculated Ce^{3+} ratios of CeO_2, Au-CeO_2, Au-CeO_2-300, and Au-CeO_2-500.

Sample	Ce^{4+} Binding Energy (eV)							Ce^{3+} Binding Energy (eV)				Ce^{3+} Ratio
	881	885	888	898	902	907	917	879	883	896	900	
CeO_2	146678	61850	152603	131373	121586	100766	117466	7044	21020	4162	45854	8.5%
Au-CeO_2	60389	38857	55318	53118	62778	30890	49389	2019	2083	0	13744	4.8%
Au-CeO_2-300	59724	22479	61366	83318	35602	34990	62996	13865	50459	4606	49079	24.6%
Au-CeO_2-500	31097	15752	29286	46221	20175	21054	37668	10798	34789	2236	33905	28.9%

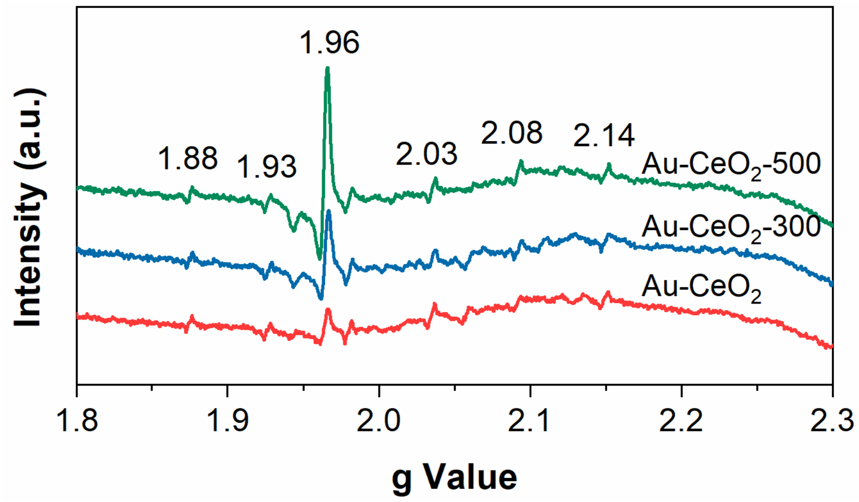

Supplementary Fig. 22 EPR spectra of Au-CeO_2, Au-CeO_2-300, and Au-CeO_2-500.

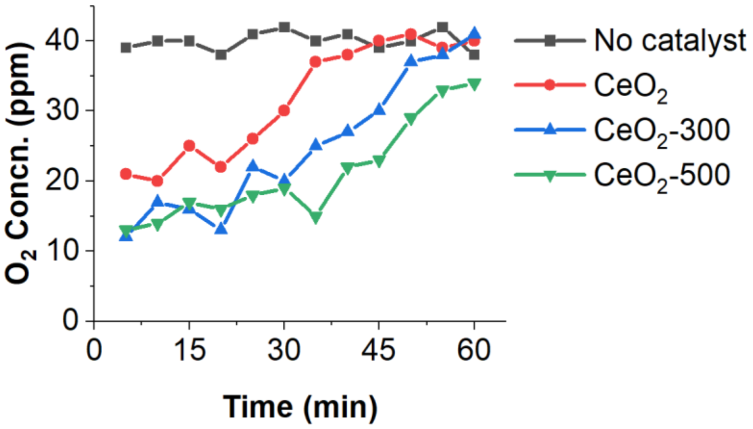

Supplementary Fig. 23 O_2 adsorption over CeO_2, CeO_2-300, and CeO_2-500.

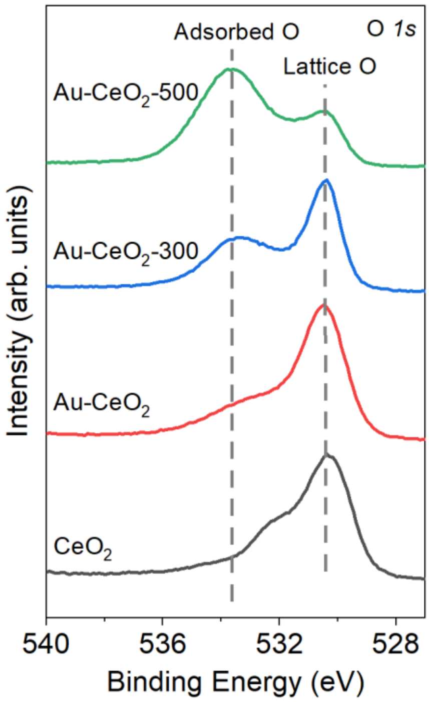

Supplementary Fig. 24 O *1s* XPS spectra of CeO_2, Au-CeO_2, Au-CeO_2-300 and Au-CeO_2-500.

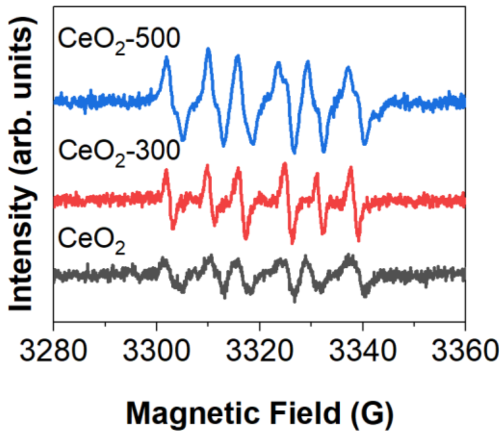

Supplementary Fig. 25 EPR spectra of CeO_2, CeO_2-300, and CeO_2-500 for O_2^- trapping.

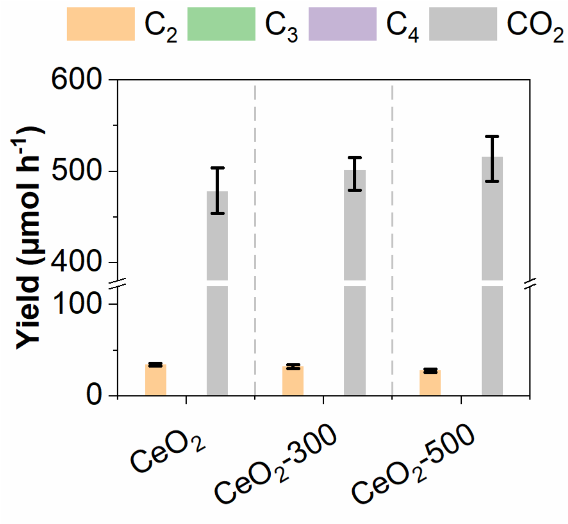

Supplementary Fig. 26 Product yield of pre-treated CeO_2. Photocatalytic methane conversion performance of CeO_2, CeO_2-300 and CeO_2-500. Error bars represent standard deviations calculated from the performance tests of the photocatalysts prepared in three different batches. Reaction conditions: 50 mg catalyst, methane to air = 200:1, GHSV = 480 000 mL h^{-1} g^{-1}, Pressure = 5 bar, Temperature = 150 °C, 365 nm LED, light intensity = 200 mW cm^{-2}.

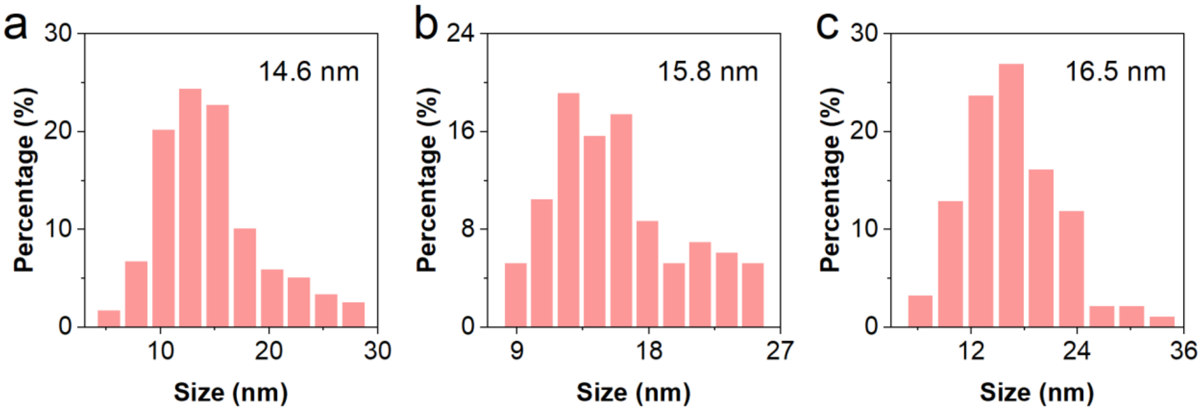

Supplementary Fig. 27 Size of CeO_2. Size distribution of CeO_2 in (a) Au-CeO_2, (b) Au-CeO_2-300 and (c) Au-CeO_2-500.

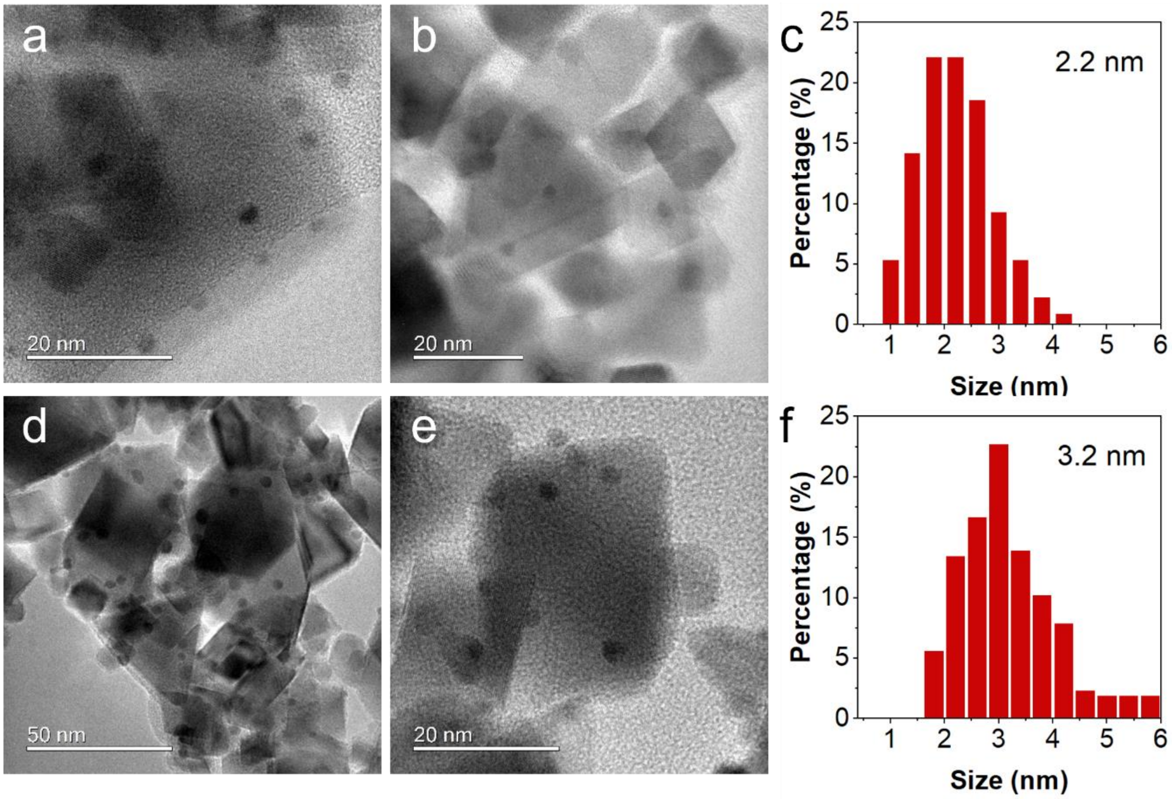

Supplementary Fig. 28 Morphology of Na modified Au-CeO_2 catalysts. TEM images of Au size distribution of (a-c) Au-CeO_2-Na and (d-f) Au-CeO_2-Na-300.

It has been reported that single atoms and small nanoclusters of metal catalysts display lower performance in oxidation reactions compared with relatively large nanoparticles.[28–30] To investigate this, smaller Au species were loaded onto CeO_2 by a modified $NaBH_4$ reduction method.[31] The catalyst obtained is denoted Au-CeO_2-Na. Au-CeO_2-Na was also calcined at 300 °C to obtain Au-CeO_2-Na-300. The TEM images of Au-CeO_2-Na display a large portion of Au clusters on CeO_2 (Supplementary Fig. 27 a and b). The average Au size on Au-CeO_2-Na is 2.2 nm, smaller than that on Au-CeO_2 of 3.1 nm. After calcination at 300 °C, the average Au size on Au-CeO_2-Na-300 increases (Supplementary Fig. 27 d and e) to 3.2 nm, similar to that of Au-CeO_2-300 (3.0 nm).

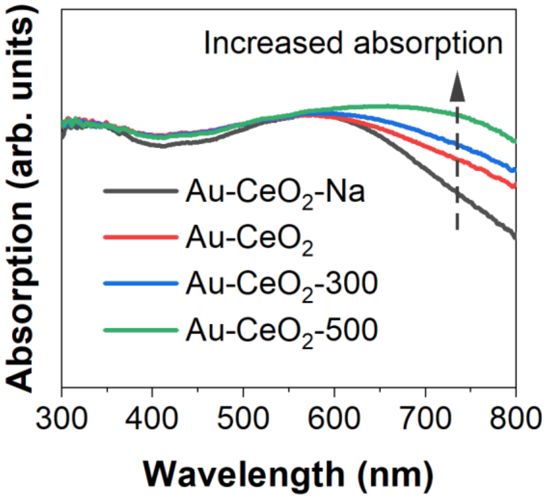

Supplementary Fig. 29 Light absorption of of Na modified Au-CeO_2 catalysts. UV-Vis absorption of Au-CeO_2-Na, Au-CeO_2, Au-CeO_2-300 and Au-CeO_2-500.

The UV-Vis absorption spectrum of Au-CeO_2-Na was measured, which displays the most blue-shifted Au plasmonic feature, indicating its smallest Au particle size among the four catalysts.

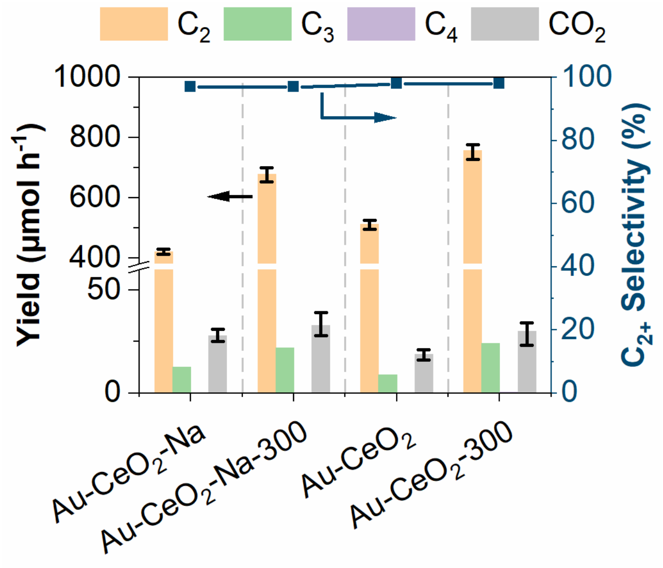

Supplementary Fig. 30 Product yield of different Au-CeO$_2$ catalysts. Photocatalytic methane conversion performance of Au-CeO$_2$-Na, Au-CeO$_2$-Na-300, Au-CeO$_2$ and Au-CeO$_2$-300. Error bars represent standard deviations calculated from the performance tests of the photocatalysts prepared in three different batches. Reaction conditions: 50 mg catalyst, methane to air = 200:1, GHSV = 480 000 mL h^{-1} g^{-1}, Pressure = 5 bar, Temperature = 150 °C, 365 nm LED, light intensity = 200 mW cm^{-2}.

The photocatalytic methane oxidation performance of the Au-CeO$_2$-Na and Au-CeO$_2$-Na-300 was then measured to study the effect of slightly increased Au particle size on the catalytic activity. Au-CeO$_2$-Na with the smallest Au average size (2.2 nm) displays a C_2H_6 yield of 421 µmol h^{-1}, lower than that of Au-CeO$_2$ (C_2H_6 yield of 513 µmol h^{-1}, Au size 3.1 nm). After calcined at 300 °C, the C_2H_6 yield of Au-CeO$_2$-Na-300 is improved to 681 µmol h^{-1}. The results indicate that Au particle size has an effect on photocatalytic methane oxidation.

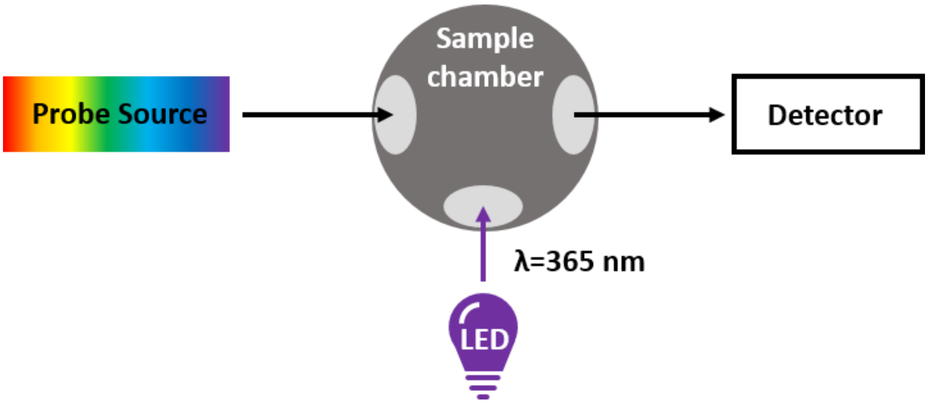

Supplementary Fig. 31 A diagram showing the setup used for PIA spectroscopy.

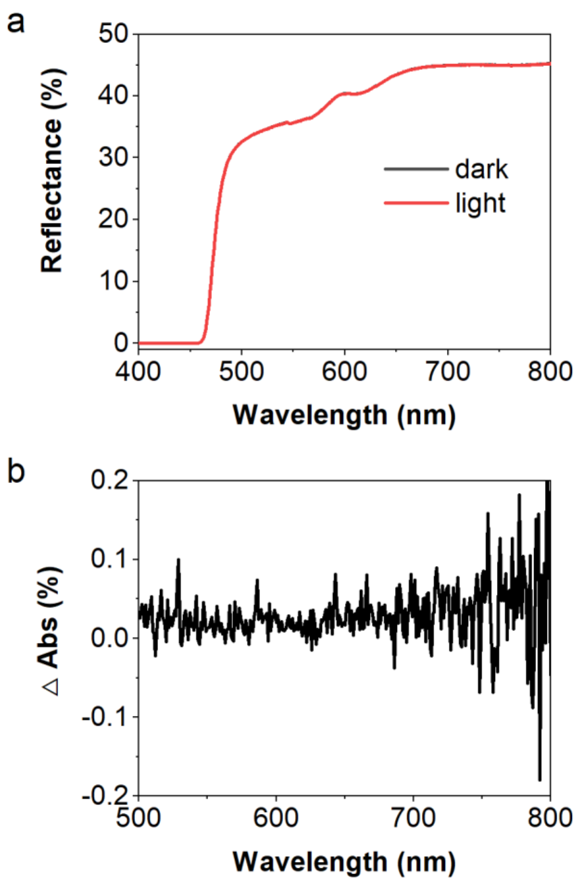

Supplementary Fig. 32 PIA of $BaSO_4$. (a) UV-Vis DRS spectrum of $BaSO_4$ in dark and under 365 nm irradiation, and (b) PIA spectrum of $BaSO_4$ in Ar atmosphere.

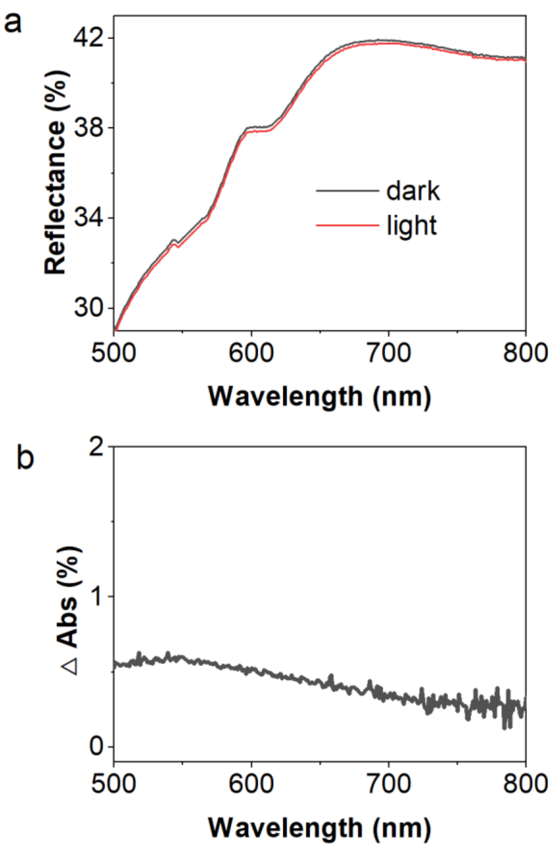

Supplementary Fig. 33 PIA of CeO_2. (a) UV-Vis DRS spectra of CeO_2 in dark and under 365 nm irradiation, and (b) PIA spectrum of CeO_2 in Ar atmosphere.

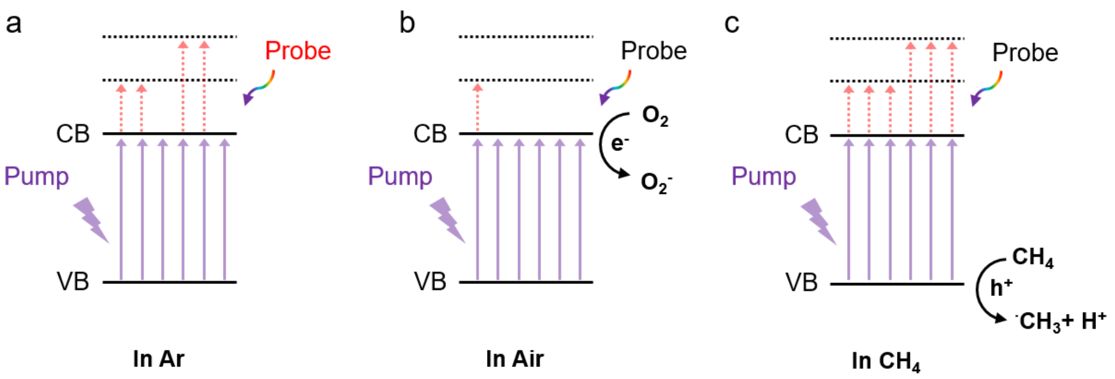

Supplementary Fig. 34 Charge migration process. Diagrams showing the charge migration of CeO_2 in (a) Ar, (b) Air, and (c) methane during PIA measurement.

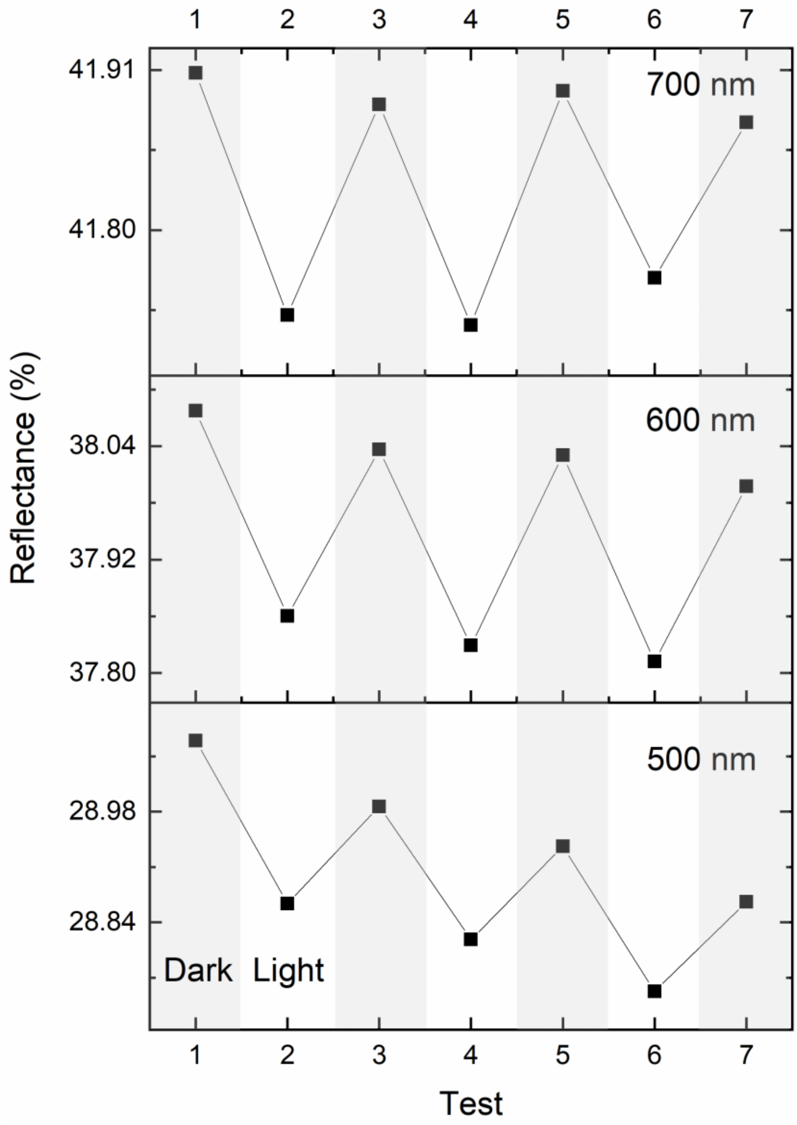

Supplementary Fig. 35 Reflectance change during PIA tests. Evolution of reflectance at 500, 600, and 700 nm over CeO_2 in Ar atmosphere under continuous light on-off switching.

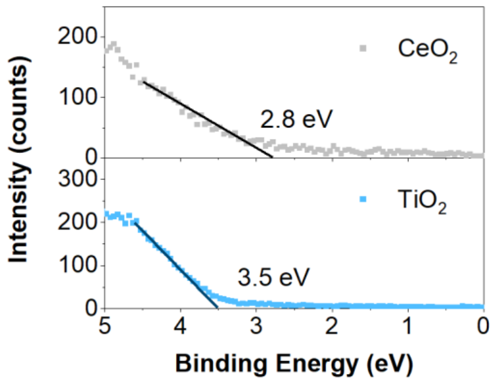

Supplementary Fig. 36 VB spectra. XPS VB spectra of CeO_2 and anatase TiO_2.

It has been reported that a minimum oxidation potential of 1.75 V vs. NHE is required to activate CH_4 into $CH_3\cdot$ and H^+.[22] Thus, the XPS VB spectrum of CeO_2, which could provide the relative oxidation potential of photo-generated holes at the VB of CeO_2, was measured. The VB spectrum of anatase TiO_2 was also measured as a reference. The interceptions obtained for CeO_2 and TiO_2 are 2.8 and 3.5 eV, respectively. This indicates that the VB potential of CeO_2 is 0.7 V more negative than that of TiO_2. Considering the reported VB potential of anatase is 2.9 V vs. NHE,[32] the VB potential of CeO_2 is calculated to be 2.2 V vs. NHE, which is more positive than the minimum methane oxidation potential of 1.75 V vs. NHE. Thus, the photo-holes generated at the VB of CeO_2 are theoretically capable of methane activation.

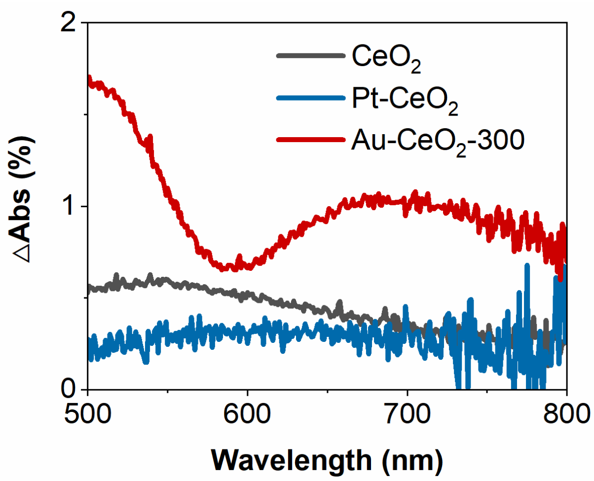

Supplementary Fig. 37 Effects of co-catalysts on the PIA of CeO_2. PIA spectra of CeO_2, Pt-CeO_2-300, and Au-CeO_2-300.

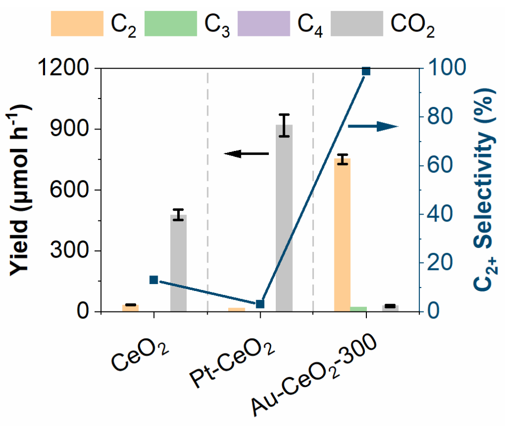

Supplementary Fig. 38 Effects of co-catalysts on the product yield of CeO_2. Photocatalytic methane conversion performance of CeO_2, Au-CeO_2-300 and Pt-CeO_2-300. Error bars represent standard deviations calculated from the performance tests of the photocatalysts prepared in three different batches. Reaction conditions: 50 mg catalyst, methane to air = 200:1, GHSV = 480 000 mL h^{-1} g^{-1}, Pressure = 5 bar, Temperature = 150 °C, 365 nm LED, light intensity = 200 mW cm^{-2}.

If Au promotes charge separation by trapping electrons from CeO_2 in a similar manner to Pt, the yield and selectivity of the products obtained should show a similar trend. However, CO_2 is the major product (97% selectivity) observed over Pt-CeO_2 as reported before over other photocatalysts,[15,20,33] while C_{2+} hydrocarbons remain the key products over Au-CeO_2-300. It is well known that Pt promotes electron separation from CeO_2 and boosts the oxygen reduction reaction, favouring the complete oxidation of methane. Therefore, both CeO_2 and Pt-CeO_2 selectively produce CO_2. In contrast, Au separates photo-generated holes from CeO_2 and reduces the oxidation potential of holes, leading to a retarded overoxidation process.

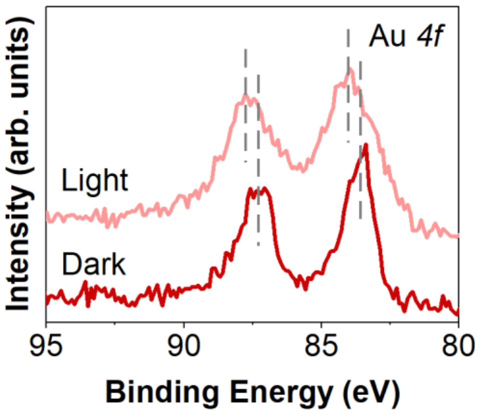

Supplementary Fig. 39 XPS under light irradiation. Au *4f* XPS spectra of Au-CeO_2-300 in dark and light irradiation.

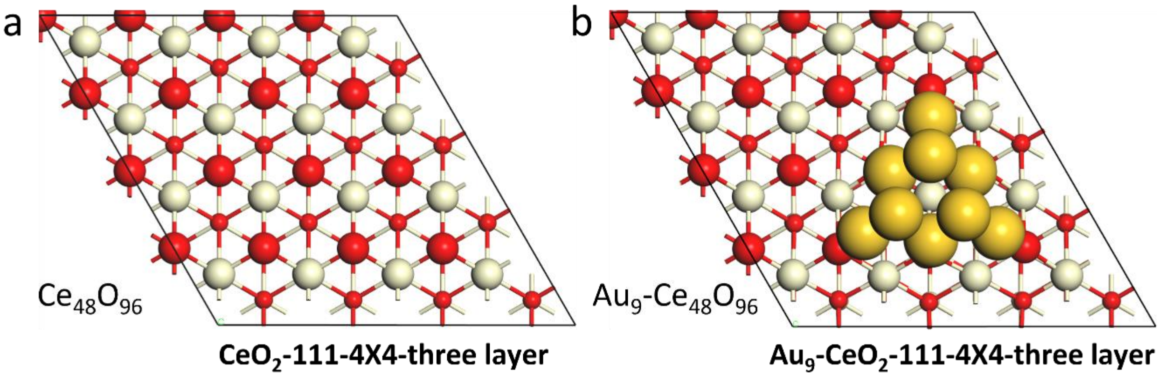

Supplementary Fig. 40 Models of ideal catalysts. The structures of (a) $Ce_{48}O_{96}$ and (b) Au_9-$Ce_{48}O_{96}$. Ce, O and Au are marked as red, white and orange.

A three-layer 4×4 CeO_2 (111) surface was chosen as the substrate (Supplementary Fig. 40a). A Au_9 cluster was loaded on CeO_2 (Supplementary Fig. 40b). The models of CeO_2 and Au-CeO_2-300 were then built based on $Ce_{48}O_{96}$ and Au_9-$Ce_{48}O_{96}$.

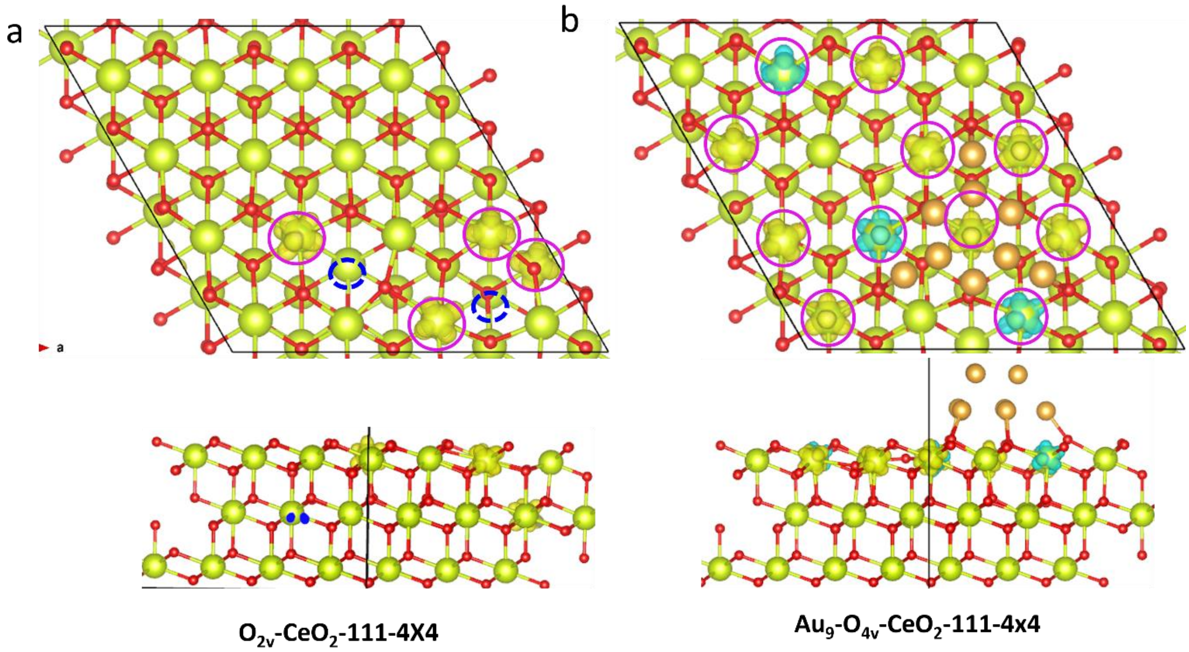

Supplementary Fig. 41 Models of actual catalysts The structures of (a) O_{2V}-CeO_2 and (b) Au_9-O_{4V}-CeO_2. Ce, O and Au are marked as yellow, red and orange. Ce^{3+} sites are marked with purple circles.

According to the XPS analysis, 8% and 24% of Ce^{3+} are detected in CeO_2 and Au-CeO_2-300, which corresponds to 4 and 11 Ce^{3+} in the CeO_2 and Au_9-CeO_2 models, respectively. Thus, the designated numbers of Ce^{3+} and O_V are introduced into both $Ce_{48}O_{96}$ and Au_9-$Ce_{48}O_{96}$. The obtained models are then used for calculation. The positions of O_V are optimised based on the lowest formation energies. The formation energy of O_V (E_{vac})in each site is calculated by:

$$E_{vac} = E_{Ov} + \frac{1}{2}E_{O_2} - E_{intergral}$$

Where E_{Ov} and $E_{intergral}$ represent the energies of the slab with and without the oxygen vacancy. E_{O_2} is the energy of the gas phase O_2.

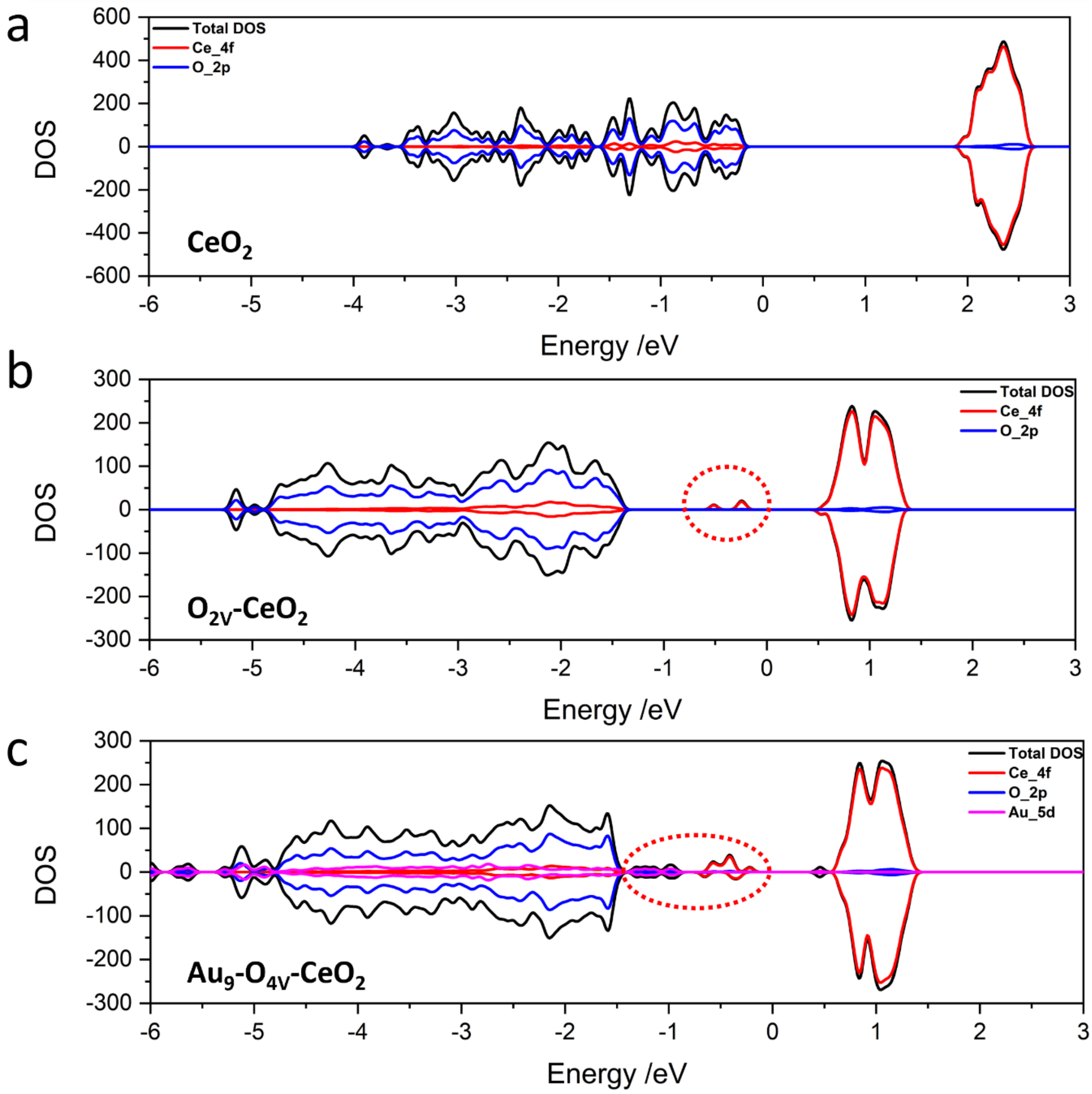

Supplementary Fig. 42 DOS spectra. Calculated density of states (DOS) for (a) perfect CeO_2, (b) O_{2V}-CeO_2, and (c) Au_9-O_{4V}-CeO_2. Contributions of DOS from Ce^{3+} and Au 5d are marked with red circles.

Based on the above models, the Density of States (DOS) of perfect CeO_2, O_{2v}-CeO_2 and Au_9-O_{4V}-CeO_2 are obtained. The VB maximum and CB minimum of perfect CeO_2 are contributed by the O 2p and Ce 4f orbitals, respectively (Supplementary Fig. 42a). For the O_{2v}-CeO_2 and Au_9-O_{4V}-CeO_2 system, the peak related to Ce 4f empty orbital shifts significantly towards the Fermi level and a new peak below the Fermi energy is found, related to the Ce^{3+} (Supplementary Fig. 42b and c). Both of these would promote charge migration. Moreover, in the Au_9-O_{4V}-CeO_2 system, Au 5d orbital (near the Ce^{3+} 4f orbital) may work as an electron donor (a hole acceptor) in Au_9-O_{4V}-CeO_2, which could further promote charge transfer.

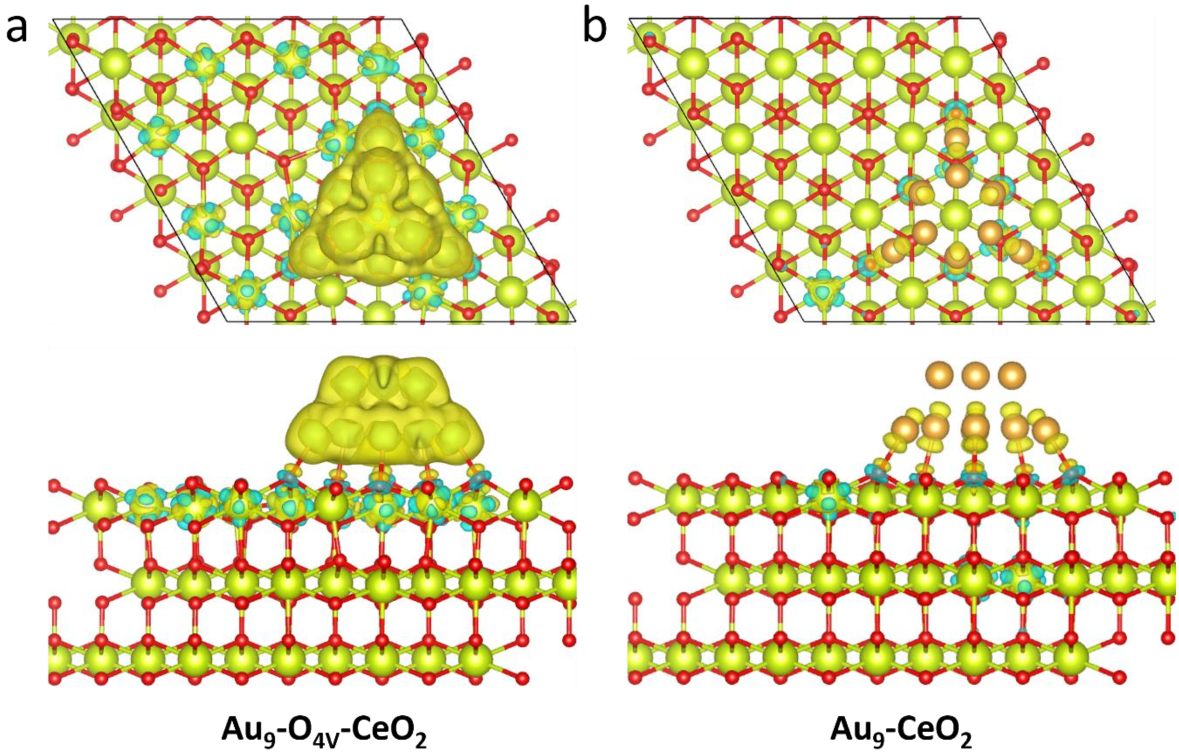

Supplementary Fig. 43 Charge density. Charge density difference showing the charge migration between Au and CeO_2 in (a) Au_9-O_{4V}-CeO_2 and (b) Au_9-CeO_2. The increase and decrease of electron density are marked as blue and yellow, respectively. Ce, O and Au are marked as yellow, red and orange.

The charge density change of Au_9-O_{4V}-CeO_2 was calculated to simulate the charge migration process at the interface of Au and CeO_2 in Au-CeO_2-300 (Supplementary Fig. 43a). The results clearly indicate the decrease in the electron density of Au and the increase in the electron density of CeO_2, which means an electron transportation process from Au to CeO_2. This has provided direct theoretical evidence that Au works as a hole acceptor in Au-CeO_2-300. Similar charge behaviour is also observed over Au_9-CeO_2 in the absence of O_V (Supplementary Fig. 43b).

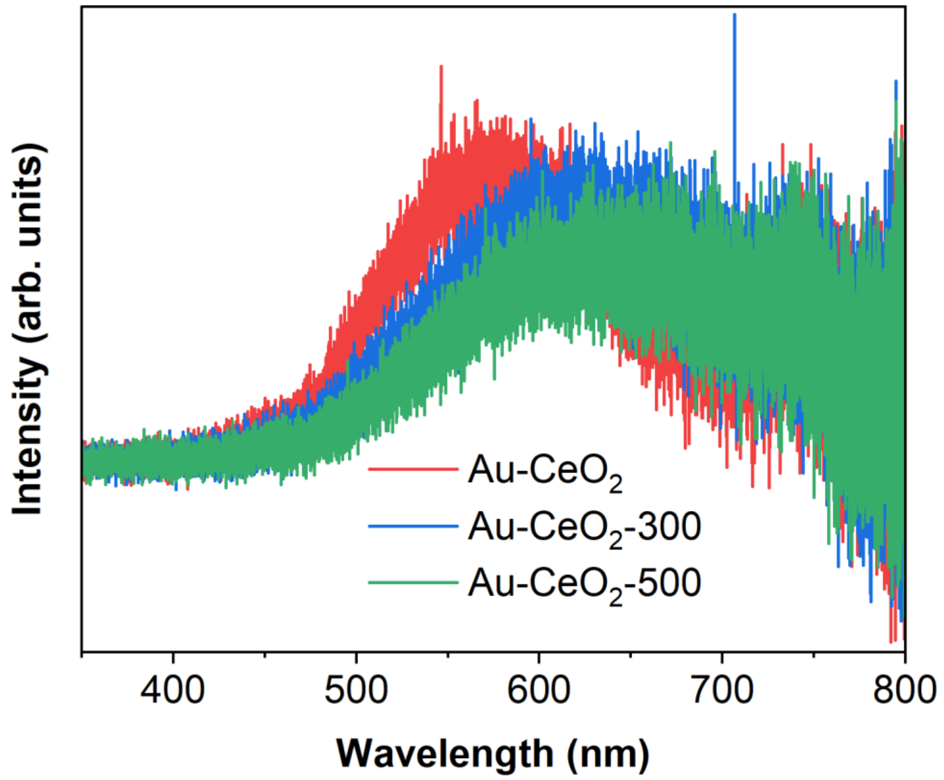

Supplementary Fig. 44 PL spectra of Au-CeO_2, Au-CeO_2-300, and Au-CeO_2-500.

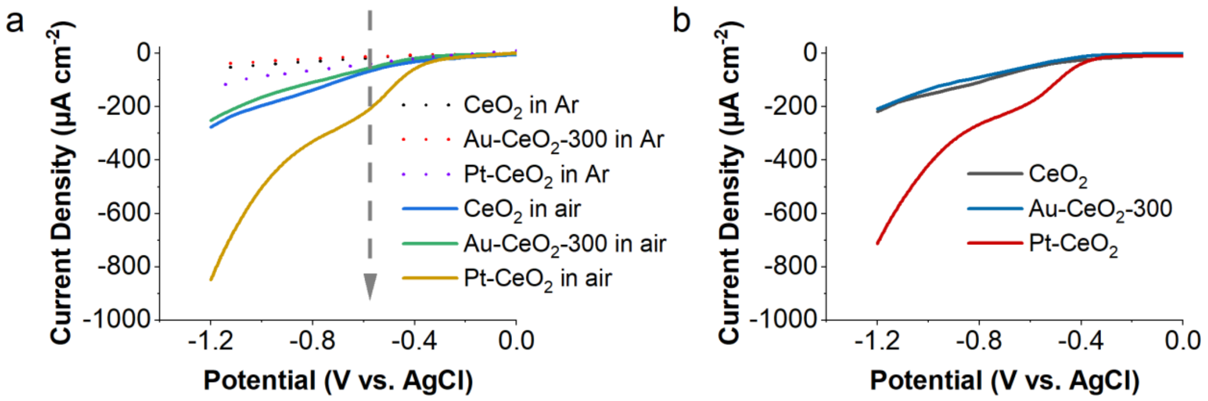

Supplementary Fig. 45 Oxygen reduction reaction. (a) LSV curves of CeO_2, Au-CeO_2-300, and Pt-CeO_2 measured in Na_2SO_4 electrolyte saturated with Ar and Air, (b) O_2 reduction LSV curves obtained from the difference of LSV curves measured in air and Ar over CeO_2, Au-CeO_2-300 and Pt-CeO_2.

To investigate the effect of co-catalysts on oxygen reduction, electrochemical oxygen reduction LSV measurement was carried out. The LSV measurement was undertaken in an electrolyte purged with Ar and then air (Supplementary Fig. 45a). The cathodic current is attributed to the proton reduction (in Ar) and the sum of the oxygen reduction reaction and the proton reduction reaction (in air). The difference between these measurements originates from oxygen reduction (Supplementary Fig. 45b). One can see that Au-CeO_2-300 displays a similar onset potential and reduction current density to CeO_2 for oxygen reduction reaction, suggesting that Au does not promote the reduction half-reaction. In contrast, Pt-CeO_2 displays a much higher reduction current than CeO_2 and Au-CeO_2-300, indicating that Pt as an electron acceptor can significantly promote the oxygen reduction reaction of CeO_2.

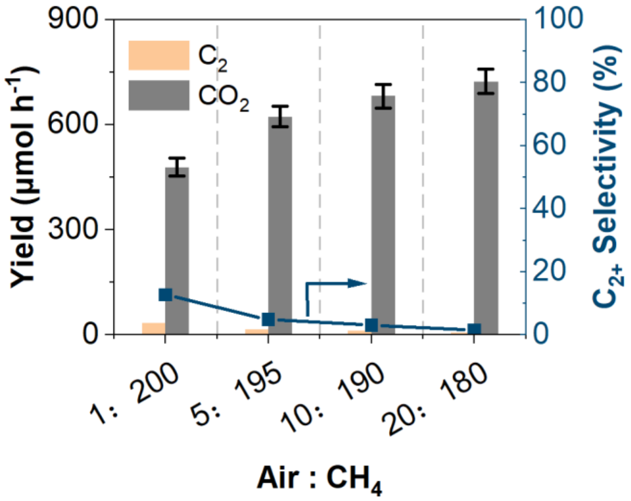

Supplementary Fig. 46 Effects of air to CH_4 ratio. Photocatalytic methane conversion performance of CeO_2 under different Air : CH_4 ratios. Error bars represent standard deviations calculated from the performance tests of the photocatalysts prepared in three different batches. Reaction conditions: 50 mg catalyst, GHSV = 480 000 mL h^{-1} g^{-1}, Pressure = 5 bar, Temperature = 150 °C, 365 nm LED, light intensity = 200 mW cm^{-2}.

The photocatalytic performance of CeO_2 without Au cocatalyst under different air-to-CH_4 ratios was measured. The yield of CO_2 increases significantly from 478 to 722 μmol h^{-1} with the air-to-CH_4 ratio increasing from 1:200 to 20:180, while the selectivity of C_{2+} decreases from 13% to 2%. This suggests that although more superoxide radicals are formed due to more O_2 molecules added, which can increase the overall methane conversion rate, severe overoxidation dominates this process. This displays a different trend in the product selectivity as loading of Au on CeO_2, suggesting that Au does not improve the oxygen reduction reaction by working as an electron acceptor.

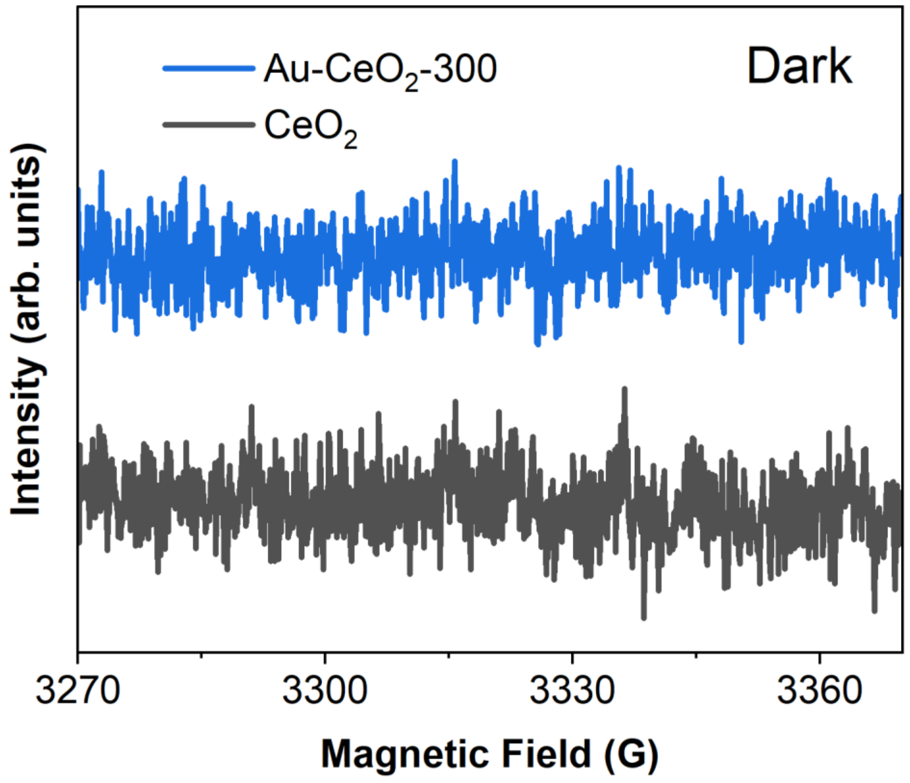

Supplementary Fig. 47 EPR spectra of CeO_2 and Au-CeO_2-300 in dark for O_2^- trapping.

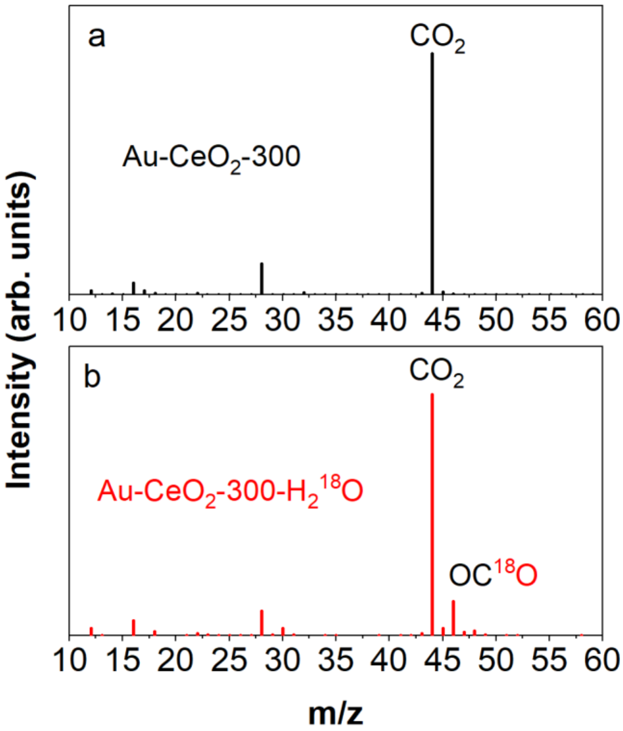

Supplementary Fig. 48 Oxygen exchange. MS spectra of CO_2 formed from photocatalytic OCM over Au-CeO_2-300 (a) without and (b) with ^{18}O doping.

To trace if removal and replenishment of lattice oxygen exists in Au-CeO_2-300 during photocatalytic OCM, ^{18}O was doped into Au-CeO_2-300 by annealing the catalyst in an Ar flow containing $H_2{}^{18}O$ moisture at 300 °C.[34,35] For Au-CeO_2-300 without ^{18}O doping, only non-labelled CO_2 with an m/z ratio of 44 is detected. However, after Au-CeO_2-300 is doped with ^{18}O, a small portion of $CO^{18}O$ is observed. This directly suggests that lattice oxygen participates in the photocatalytic OCM reaction, which is in accordance with the reported.[15,35] Considering the excellent stability of Au-CeO_2-300, the replenishment of lattice oxygen is a facile process during photocatalytic OCM by Au-CeO_2-300.

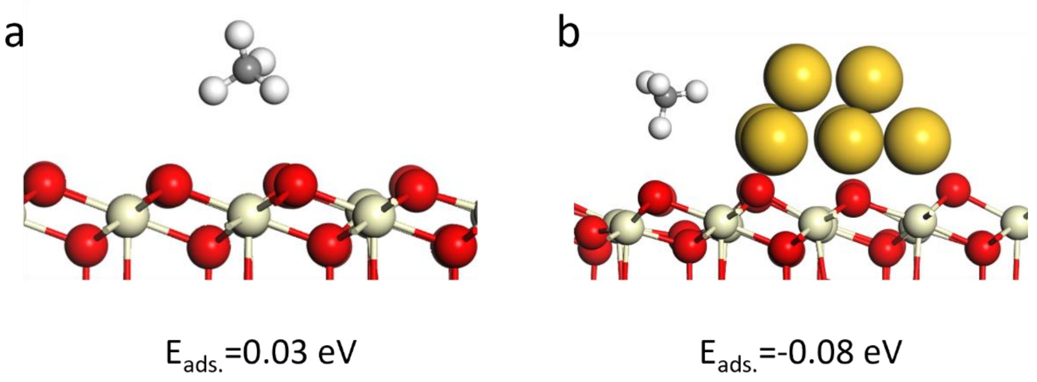

Supplementary Fig. 49 Methane adsorption. Adsorption of CH_4 on CeO_2 (a) and Au-CeO_2-300 (b). Ce, O and Au are marked as white, red and orange.

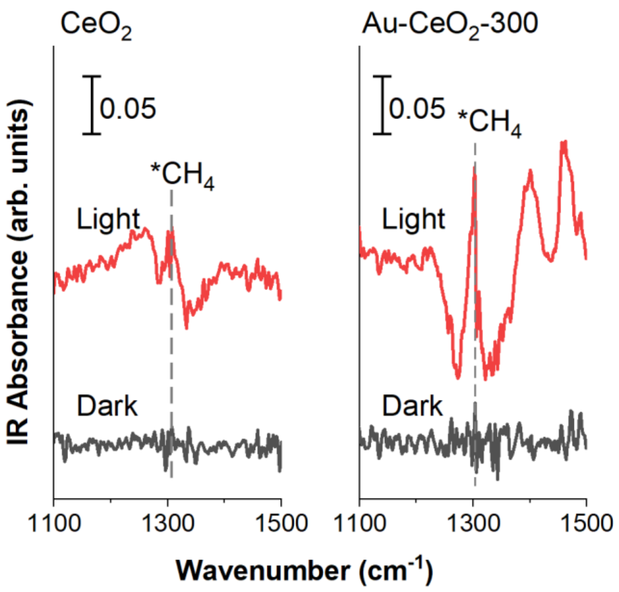

Supplementary Fig. 50 Methane adsorption. *In situ* DRIFTS spectra of CeO_2 and Au-CeO_2-300 in a CH_4:Air of 1:10 atmosphere.

To investigate the effect of light irradiation on the adsorption of methane on the surface of the CeO_2-based catalysts, the *in situ* DRIFTS measurement was performed in an atmosphere with low concentration of methane (CH_4:Air=1:10). A new band at 1308 cm^{-1} (different from that of gas-phase CH_4, 1301 cm^{-1}) is observed over CeO_2 under light-irradiation, originating from surface adsorbed methane. This indicates that light to some extent promotes the adsorption of methane on the surface of CeO_2. The intensity of this band is much stronger over Au-CeO_2-300 compared to CeO_2. This indicates that Au greatly promotes the adsorption of methane on the surface of CeO_2.

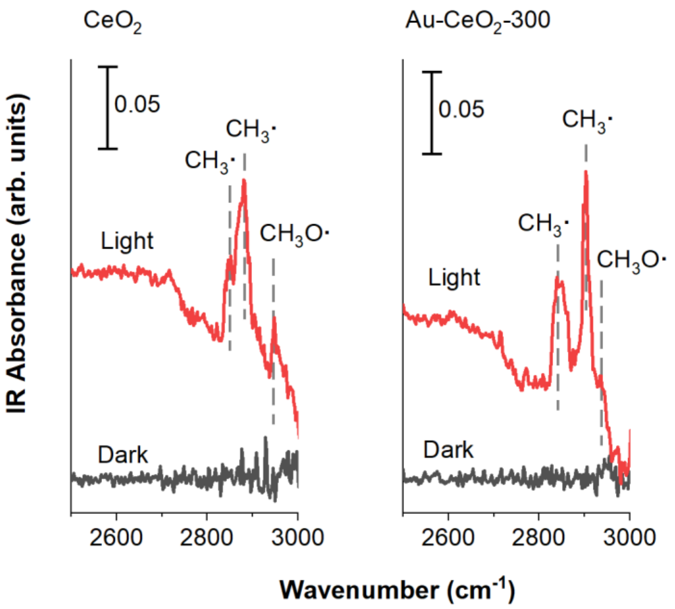

Supplementary Fig. 51 Methane activation. *In situ* DRIFTS spectra of CeO_2 and Au-CeO_2-300 in pure CH_4.

Another feature in the DRIFTS spectra is the band located at 2947 cm^{-1}, originating from the $CH_3O\cdot$ species.[36] The band related to $CH_3O\cdot$ is strong in the spectrum of CeO_2. However, it can hardly be observed in the spectrum of Au-CeO_2-300. After the hole transportation from CeO_2 to Au, the oxidation potential of the photo-hole is reduced. Therefore, the first step of the overoxidation process ($CH_3\cdot$ oxidation to $CH_3O\cdot$) is retarded when Au is loaded onto CeO_2.

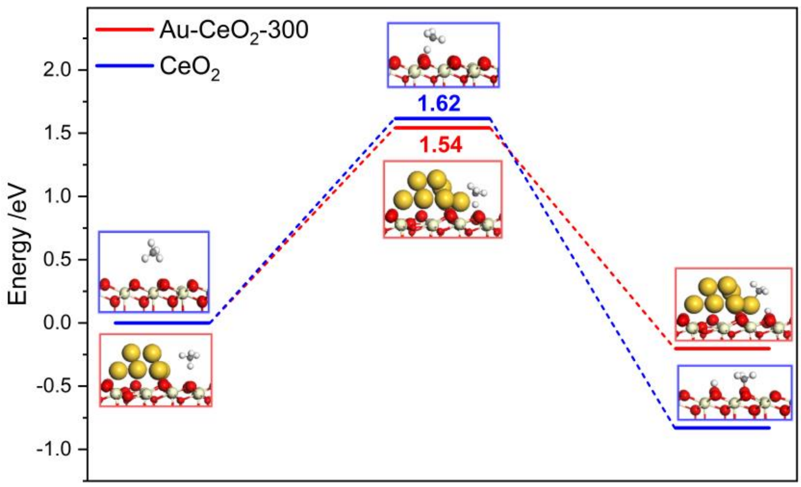

Supplementary Fig 52 Methane activation. Energy profile of CH_4 activation on Au-CeO_2-300 and CeO_2 surfaces. Ce, O, Au, C, and H are marked as light yellow, red, orange, grey, and white, respectively.

On the surface of CeO_2, methane is adsorbed on an O site. After activation, a hydrogen and methyl group each is adsorbed on an O site of CeO_2. An energy barrier of 1.62 eV is observed for methane activation over CeO_2. On the surface of Au-CeO_2-300, methane is adsorbed at the interface of Au and CeO_2. After activation, the methyl group is adsorbed on the Au surface, while hydrogen is adsorbed on the O site of CeO_2. The energy barrier for methane activation over Au-CeO_2-300 is reduced to 1.54 eV. The results indicate that loading Au on CeO_2 reduces the energy barrier for methane activation.

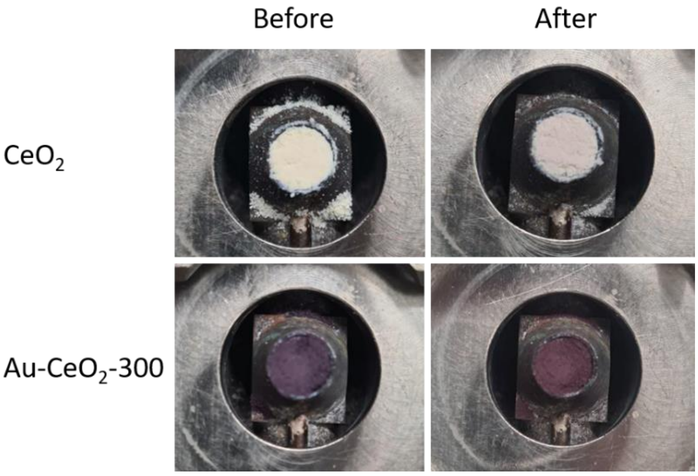

Supplementary Fig. 53 Catalyst reduction. Photos showing the colour change of CeO_2 and Au-CeO_2-300 before and after the *in situ* DRIFTS measurement in pure CH_4 without O_2.

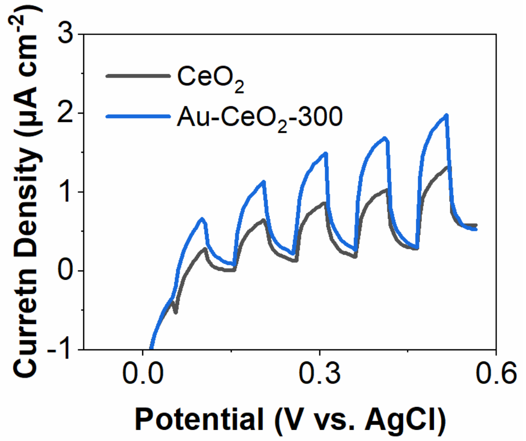

Supplementary Fig. 54 Water oxidation LSV curves of CeO_2 and Au-CeO_2-300.

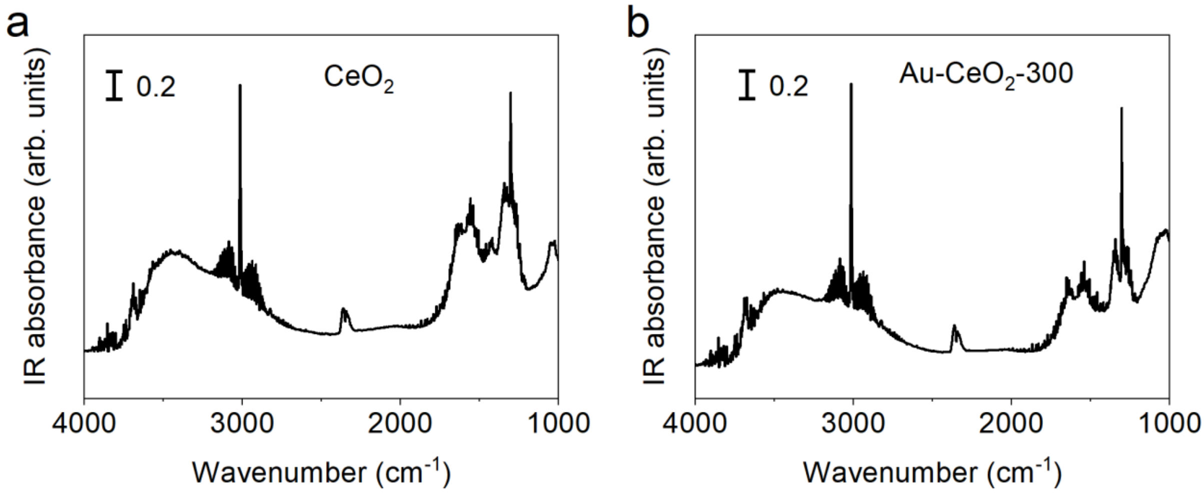

Supplementary Fig. 55 Background of DRIFTS measurement. DRIFTS spectra of (a) CeO_2 and (b) Au-CeO_2-300 in dark (methane to air = 200:1), which were used as the baseline when performing corresponding measurements with light irradiation.

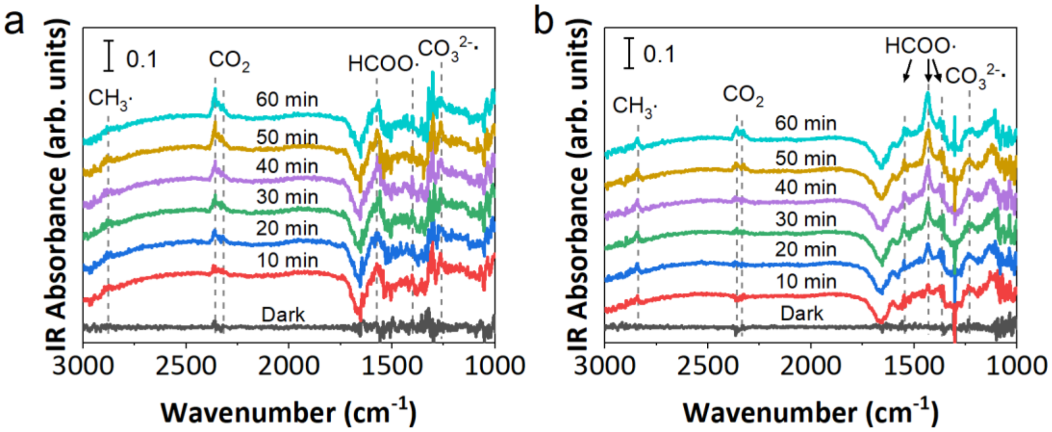

Supplementary Fig. 56 DRIFTS spectra. *In situ* DRIFTS spectra of (a) CeO_2 and (b) Au-CeO_2-300 in dark and under photocatalytic methane oxidation reactions (methane to air = 200:1).

Supplementary Table 5 IR band positions of various species observed by DRIFTS over CeO_2 and Au-CeO_2-300 (N.D = not detected).

Species	Wavenumber (cm^{-1})	
	CeO_2	Au-CeO_2-300
CH_4 (gaseous)	3015/1304	3015/1304
$CH_3\cdot$	2885	2843
CO_2 (gaseous)	2360	2360
$HCOO\cdot$	1566	1548
$HCOO\cdot$	N.D.	1433
$HCOO\cdot$	1402	1373
$CO_3^{2-}\cdot$	1263	1236

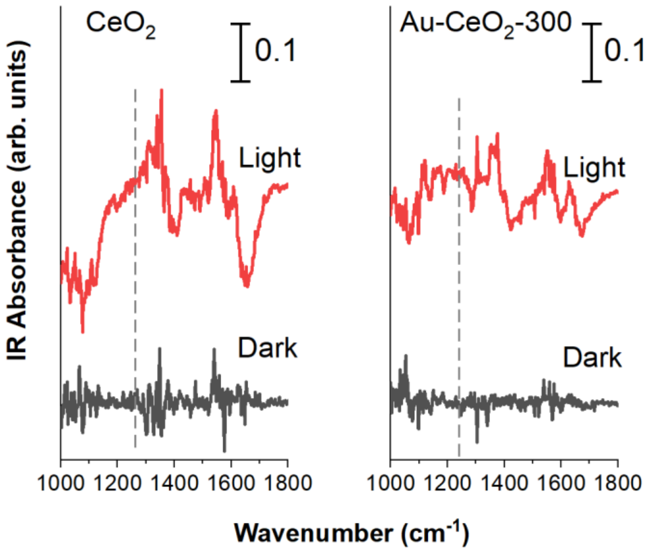

Supplementary Fig. 57 Carbonate species. *In situ* DRIFTS OF CeO_2 and Au-CeO_2-300 under reaction conditions with CH_4 to Air of 1:1.

For CeO_2, no evident IR band is observed under light irradiation, possibly due to the fast transition of carbonate species to carbon dioxide. For Au-CeO_2-300 which displays a relatively sluggish overoxidation process, a broad and strong band at around 1263 cm^{-1} is observed, which is attributed to carbonate species.[36]

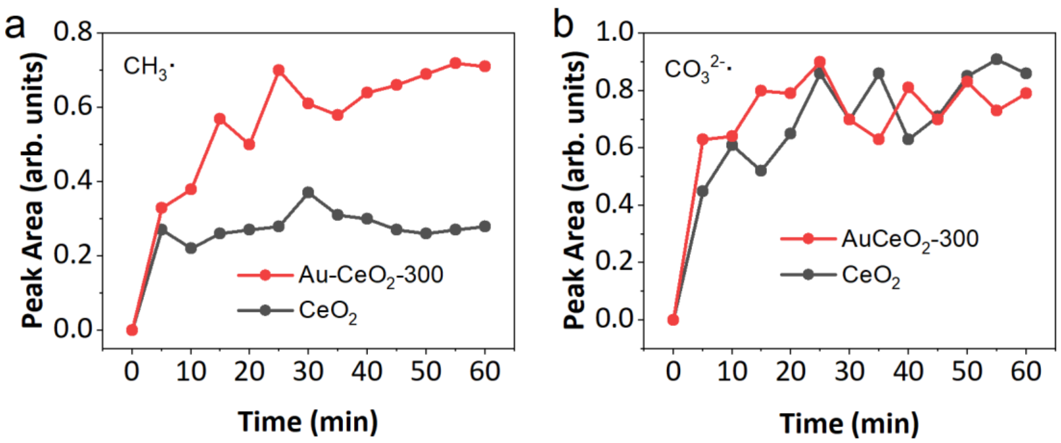

Supplementary Fig. 58 Intermediates evolution. Evolution of (a) $CH_3\cdot$ and (b) $CO_3^{2-}\cdot$ during in situ DRIFTS measurement of CeO_2 and Au-CeO_2-300.

Both CeO_2 and Au-CeO_2-300 display similar evolution features for carbonates. The level of this species quickly reaches a certain level and remains constant subsequently (Supplementary Fig. 58b). It suggests the formation and consumption of the carbonate species are at a similar rate on both photocatalysts.

Supplementary Table 6 Adsorption energy of methyl group at different sites on CeO_2 and Au-CeO_2-300 in the Unit of eV.

Sample	CeO_2 site	Au site
CeO_2	-2.25	--
Au-CeO_2-300	-1.96	-1.71

The adsorption energies are calculated using:

$E_{ads} = E_{CH_3-ads} - E_{Sam} - E_{CH_3}$, Where E_{CH_3-ads}, E_{Sam} and E_{CH_3} are the optimized energies of the methyl group adsorbed on the CeO_2 and Au sites of Au-CeO_2-300 and CeO_2, the Au-CeO_2-300 and CeO_2 surface, and the methyl group, respectively.

The lowest adsorption energy of -2.25 eV is obtained over CeO_2, indicating strong adsorption of methyl radicals on the surface of CeO_2. After loading Au onto CeO_2, the adsorption of methyl on the CeO_2 site changes to a less negative value of -1.96 eV. The Au site of Au-CeO_2-300 displays the weakest adsorption energy of -1.71 eV. The results indicate that methyl groups adsorbed on CeO_2 is more likely to undergo overoxidation due to the largest adsorption energy. The introduction of Au onto CeO_2 weakens the adsorption of methyl groups on the surface of CeO_2 and the Au site on Au-CeO_2-300 has the weakest interaction with the methyl groups. The weak adsorption energy is beneficial for the desorption and/or coupling of methyl radicals and the production of C_{2+} chemicals.

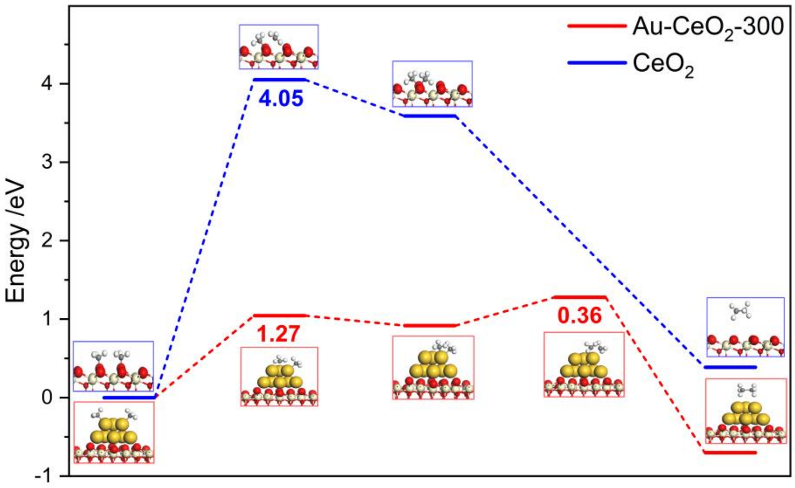

Supplementary Fig. 59 C-C coupling. Energy profile of CH_3· coupling on Au-CeO_2-300 and CeO_2 surface. Ce, O, Au, C, and H are marked as light yellow, red, orange, grey, and white, respectively.

It takes two steps for the coupling of two methyl groups into an ethane molecule. The first step is for one of the methyl groups adsorbed on the adjacent sites of the catalyst to approach the other. This step involves the desorption and transfer of one methyl group, and results in the adsorption of two methyl groups at one atomic site of the catalyst surface. The second step is the coupling of two methyl groups into ethane and the desorption of ethane from the catalyst surface. On the CeO_2 surface, two methyl groups are adsorbed on two adjacent O atoms. A huge energy barrier of 4.05 eV is observed for the transfer of one methyl group. This energy barrier is reduced to as low as 1.27 eV on the Au surface of Au-CeO_2-300. It suggests that the transfer of methyl groups on the surface of Au is much easier than that on CeO_2. Another small energy barrier of 0.36 eV is observed for the coupling of methyl radicals into ethane on the Au surface. Overall, the coupling of methyl groups is limited by the first step, i.e., transfer of the first methyl group, and this step is greatly facilitated after CeO_2 is modified by Au.

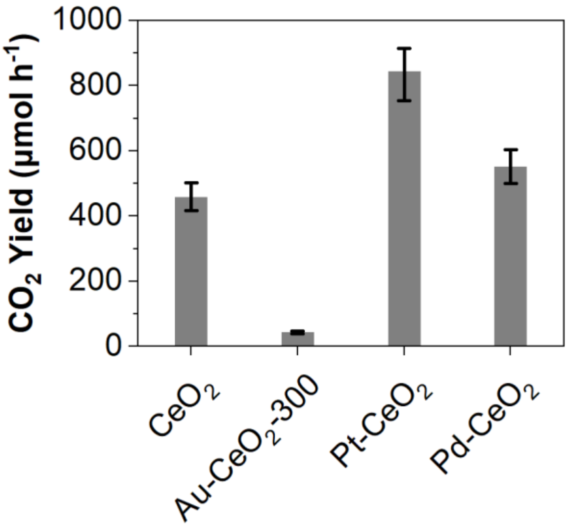

Supplementary Fig. 60 HCOO* conversion. CO_2 production rate from photocatalytic HCOONa oxidation by CeO_2, Au-CeO_2-300, Pt-CeO_2, and Pd-CeO_2. Error bars represent standard deviations calculated from the performance tests of the photocatalysts prepared in three different batches.

Au working as a hole acceptor reduces the oxidation potential of photoholes from CeO_2. Therefore, the oxidation of HCOO· over Au-CeO_2-300 is reduced. In contrast, Pt and Pd, working as electron acceptors, promote the charge separation and then oxidation reaction during photocatalysis. Thus, the production rate of CO_2 from HCOONa oxidation over Pd-CeO_2 and Pt-CeO_2 is significantly improved compared with CeO_2.

Supplementary References

1. Teng, Z. *et al.* Atomically dispersed antimony on carbon nitride for the artificial photosynthesis of hydrogen peroxide. *Nat. Catal.* **4**, 374–384 (2021).

2. Richards, T. *et al.* A residue-free approach to water disinfection using catalytic in situ generation of reactive oxygen species. *Nat. Catal.* **4**, 575–585 (2021).

3. Kresse, G. & Furthmüller, J. Efficient iterative schemes for ab initio total-energy calculations using a plane-wave basis set. *Phys. Rev. B* **54**, 169–186 (1996).

4. Kresse, G. & Furthmüller, J. Efficiency of ab-initio total energy calculations for metals and semiconductors using a plane-wave basis set. *Comput. Mater. Sci.* **6**, 15–50 (1996).

5. Perdew, J. P., Burke, K. & Ernzerhof, M. Generalized gradient approximation made simple. *Phys. Rev. Lett.* **77**, 3865–3868 (1996).

6. Kresse, G. & Joubert, D. From ultrasoft pseudopotentials to the projector augmented-wave method. *Phys. Rev. B - Condens. Matter Mater. Phys.* **59**, 1758–1775 (1999).

7. Anisimov, V. I., Aryasetiawan, F. & Lichtenstein, A. I. First-principles calculations of the electronic structure and spectra of strongly correlated systems: The LDA + U method. *J. Phys. Condens. Matter* **9**, 767–808 (1997).

8. Dudarev, S. & Botton, G. Electron-energy-loss spectra and the structural stability of nickel oxide: An LSDA+U study. *Phys. Rev. B - Condens. Matter Mater. Phys.* **57**, 1505–1509 (1998).

9. Hannes, J., Mills, G. & Jacobsen W Karsten. Nudged elastic band method for finding minimum energy paths of transitions. In Classical and Quantum Dynamics in Condensed Phase Simulations. *World Sci.* 385–404 (1998).

10. Henkelman, G., Uberuaga, B. P. & Jónsson, H. Climbing image nudged elastic band method for finding saddle points and minimum energy paths. *J. Chem. Phys.* **113**, 9901–9904 (2000).

11. Henkelman, G. & Jónsson, H. Improved tangent estimate in the nudged elastic band method for finding minimum energy paths and saddle points. *J. Chem. Phys.* **113**, 9978–9985 (2000).

12. Song, H. *et al.* Selective Photo-oxidation of Methane to Methanol with Oxygen over Dual-Cocatalyst-Modified Titanium Dioxide. *ACS Catal.* **10**, 14318–14326 (2020).

13. Fang, F. *et al.* TiO2 Facet-dependent reconstruction and photocatalysis of CuOx/TiO2 photocatalysts in CO2 photoreduction. *Appl. Surf. Sci.* **564**, (2021).

14. Li, R. *et al.* Spatial separation of photogenerated electrons and holes among {010} and {110} crystal facets of BiVO 4. *Nat. Commun.* **4**, (2013).

15. Song, S. *et al.* A selective Au-ZnO/TiO2 hybrid photocatalyst for oxidative coupling of methane to ethane with dioxygen. *Nat. Catal.* **4**, 1032–1042 (2021).

16. Zhang, W. *et al.* High-performance photocatalytic nonoxidative conversion of methane to ethane and hydrogen by heteroatoms-engineered TiO2. *Nat. Commun.* **13**, 1–9 (2022).

17. Wang, C. *et al.* Synergy of Ag and AgBr in a Pressurized Flow Reactor for Selective Photocatalytic Oxidative Coupling of Methane. *ACS Catal.* **13**, 3768–3774 (2023).

18. Amano, F., Akamoto, C., Ishimaru, M., Inagaki, S. & Yoshida, H. Pressure-induced dehydrogenative coupling of methane to ethane by platinum-loaded gallium oxide photocatalyst. *Chem. Commun.* **56**, 6348–6351 (2020).

19. Ishimaru, M., Amano, F., Akamoto, C. & Yamazoe, S. Methane coupling and hydrogen evolution induced by palladium-loaded gallium oxide photocatalysts in the presence of water vapor. *J. Catal.* **397**, 192–200 (2021).

20. Li, X., Xie, J., Rao, H., Wang, C. & Tang, J. Platinum- and CuOx-Decorated TiO2 Photocatalyst for Oxidative Coupling of Methane to C2 Hydrocarbons in a Flow Reactor. *Angew. Chemie - Int. Ed.* **59**, 19702–19707 (2020).

21. Zhang, J. *et al.* Efficiently Light-Driven Nonoxidative Coupling of Methane on Ag/NaTaO 3 : A Case for Molecular-Level Understanding of the Coupling Mechanism . *ACS Catal.* 2094–2105 (2023) doi:10.1021/acscatal.2c05081.

22. Yu, X. *et al.* Stoichiometric methane conversion to ethane using photochemical looping at ambient temperature. *Nat. Energy* **5**, 511–519 (2020).

23. Jiang, W. *et al.* Pd-Modified ZnO-Au Enabling Alkoxy Intermediates Formation and Dehydrogenation for Photocatalytic Conversion of Methane to Ethylene. *J. Am. Chem. Soc.* **143**, 269–278 (2021).

24. Singh, S. P. *et al.* A Pd-Bi Dual-Cocatalyst-Loaded Gallium Oxide Photocatalyst for Selective and Stable Nonoxidative Coupling of Methane. *ACS Catal.* **11**, 13768–13781 (2021).

25. Wang, G. *et al.* Light-Induced Nonoxidative Coupling of Methane Using Stable Solid Solutions. *Angew. Chemie - Int. Ed.* **60**, 20760–20764 (2021).

26. Amano, F. & Ishimaru, M. Hydroxyl Radical Formation on Metal-Loaded Ga2O3Photocatalysts for Dehydrogenative Coupling of Methane to Ethane with Water. *Energy and Fuels* **36**, 5393–5402 (2022).

27. Huang, A. *et al.* Room-temperature coupling of methane with singlet oxygen†. *Environ. Sci. Adv.* **1**, 438–442 (2022).

28. Jiang, D. *et al.* Dynamic and reversible transformations of subnanometre-sized palladium on ceria for efficient methane removal. *Nat. Catal.* **6**, (2023).

29. Goodman, E. D. *et al.* Catalyst deactivation via decomposition into single atoms and the role of metal loading. *Nat. Catal.* **2**, 748–755 (2019).

30. Jeong, H. *et al.* Highly durable metal ensemble catalysts with full dispersion for automotive applications beyond single-atom catalysts. *Nat. Catal.* **3**, 368–375 (2020).

31. Jin, J. *et al.* Anchoring ultrafine metallic and oxidized Pt nanoclusters on yolk-shell TiO2 for unprecedentedly high photocatalytic hydrogen production. *Nano Energy* **38**, 118–126 (2017).

32. Scanlon, D. O. *et al.* Band alignment of rutile and anatase TiO2. *Nat. Mater.* **12**, 798–801 (2013).

33. Wang, P. *et al.* Selective Photocatalytic Oxidative Coupling of Methane via Regulating Methyl Intermediates over Metal/ZnO Nanoparticles. *Angew. Chemie* **135**, (2023).

34. Watanabe, R. *et al.* Role of alkali metal in a highly active Pd/alkali/Fe2O 3 catalyst for water gas shift reaction. *Appl. Catal. A Gen.* **457**, 1–11 (2013).

35. Shoji, S. *et al.* Photocatalytic uphill conversion of natural gas beyond the limitation of thermal reaction systems. *Nat. Catal.* **3**, 148–153 (2020).

36. Fu, C. *et al.* Spontaneous Bulk-Surface Charge Separation of TiO2-{001} Nanocrystals Leads to High Activity in Photocatalytic Methane Combustion. *ACS Catal.* **12**, 6457–6463 (2022).
